# 5‐methyl‐2‐carboxamidepyrrole‐based novel dual mPGES‐1/sEH inhibitors as promising anticancer candidates

**DOI:** 10.1002/ardp.202400708

**Published:** 2024-12-18

**Authors:** Ester Colarusso, Gianluigi Lauro, Marianna Potenza, Paola Galatello, Maria Luisa d'Aulisio Garigliota, Maria Grazia Ferraro, Marialuisa Piccolo, Maria Giovanna Chini, Carlo Irace, Pietro Campiglia, Robert Klaus Hoffstetter, Oliver Werz, Anna Ramunno, Giuseppe Bifulco

**Affiliations:** ^1^ Department of Pharmacy University of Salerno Fisciano Italy; ^2^ Department of Pharmaceutical/Medicinal Chemistry, Institute of Pharmacy Friedrich Schiller University Jena Germany; ^3^ Department of Molecular Medicine and Medical Biotechnologies, School of Medicine and Surgery University of Naples Naples Italy; ^4^ BioChem Lab, Department of Pharmacy, School of Medicine and Surgery University of Naples Naples Italy; ^5^ Department of Biosciences and Territory University of Molise Pesche Italy

**Keywords:** 5‐methyl‐2‐carboxamidepyrrole‐based compounds, cancer, docking experiments, mPGES‐1, sEH

## Abstract

Inhibiting microsomal prostaglandin E_2_ synthase‐1 (mPGES‐1), an inducible enzyme involved in prostaglandin E_2_ (PGE_2_) biosynthesis and tumor microenvironment (TME) homeostasis, is a valuable strategy for treating inflammation and cancer. In this work, 5‐methylcarboxamidepyrrole‐based molecules were designed and synthesized as new compounds targeting mPGES‐1. Remarkably, compounds **1f**, **2b**, **2c**, and **2d** were able to significantly reduce the activity of the isolated enzyme, showing IC_50_ values in the low micromolar range. With the aim of further profiling the synthesized molecules, their ability to interfere with the activity of soluble epoxide hydrolase (sEH), whose inhibition blocks the loss of the anti‐inflammatory mediators epoxyeicosatrienoic acids (EETs or epoxyicosatrienoic acids), was investigated in silico and by employing specific biological assays. Among the set of tested compounds, **1f**, **2b**, **2c**, and **2d** emerged as mPGES‐1/sEH dual inhibitors. Moreover, given that overexpression of mPGES‐1 has been observed in many human tumors, we finally explored the biological effect of our compounds in an in vitro model of human colorectal cancer (CRC). The obtained outcomes pave the way for future investigation to optimize and further characterize anticancer pharmacological profile of the carboxamidepyrrole‐based molecules.

## Graphical abstract

5‐Methyl‐2‐carboxamidepyrrole‐based compounds were disclosed as new microsomal prostaglandin E_2_ synthase‐1 (mPGES‐1)/soluble epoxide hydrolase (sEH) inhibitors, highlighting their promising profile as anticancer agents. A combined in silico/experimental approach is reported, highlighting its applicability for discovering novel bioactive compounds against specific targets of interest.

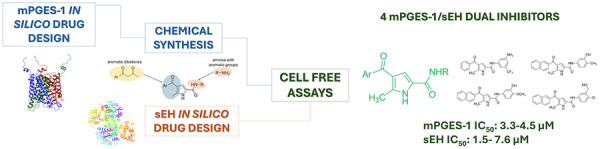

**Abbreviations**
DCM: dichloromethane
DMAP: 4‐(dimethylamino)pyridine
DMF: N,N‐dimethylformamide
HaCaT: high sensitivity of human epidermal keratinocytes
HBTU: N,N,N,N'‐tetramethyl‐O‐(1H‐benzotriazol‐1‐yl)uronium hexafluorophosphate
HOBT: 1‐hydroxybenzotriazole
MTT: 3‐(4,5‐dimethyl‐2‐thiazolyl) 2,5‐diphenyl‐2‐H‐tetrazolium bromide
NMM: N‐methylmorpholine
RP‐LC: reversed‐phase liquid chromatography
RPMI: Roswell Park Memorial Institute
TEA: triethylamine
TFA: trfluoroacetic acid.

## 1 INTRODUCTION

Inflammation and cancer diseases are recognized causes of death worldwide, and their interplay has been widely demonstrated.^[^ ^1^, ^2^ ^]^ Chronic and uncontrolled inflammatory conditions, in fact, are usually able to trigger tumor development and progression by perturbing microenvironment homeostasis, recruiting protumorigenic cells, and increasing the production of tumoral molecular mediators.^[^ ^3^, ^4^ ^]^ Specifically, the role of inflammation‐related prostaglandins yields several mechanisms for tumor growth progression and immune system evasion,^[^ ^3^ ^]^ especially for tumors containing tumor‐infiltrating lymphocytes (TIL).^[^ ^5^ ^]^

Various inflammatory mediators such as prostaglandins, prostacyclin, lipoxins, and leukotrienes are biosynthesized from arachidonic acid (ARA), an omega‐6 polyunsaturated fatty acid (PUFA), through the action of three classes of enzymes, namely cyclooxygenases (COXs), lipoxygenases (LOXs), and cytochrome P450s (CYPs). In this field, prostaglandin E_2_ (PGE_2_) was reported as one of the most relevant mediators in inflammation and cancer evolution.^[^ ^6^, ^7^ ^]^ Indeed, preclinical outcomes showed the selective reduction of PGE_2_ as a key factor leading to important implications in the inflammatory response.^[^ ^8^, ^9^ ^]^ The use of nonsteroidal anti‐inflammatory drugs (NSAIDs), chemical agents able to inhibit COXs, is widely considered a well‐known pharmacological strategy for shutting down inflammatory symptoms.^[^ ^10^ ^]^ However, side effects caused by NSAIDs,^[^ ^11^, ^12^ ^]^ such as cardiovascular and gastrointestinal, represent a relevant drawback that prompted the interest of researchers in disclosing novel valuable therapeutic targets. In this scenario, inhibiting PGE_2_ biosynthesis while simultaneously avoiding the reduction of the other prostaglandin (PGI_2_, PGD_2_, PGF_2_, TxA_2_) levels may be a promising strategy for blocking inflammation and interfering with cancer‐related disease progression. Specifically, three different isoforms of PGE_2_ synthases, downstream of COXs, are directly involved in PGE_2_ biosynthesis: cPGES‐1 (cytosolic) and mPGES‐2 (microsomal) that are constitutively expressed, and mPGES‐1 that is instead induced in pathological conditions. The aforementioned implication of PGE_2_ in the development of several types of cancer and inflammatory response, as well as “classic” NSAIDs side effects, arising from the low selectivity in reducing the concentration of all prostaglandins, prompted us to continue investigating the discovery of new molecular entities able to inhibit mPGES‐1, which may provide several improvements in cancer and inflammation diseases treatment.^[^ ^13^ ^]^ In the last few years, new chemical platforms targeting mPGES‐1 were developed by us^[^ ^14^, ^15^, ^16^, ^17^, ^18^, ^19^, ^20^, ^21^, ^22^, ^23^, ^24^, ^25^ ^]^ and others,^[^ ^26^, ^27^, ^28^, ^29^, ^30^, ^31^ ^]^ and, to date, only two inhibitors entered clinical development phases, namely GRC27864 (Glenmark Pharmaceuticals Ltd) and LY3023703 (developed by Eli Lilly). For the latter, the approval process was interrupted due to its liver toxicity.^[^ ^13^ ^]^

Furthermore, interfering with the activity of soluble epoxide hydrolase (sEH) represents a valuable option for discovering novel anti‐inflammatory/anticancer compounds as well. Specifically, sEH is responsible for epoxyeicosatrienoic acids (also known as EETs or epoxyicosatrienoic acids) conversion to the corresponding dihydroxyeicosatrienoic acids (DHETs or DiHETrEs or dihydroxyicosatrienoic acids), leading to the lack of the biological benefits associated with the former.^[^ ^13^ ^]^ Also, sEH inhibition was associated with the decreased plasma levels of proinflammatory cytokines and nitric oxide metabolites, promoting the formation of lipoxins and supporting the resolution of inflammation, and targeting sEH was commonly recognized for preventing inflammation‐induced carcinogenesis.^[^ ^32^ ^]^ Indeed, recent studies confirmed that sEH is also increased in samples of colonic dysplasia and adenocarcinoma from colorectal cancer (CRC) patients.^[^ ^27^, ^28^ ^]^ When sEH is inhibited, fatty acid epoxides were demonstrated to be stabilized and to have stronger anti‐inflammatory effects in colitis, pancreatitis, and CRC.

In light of these considerations, the development of dual mPGES‐1/sEH inhibitors could result in promising therapeutic option featuring reduced side effects. Such agents could work on both targets with the main advantage of synergistic effects that reduce the dose, preventing resistance and adverse effects of drugs.^[^ ^16^, ^23^, ^30^, ^31^, ^33^ ^]^

In this work, the pyrrole moiety was investigated for developing novel anti‐inflammatory/anticancer agents. Pyrrole, in fact, is present in a broad number of drugs presenting specific pharmacological profiles, such as antibacterial,^[^ ^34^, ^35^ ^]^ anti‐inflammatory,^[^ ^36^ ^]^ antifungal,^[^ ^34^ ^]^ and anticancer.^[^ ^37^ ^]^ Several bioactive pyrrole‐based molecules were developed over the years, making this core an interesting heterocycle for medicinal chemistry investigation.^[^ ^38^ ^]^ Interestingly, in only one previous study by Lehr's group,^[^ ^29^ ^]^ pyrrole alkanoic acid derivatives, previously discovered as anti‐inflammatory agents targeting cytosolic phospholipase A_2_,^[^ ^39^, ^40^, ^41^ ^]^ apparently emerged as new mPGES‐1 inhibitors but they later proved to be nuisance mPGES‐1 inhibitors due to high lipophilicity that caused aggregate formation. Consequently, they were considered as false positives. Starting from these data, we here wondered whether the pyrrole core could be re‐evaluated on mPGES‐1 by molecular docking experiments to drive oriented chemical substitutions. The here reported in silico experimental workflow finally led to the identification of new mPGES‐1 inhibitors, and further subsequent investigations allowed to disclose their ability to inhibit sEH, thus identifying unprecedented mPGES‐1/sEH dual inhibitors that showed promising anticancer activities against CRC cell lines.

## 2 RESULTS AND DISCUSSION

### 2.1 Design and selection of mPGES‐1 inhibitors

**Figure 1:**
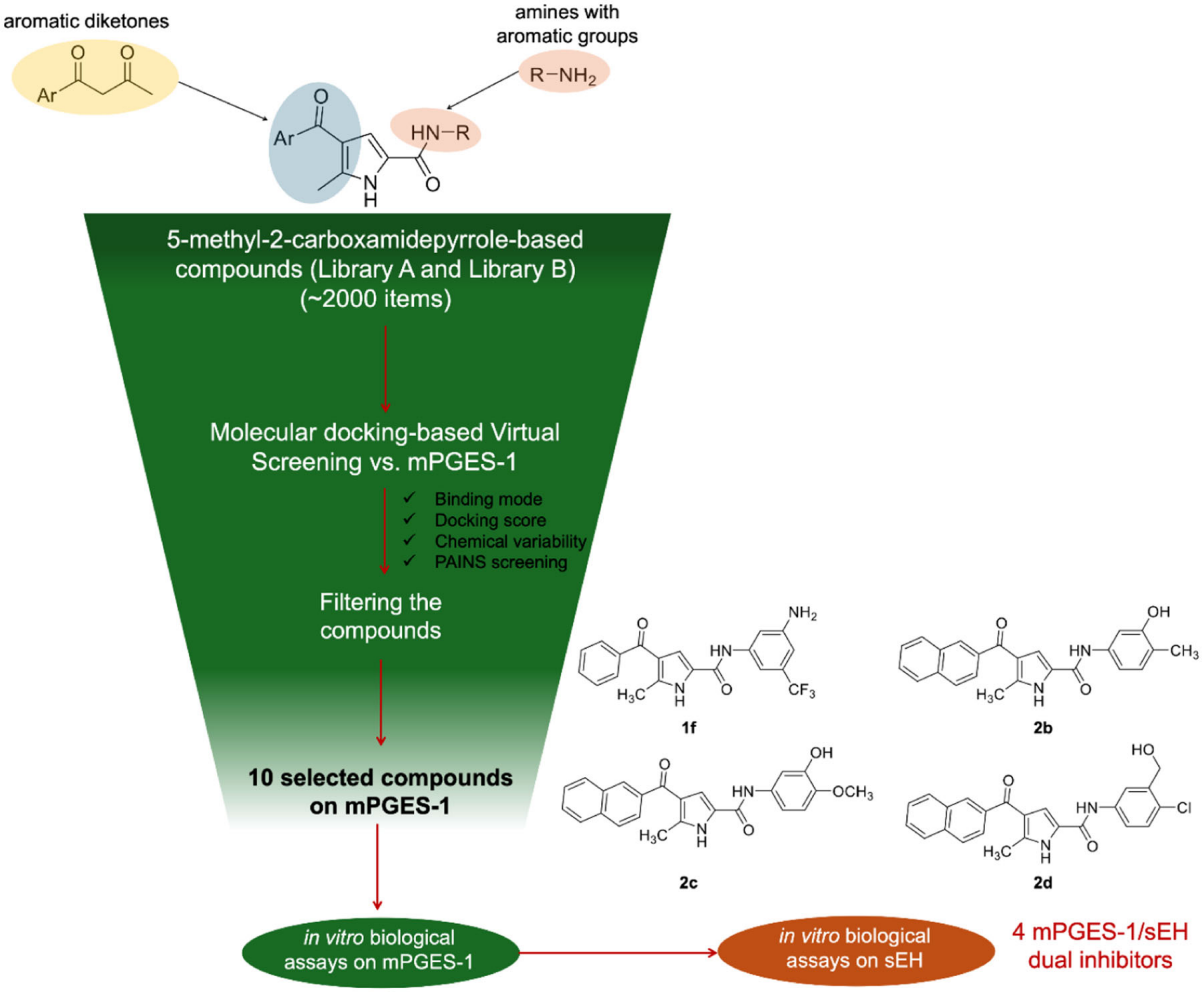
Workflow for the in silico generation of 5‐methyl‐2‐carboxamidepyrrole‐based microsomal prostaglandin E_2_ synthase‐1 (mPGES‐1)/soluble epoxide hydrolase (sEH) dual inhibitors.

In an article published in 2012, a series of pyrrole alkanoic acids was reported as “nuisance inhibitors” of mPGES‐1 (Supporting Information S2: Table S1).^[^ ^29^ ^]^ Further, a new series of lipophilic carboxylic acids showed the same biological behavior on the mPGES‐1 enzyme.^[^ ^21^ ^]^ Starting from these premises and prompted by the aim of discovering novel mPGES‐1 inhibitors, we wondered whether the pyrrole scaffold, if properly decorated, could still represent a valuable moiety for developing new modulators of this enzyme. With this aim, we virtually built in silico two combinatorial libraries comprising pyrrole‐based molecules differently functionalized and potentially synthesizable according to the chosen synthetic route (Figure 1) (vide infra).

Specifically, considering the presence of aromatic residues placed in two different positions onto the mPGES‐1 binding site, that is, Tyr130_chainA_ and Phe44_chainB_/His53_chainB_ which could be targeted through π–π interactions, a first aromatic moiety was inserted at position 4 instead of the aliphatic ketone chain of compound **I** (Supporting Information S2: Table S1). Also, the carboxylic function was replaced with an amidic moiety, which was demonstrated to be an advantageous strategy when designing anti‐inflammatory agents endowed with reduced side effects.^[^ ^42^ ^]^ Such amidic group was also functionalized with a second aromatic substituent to gain further π–π interactions with the protein counterpart (vide infra). Moreover, the methyl group was removed from the nitrogen on the pyrrolic ring to establish H‐bonds with polar amino acids present in the binding site of mPGES‐1 (Ser127_chainA_, Thr131_chainA_, Tyr130_chainA_, His53_chainB_, Gln36_chainB,_ Thr28_chainB_). This allowed us to finally select a small library of 10 new 5‐methyl‐2‐carboxamidepyrrole‐based compounds (**1a**–**f** and **2a**–**d**, Table 1) as novel anticancer agents (vide infra).

**Table 1:** Chemical structure of the selected and synthesized compounds **1a–f** and **2a–d**.

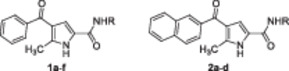	
Compound	R
**1a**	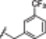
**1b**	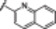
**1c**	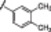
**1d**	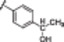
**1e**	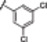
**1f**	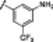
**2a**	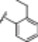
**2b**	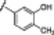
**2c**	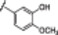
**2d**	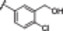

**Scheme 1:**
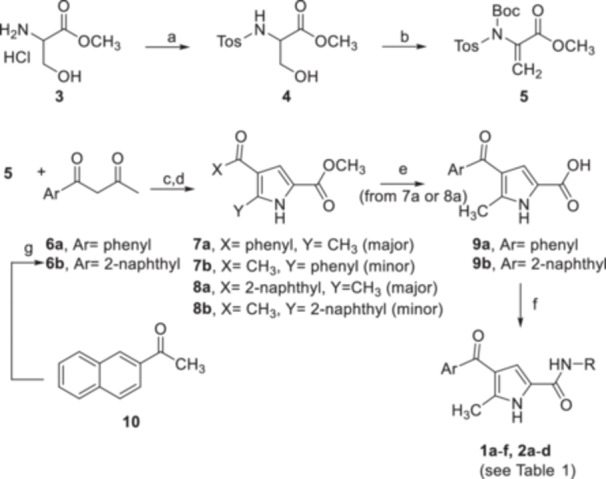
Synthetic strategy applied for the synthesis of compounds **1a**–**f** and **2a**–**d**. Reagents and conditions: (a) TosCl, TEA, DCM, rt, 12 h; (b) (Boc)_2_O, dry CH_3_CN, rt, 3 h^[^ ^37^, ^43^ ^]^; (c) Cs_2_CO_3_, CH_3_CN, 12 h, rt; (d) TFA (20% v/v in DCM), rt; (e) NaOH 2 M, MeOH, reflux; (f) HOBT, HBTU, NMM, DMF, appropriate amine, rt, 12 h (for **1a**,**1c**,**1d**, **1f**, and **2a–d**), or oxalyl chloride, dry toluene, 2 h, 50°C, then appropriate amine, dry TEA, dry DCM, rt, overnight (for **1b** and **1e)**; (g) dry EtOAc, NaH (60% dispersion in mineral oil), dry DMF, 0°C to rt, 4 h. DCM, dichloromethane; DMAP, 4‐(dimethylamino)pyridine; DMF, N,N‐dimethylformamide; HBTU, *N*,*N*,*N*,*N*’‐tetramethyl‐O‐(1H‐benzotriazol‐1‐yl)uronium hexafluorophosphate; HOBT, 1‐hydroxybenzotriazole; NMM, *N*‐methylmorpholine, TEA, triethylamine.^[^ ^44^ ^]^

In detail, the building of the combinatorial libraries started considering the 2‐carboxamide pyrrole building block (Figure 1). Based on the synthons of the selected chemical route reported in Scheme 1, two large libraries of molecules were prepared, specifically as derivatives of 5‐methyl‐4‐phenoyl‐1*H*‐pyrrole‐2‐carboxamide (Library A) and 5‐methyl‐4‐naphtoyl‐1*H*‐pyrrole‐2‐carboxamide (Library B) (Figure 1 and Table 1). The choice of the two different substituents at C‐4 arose from the commercial availability of 1‐phenylbutane‐1,3‐dione (for Library A) and the possibility of easily synthesizing 1‐(naphthalen‐2‐yl)butane‐1,3‐dione (for Library B) diketone synthons (Scheme 1). In this way, we introduced two aromatic substituents at C‐4 as pilot moieties to investigate the impact of the different dimensions of the phenoyl and naphtoyl in binding the protein counterpart through in silico analysis. Indeed, preliminary molecular docking investigations of 5‐methyl‐4‐phenoyl‐1*H*‐pyrrole‐2‐carboxamide and 5‐methyl‐4‐naphtoyl‐1*H*‐pyrrole‐2‐carboxamide as the two starting partially decorated building blocks highlighted the good accommodation of the aromatic groups in the binding site region, specifically toward the cytoplasmic side and evidenced their ability to establish π–π interactions with Phe44_chainB_/His53_chainB_ (Supporting Information S2: Figure S1). Subsequently, commercially available aromatic amines were used for the decoration of the scaffolds (Figure 1) on the carboxamide moiety at C‐2 to gain further aromatic interactions with Tyr130_chainA_ as well as further contacts with key residues belonging to the binding region toward the endoplasmic reticulum membrane (e.g., Gln134_chainA_, Tyr28_chainB_). With this aim, two libraries, each containing 3097 potentially synthesizable compounds, were built using CombiGlide software.^[^ ^45^ ^]^ LigPrep^[^ ^46^ ^]^ calculations were then performed for predicting and taking into account all tautomers, stereoisomers, and protonation states, and QikProp ^[^ ^47^ ^]^ for the estimation of pharmacokinetic parameters. Specifically, after applying specific filters, “drug‐like” compounds were discriminated from “nondrug‐like” compounds, and molecules with reactive and toxic functional groups were removed, finally leading to Library A (1347 items) and Library B (783 items) (see Section 4). The resulting libraries were then submitted to docking calculations employing the Virtual Screening Workflow (VSW), using Glide software as implemented in the Schrödinger Suite (see Section 4).^[^ ^48^, ^49^, ^50^ ^]^ The final set of selected compounds (see Section 4) was then analyzed, prioritizing the visual inspection and evaluation of the interactions with the protein counterpart and taking into account the chemical variability, synthetical accessibility, and commercial availability of the related synthons (vide infra). Eventually, 10 compounds were selected from Libraries A and B, specifically **1a**–**f** as 4‐phenoyl and **2a**–**d** as 4‐naphthoyl derivatives (Table 1). Among them, compounds **1a** and **2a** were randomly selected as negative control due to their predicted poor binding against mPGES‐1. Also, the selected compounds were processed using the SwissADME ^[^ ^51^ ^]^ web tool to exclude the putative presence of chemical species belonging to the “Pan‐Assay Interference compounds” chemical class. All the molecules passed the filter and were submitted to the subsequent synthetic step.

### 2.2 Chemical synthesis

The synthesis of amides **1a**–**f** and **2a**–**d** was accomplished as depicted in Scheme 1. The dehydroalanine derivative **5** was obtained starting from (±)‐serine methyl ester hydrochloride **3**, which was converted into the corresponding tosyl derivative **4** through a reaction with tosyl chloride. The latter was reacted with (Boc)_2_O (di‐*tert‐*butyl dicarbonate) in the presence of 4‐dimethylaminopyridine to give **5** according to a previously reported procedure.^[^ ^43^ ^]^ Coupling of **5** with the appropriate β‐diketones **6a**‐**b** in the presence of Cs_2_CO_3_ as a base, and subsequent treatment with TFA (20% v/v in DCM) afforded the pyrrole derivatives **7a**,^[^ ^37^ ^]^ **8a** and their regioisomers **7b**,^[^ ^37^ ^]^ **8b** as minor products (Scheme 1). The esters **7a** and **8a** were hydrolyzed in an alkaline medium into the corresponding acids **9ab** which, in turn, were coupled with the appropriate amine in the presence of 1‐hydroxybenzotriazole (HOBT), HBTU *N,N,N’,N’‐*tetramethyl‐O‐(^1^H‐benzotriazol‐1‐yl)uronium hexafluorophosphate (HBTU) as coupling reagents, and NMM (*N*‐methylmorpholine) as a base to produce **1a**,**1c**,**1d**,**1f**, and **2a**–**d** in good yields. Amides **1b** and **1e** were obtained from **9a** and 2‐aminoquinoline or 3,5‐dichloroaniline, respectively, under Schotten–Baumann conditions. Compound **6b** was synthesized via a Claisen‐type condensation reaction between 2‐acetonaphthone **10** and ethyl acetate (EtOAc) in the presence of sodium hydride (NaH) as a base (Scheme 1) following a previously reported procedure.^[^ ^44^ ^]^

### 2.3 Biological assays on mPGES‐1 and sEH and analysis of molecular docking poses

Compounds **1a**–**f** and **2a**–**d** were submitted to a cell‐free assay mPGES‐1^[^ ^52^ ^]^ to validate the inhibitory activity predicted in silico. All the compounds were screened at two different concentrations (10 and 1 µM), with the aim of identifying the candidates able to induce a significant inhibition of PGE_2_ formation. At 10 µM, all the compounds but not **1a**, **1e**, and **2a** showed a moderate to significant interference with mPGES‐1, leading to a residual mPGES‐1 activity at least lower than 70%. Remarkably, compounds **2b**–**d** reduced residual mPGES‐1 activity up to 17.6% at 10 µM, and 65%–75% at 1 µM (Supporting information S2: Figure S52). Thus, the IC_50_ was evaluated for these hit compounds (Table 2), with compound **2b** featuring the highest potency (IC_50 _= 3.3 ± 0.5 µM). Also, compound **1b** led to ~55% residual mPGES‐1 activity at 10 µM. Unfortunately, compound **1d** could not be analyzed due to unspecific interference of the compound with the assay.

**Table 2:** Values of the residual activity of microsomal prostaglandin E_2_ synthase‐1 (mPGES‐1) and human soluble epoxide hydrolase (sEH) after incubation with compounds 1a–f and 2a–d at a concentration of 10 μM.

Compound	Residual activity of mPGES‐1, mean ± SD (%)	IC_50_ (µM), mean ± SD for mPGES‐1	Residual activity of sEH, mean ± SD (%)	IC_50_ (µM), mean ± SD for sEH
**1a**	75.0 ± 5.3	\	85.7 ± 2.5	\
**1b**	55.6 ± 2.9		71.8 ± 4.7	\
**1c**	65.5 ± 3.7	\	47.7 ± 2.9	\
**1d**	No analysis, superimposition peaks	\	93.4 ± 0.6	\
**1e**	73.4 ± 5.2		86.9 ± 4.8	\
**1f**	47.6 ±4.4	\	36.1 ± 5.1	5.0 ± 0.9
**2a**	83.4 ± 4.0	\	74.3 ± 3.6	\
**2b**	17.6 ± 0.3	3.3 ± 0.5	21.7 ± 4.2	1.5 ± 0.2
**2c**	30.4 ± 1.9	4.0 ± 1.1	47.2 ± 2.5	6.3 ± 1.1
**2d**	21.5 ± 2.9	4.5 ± 1.3	41.6 ± 3.1	7.6 ± 1.0
MK886 (mPGES‐1 inhibitor)	33.5 ± 2.1	\	\	\
AUDA (sEH inhibitor)		\	6.1 ± 0.8	\

*Note*: Data are expressed as a percentage of control (100%) relative to the vehicle control (DMSO), *n* = 3. MK‐886 (10 μM) and AUDA (5 μM) were used as reference compounds for mPGES‐1 and sEH cell‐free assays, respectively.

Abbreviations: mPGES‐1, microsomal prostaglandin E2 synthase‐1; sEH, soluble epoxide hydrolase.

**Figure 2:**
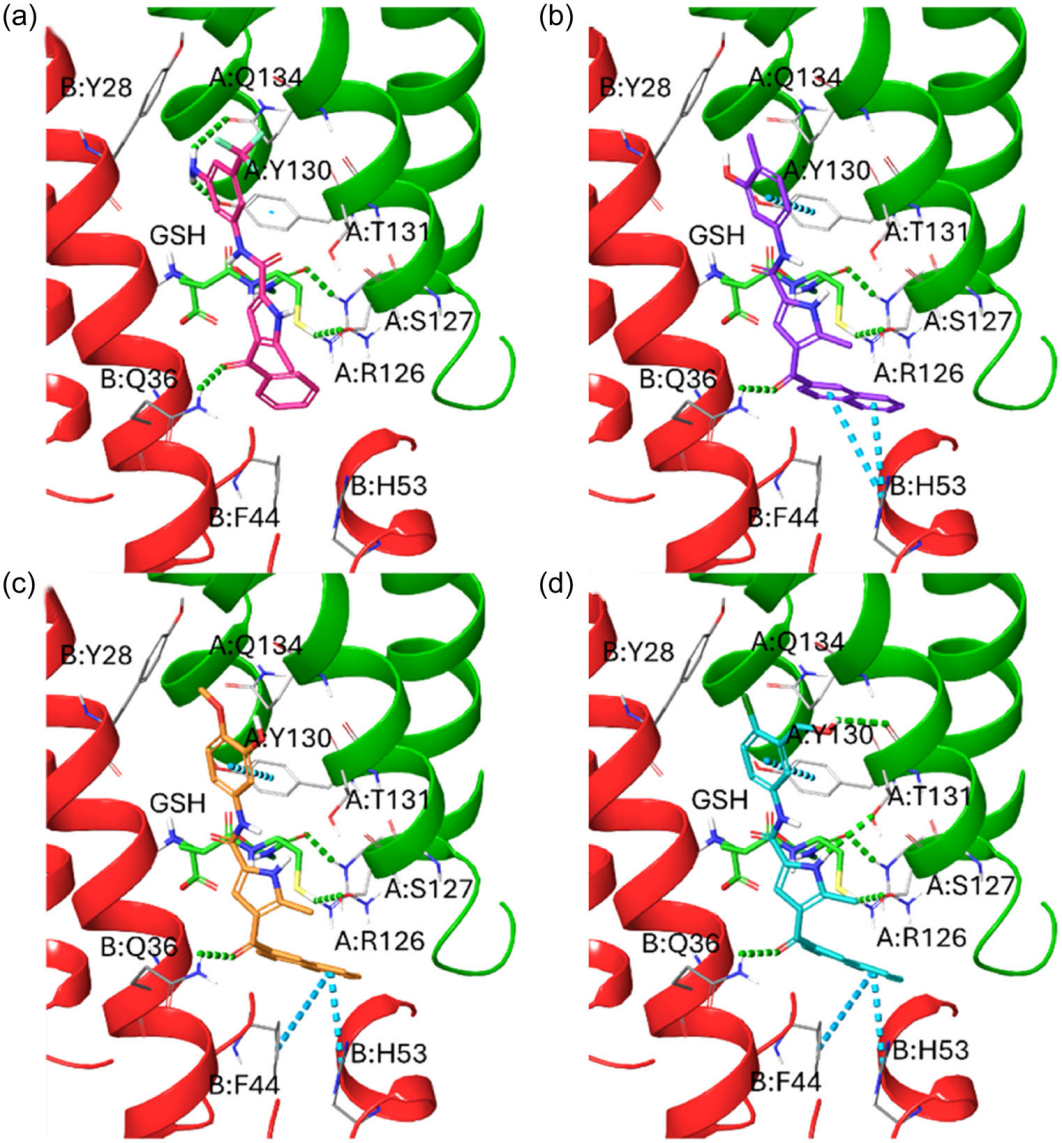
Three‐dimensional (3D) docking poses of (a) **1f** (colored by atom type: C fuchsia, O red, N blue, F light green, polar H white), (b) **2b** (colored by atom type: C violet, O red, N blue, polar H white), (c) **2c** (colored by atom type: C orange, O red, N blue, polar H white), (d) **2d** (colored by atom type: C sky blue, O red, N blue, Cl dark green, polar H white) in the mPGES‐1 binding site (chains A and B reported in green and red ribbons, respectively; GSH cofactor colored by atom type: C green, O red, N blue, S yellow, polar H white, residues colored by atom type: C gray, O red, N blue, polar H white). H‐bonds and π–π interactions are reported in green and cyan dotted lines, respectively.

In general, since most of the active compounds (**1 f**, **2b**–**2d**) selected from the virtual screening featured a naphthyl moiety at C‐4, we imputed this different behavior to the better accommodation of these molecules with the protein counterpart compared with the derivatives with the 4‐phenoyl group, as highlighted by molecular docking experiments. In detail, focusing on **2b**–**g**, the aromatic moieties at C‐4 were involved in π–π interactions (edge‐to‐face or face‐to‐face) with His53_chainB_ and Phe44_chainB_, as previously observed for the 5‐methyl‐4‐phenoyl‐1*H*‐pyrrole‐2‐carboxamide and 5‐methyl‐4‐naphtoyl‐1*H*‐pyrrole‐2‐carboxamide building blocks, whereas the second aromatic group on the amidic moiety interacted with Tyr130_chainA_, Thr131_chainA_, Gln134_chainA_, and Tyr28_chainB_. Interestingly, compound **1f**, which emerged as the only 4‐phenoyl‐based compound, showed a suboptimal edge‐to‐face π–π interaction with Phe44_chainB_, which, although partially offset by H‐bonds with Tyr130_chainA_ and Gln134_chainA_, may explain the slightly poorer mPGES‐1 inhibition than that observed for the 4‐naphtoyl‐based compounds **2b**–**2d** (Figure 2).

**Figure 3:**
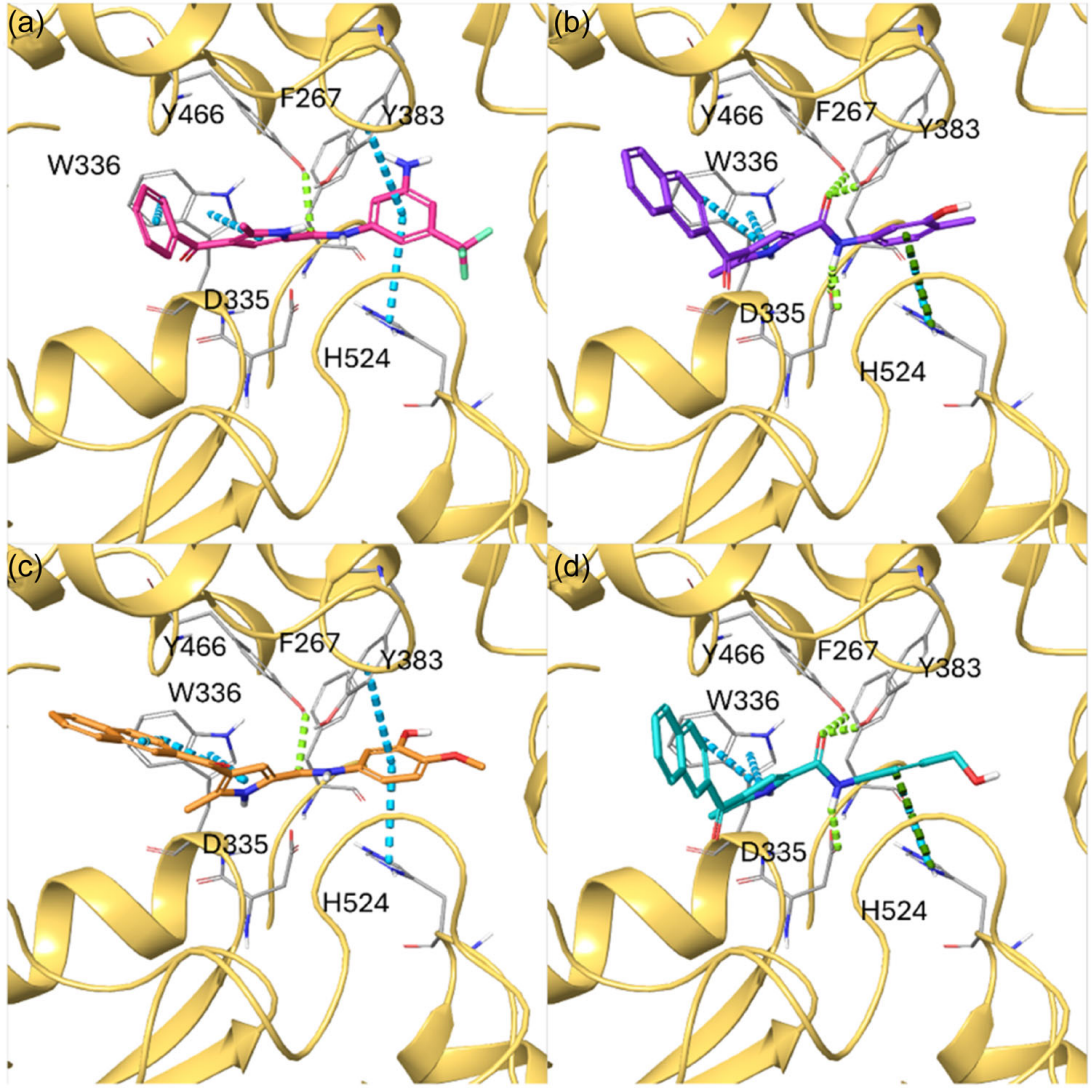
Three‐dimensional (3D) docking poses of (a) **1f** (colored by atom type: C fuchsia, O red, N blue, F light green, polar H white), (b) **2b** (colored by atom type: C violet, O red, N blue, polar H white), (c) **2c** (colored by atom type: C orange, O red, N blue, polar H white), and (d) **2d** (colored by atom type: C sky blue, O red, N blue, Cl dark green, polar H white) in the sEH binding site (chains reported in the dark yellow, residues colored by atom type: C gray, O red, N blue, polar H white). H‐bonds and π–π interactions are reported in green and cyan dotted lines, respectively.

Prompted by these encouraging data and supported by computational outcomes, we also investigated the synthesized compounds **1a**–**f** and **2a**–**d** against the sEH. In addition to mPGES‐1 interference, compounds **1f** and **2c**–**d** demonstrated a potent inhibitory effect against sEH, with IC_50_ values of 5.0 ± 0.9, 6.3 ± 1.1, and 7.6 ± 1.0 µM, respectively (Table 2). Interestingly, the most active compound in the series remained **2b**, exhibiting an IC_50_ value of 1.5 ± 0.2 µM. The other molecules in the series displayed similar behavior to that observed for mPGES‐1. Molecular docking calculations showed the ability of **1f** and **2c**–**d** to conveniently bind the sEH hydrolase binding site. In particular, the amide groups present in these compounds were able to make hydrogen bonds with the side chains of the key residues belonging to the catalytic triad, that is, Asp335, Tyr383, and Tyr466, similar to the urea‐based sEH reference binders^[^ ^53^, ^54^ ^]^ and also as recently reported by us for N‐(benzothazol‐2‐yl)amide‐based compounds.^[^ ^55^ ^]^ Furthermore, the two aromatic moieties at C‐2 and C‐4 established additional π–π interactions with Phe267, Trp336, and Tyr383 (Figure 3), enforcing their binding toward the hydrolase domain of sEH.

### 2.4 In vitro bioscreens for anticancer activity

**Figure 4:**
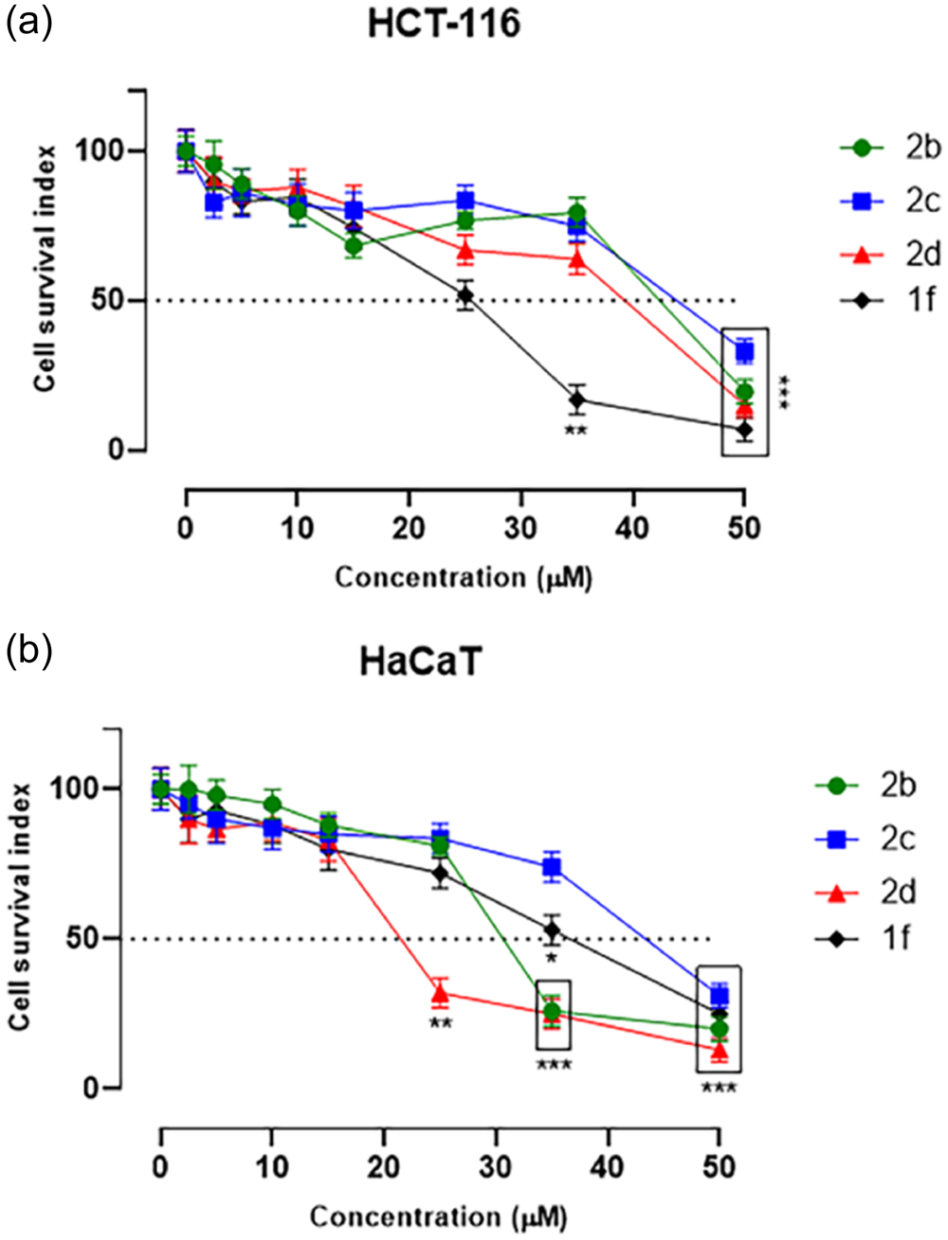
Cell survival index, evaluated by the MTT assay and live/dead cell ratio, for (a) HCT‐116 and (b) HaCaT cell lines following 48 h of incubation with a range of concentrations (0–50 µM) of **1f, 2b**, **2c**, and **2d** as indicated in the legend. Data are expressed as a percentage of untreated control cells and are reported as the mean of four independent experiments ± SEM (*n* = 24). The cell survival index was calculated as described in the experimental section and plotted in line graphs against the different concentrations of the tested molecules. *p ˂ 0.05 versus control cells; **p < 0.01 versus control cells; ***p < 0.001 versus control cells. HaCaT, high sensitivity of human epidermal keratinocytes; MTT, 3‐(4,5‐dimethyl‐2‐thiazolyl) 2,5‐diphenyl‐2‐H‐tetrazolium bromide.

mPGES‐1 has progressively become a well‐established target for cancer suppression as PGE_2_ can elicit a wide range of biological effects among others associated with neoplasm, particularly with CRC.^[^ ^56^, ^57^ ^]^ Based on their promising inhibitory effects on mPGES‐1, **1f, 2b**, **2c**, and **2d** were tested for potential antiproliferative effects on human HCT‐116 cells, a well‐established in vitro preclinical model of human CRC used in a variety of biomedical studies involving colon cancer proliferation.^[^ ^58^ ^]^ The results shown in Figure 4 as cell survival index highlight moderate antitumor effects for all the synthesized substances under investigation, for which IC_50_ values (Table 3) are observed in the low micromolar range (less than 50 µM). Interestingly, **1f** shows the most significant antiproliferative effects (IC_50_ = 25 µM), thus supporting the hypothesis that mPGES‐1 inhibition may be a useful tool for the control of specific tumor phenotypes. However, in the same experimental conditions, cellular responses obtained from the treatment of control healthy cultures (high sensitivity of human epidermal keratinocytes [HaCaT] cell line) demonstrate quite significant cytotoxic effects, especially at higher concentrations in actively proliferating cells (Table 3). On the other hand, these data on HCT‐116 and HaCaT cell lines highlighted a comparable behavior between the investigated compounds and cisplatin. Further efforts will be needed to make these potential drug candidates for future preclinical developments, endowed with an enhanced safety profile as well as selectivity toward cancer cells.

**Table 3:** IC_50_ values (µM) relative to **2b, 2c, 2d,** and **1f** in the HCT‐116 CRC cell line and HaCaT keratinocytes following 48 h of incubation.

IC_50_ ± SD (µM)		
Compound	HCT‐116	HaCat
**1f**	25 ± 2	37 ± 3
**2b**	42 ± 1	31 ± 4
**2c**	44 ± 3	43 ± 2
**2d**	39 ± 2	22 ± 2
Cisplatin	9 ± 3	20 ± 3

*Note*: IC_50_ values are calculated from concentration‐effect curves and reported as mean values ± SEM (*n* = 24).

## 3 CONCLUSIONS

In this work, we reported 5‐methyl‐2‐carboxamidepyrrole‐based compounds as new mPGES‐1/sEH inhibitors, highlighting their promising profile as anticancer agents. The combined application of virtual combinatorial screening, accessible chemical synthesis, and targeted biological investigations allowed the fast selection and oriented synthesis of a focused set of 5‐methyl‐2‐carboxamidepyrrole‐based derivatives. Following the synthetic route for obtaining *N*‐substituted derivatives, two virtual libraries of 4‐phenoyl‐ and 4‐naphtoyl‐based potentially synthesizable compounds were virtually built. Ten items were filtered from the molecular docking‐based virtual screening against mPGES‐1. Among them, three molecules (**2b**, **2c**, **2d**) emerged as dual‐target agents able to inhibit mPGES‐1 and sEH, another key enzyme involved in the ARA cascade and whose inhibition emerged as a promising strategy to disclose new anti‐inflammatory/anticancer agents, all endowed with promising activities in the low micromolar range for both targets. Also, compound **1f** showed a similar sEH inhibition profile (IC_50_ = 5.0 ± 0.9 µM) while also leading to mPGES‐1 residual activity of ~50%. Due to the involvement of mPGES‐1 and sEH in human CRC, **1f**, **2b**, **2c**, and **2d** were tested on the HCT‐116 CRC cell line for anticancer activity, disclosing significative antiproliferative effects, which highlight their potential as new candidate therapeutic agents. In line with the purposes of this study, we have identified a novel valuable core for the development of anticancer agents as dual inhibitors of mPGES‐1 and sEH, key targets in the ARA cascade. A deeper structure–activity relationship investigation will require the synthesis and biological evaluations of a larger set of compounds, which we have planned for the future.

## 4 EXPERIMENTAL

### 4.1 Computational chemistry

#### 4.1.1 Building of input ligands and the combinatorial libraries

The chemical structures of 5‐methyl‐4‐phenoyl‐1*H*‐pyrrole‐2‐carboxamide and 5‐methyl‐4‐naphtoyl‐1*H*‐pyrrole‐2‐carboxamide were built with Maestro Build Panel^[^ ^59^ ^]^ and processed with LigPrep,^[^ ^46^ ^]^ to generate all tautomers and protonation states at a pH of 7.4 ± 1.0, followed by an energy minimization step with the OPLS 2005 force field.

The input libraries of 5‐methyl‐4‐phenoyl‐1*H*‐pyrrole‐2‐carboxamide‐ (Library A) and 5‐methyl‐4‐naphtoyl‐1*H*‐pyrrole‐2‐carboxamide‐ (Library B) based compounds (See Figure 3 and Scheme 1) were obtained using CombiGlide software.^[^ ^45^ ^]^ This in silico tool allows the decoration of a starting molecule with a set of specific substituents according to the specific chemical synthons. In this case, 3097 amines, featuring at least one aromatic moiety and commercially available from Merck, were used for generating Library A (3097 items) and Library B (3097 items) to generate N‐substituted carboxamide derivatives.

The produced starting virtual library was processed with LigPrep^[^ ^46^ ^]^ to generate all the possible stereoisomers, tautomers, and protonation states at a pH of 7.4 ± 1.0, followed by a final energy minimization with the OPLS 2005 force field. The related pharmacokinetic properties were computed using QikProp software,^[^ ^47^, ^60^ ^]^ and “nondrug like” compounds were filtered out using the Reactive filter tool. Eventually, starting from Library A and Library B, two final libraries were obtained, comprising 1347 and 783 items, respectively.

#### 4.1.2 Protein preparation and virtual screening on mPGES‐1

mPGES‐1 3D model was prepared using the Schrödinger Protein Preparation Wizard,^[^ ^61^ ^]^ starting from the mPGES‐1 X‐ray structure in the active form co‐crystallized with LVJ (PDB code: 4BPM).^[^ ^62^ ^]^ The visual inspection of the protein structure disclosed the absence of structural water molecules assisting the binding of the reference co‐crystallized inhibitor (LVJ) and, accordingly, they were removed. Then, all hydrogen atoms were added, and bond orders were assigned.

For the subsequent molecular docking calculations, the co‐crystallized LVJ mPGES‐1 inhibitor was removed, and the GSH cofactor was left.

In detail, Library A and Library B were used as input for the molecular docking‐based Virtual Screening using Glide software^[^ ^48^ ^]^ and taking advantage of the Virtual Screening Workflow as implemented in the Schrödinger Suite, following the scheme:

− High‐Throughput Virtual Screening scoring and sampling (HTVS), saved the first 60% of ranked compounds and 1 pose for each compound;
− Standard Precision scoring and sampling phase (SP), saved the first 60% of ranked compounds and 1 pose for each compound;
− Extra Precision scoring and sampling phase (XP) saved the first 40% of ranked compounds and 20 maximum numbers of poses for each compound.

The selected compounds were then ranked according to the XP Glide Score. Also, the filtered items were visually inspected, evaluating the respect of the key interactions, leading to a focused set of molecules for the subsequent chemical synthesis (see Section 2).

5‐methyl‐4‐phenoyl‐1*H*‐pyrrole‐2‐carboxamide and 5‐methyl‐4‐naphtoyl‐1*H*‐pyrrole‐2‐carboxamide were docked against mPGES‐1 using the XP mode, setting a scaling factor of 0.8 related to van der Waals radii with a partial charge cutoff of 0.15. Based on a 0.5 kcal/mol rejection cutoff for the obtained minimized poses, a maximum of 20 poses was set in the post‐docking optimization phase.

### 4.2 Chemistry

The reagents and solvents were used as received from commercial suppliers without further purification unless otherwise indicated. All reactions involving air‐ or moisture‐sensitive reagents were performed under a nitrogen atmosphere using dried solvents and glassware. Reaction progress was monitored by analytical thin‐layer chromatography (TLC) on Merck 0.2 mm precoated silica (60 F254) aluminum sheets, with visualization by irradiation with a UV lamp (254 and 365 nm). ^1^H and ^13^C NMR spectra (see the Supporting Information) were recorded at 400 and 100 MHz, respectively, using a Bruker Avance 400 MHz spectrometer. Splitting patterns are described as singlet (s), doublet (d), triplet (t), quartet (q), quintuplet (qt), and broad (br). ESI‐HRMS spectra were acquired by a linear ion trap Orbitrap hybrid mass spectrometer (LTQ Orbitrap XL) (Thermo Fisher Scientific) operating in positive electrospray ionization mode. Data were collected and analyzed using the Xcalibur 2.2 software provided by the manufacturer. Chromatographic separations were performed on silica gel (Kieselgel 40, 0.040−0.063 mm, Merck). Yields refer to purified products and are not optimized. The purity of the samples was determined by the HPLC method, employing a Luna Phenomenex® C18 reversed‐phase column (250 × 10.0 mm, 5μ, 100 Å). The flow rate was maintained at 3 mL/min, and gradient elution was employed, starting from 90/10 of water/acetonitrile and gradually increasing to 100% of acetonitrile over a period of 40 min. Absorbance detection was performed at 240 nm. Compounds **5**, **6a**, **7a**–**b**, were obtained as previously reported.^[^ ^37^, ^63^ ^]^ Melting points were determined on a Stuart^TM^ SMP30 apparatus heating at 10°C/min up to the melting region.

The InChI codes of the investigated compounds, together with some biological activity data, are provided as Supporting Information.

#### 4.2.1 Synthesis of methyl 4‐(2‐naphthoyl)‐5‐methyl‐1*H*‐pyrrole‐2‐carboxylate (**8a**)

To a solution of Tos‐ΔAla(*N*‐Boc)‐OMe **5** (3.25 g, 9.14 mmol) in acetonitrile (50 mL) Cs_2_CO_3_ (3.57 g, 10.97 mmol) and 1‐(naphthalen‐2‐yl)butane‐1,3‐dione **6b** (2.33 g, 10.97 mmol) were added, and the resulting mixture was stirred at room temperature for 12 h. The solvent was removed under reduced pressure, and the residue was taken up in EtOAc (50 mL) and washed with saturated NH_4_Cl solution (2 × 45mL) and brine (2 × 45 mL). The organic phase was dried over anhydrous Na_2_SO_4_, filtered, and the solvent was removed under vacuum. A 20% TFA solution in DCM (40 mL) was added to the residue, and the mixture was stirred at room temperature overnight. Then, the mixture was washed with brine (2 x 30 mL), and 1M NaOH (2 x 30 mL). The organic phase was dried (Na_2_SO_4_), filtered, and concentrated under reduced pressure. The residue was purified by flash chromatography (SiO_2_, DCM/*n*‐hexane/EtOAc 7:2:1 v/v as an eluent) and recrystallized from Et_2_O*/n*‐hexane to give **8a** as a white solid Rf = 0.42. Yield: 53%; ^1^H NMR (CDCl_3_) *δ*: 2.68 (s, 3H), 3.87 (s, 3H), 7.17 (d, 1H, *J* = 2.6 Hz), 7.53–7.61 (m, 2H), 7.89–7.97 (m, 4H), 8.30 (s, 1H), 9.65 (brs, 1H); ^13^C‐DEPTq NMR (CDCl_3_) *δ*: 14.3, 52.1, 119.3, 120.5, 122.1, 125.7, 127.0, 128.1, 128.2, 128.5, 129.6, 130.5, 132.8, 135.3, 137.3, 141.1, 161.9, 192.1. (The spectroscopic data match with those reported in the literature).^[^ ^64^ ^]^

#### 4.2.2 Synthesis of methyl 4‐acetyl‐5‐(naphthalen‐2‐yl)‐1*H*‐pyrrole‐2‐carboxylate (**8b**)

Compound **8b** was isolated as a minor isomer from the procedure reported for **8a**. Rf = 0.72. Pale yellow solid. Yield: 14%; ^1^H NMR (CDCl_3_) *δ*: 2.40 (s, 3H), 3.83 (s, 3H), 7.38 (d, 1H, *J *= 2.7 Hz), 7.50–7.56 (m, 2H), 7.66 (dd, 1H, *J *= 8.5, 1.8 Hz), 7.86–7.90 (m, 3H), 8.05 (s, 1H), 9.56 (brs, 1H); ^13^C‐DEPTq NMR (CDCl_3_) *δ*: 29.3, 52.2, 118.4, 122.4, 123.6, 127.0 (2 C), 127.4, 128.1, 128.4, 128.7 (2 C), 128.9, 133.3, 133.9, 140.1, 161.6, 194.1 (see also 2D‐ROESY NMR spectrum in Supporting Information).

#### 4.2.3 Synthesis of 4‐benzoyl‐5‐methyl‐1*H*‐pyrrole‐2‐carboxylic acid (**9a**)

To a solution of **7a** (1.20 g, 4.93 mmol) in MeOH (25 mL) 2 M NaOH (12.3 mL, 24.60 mmol) was added, and the resulting mixture was heated at reflux until not starting material was detected by TLC (EtOAc/*n*‐hexane 3:7) (~2 h). The organic solvent was removed under reduced pressure, then 6 N HCl was added (pH ~ 3), and the mixture was extracted with EtOAc (3 × 50 mL). The combined organic extracts were dried over anhydrous Na_2_SO_4_, filtered, and the solvent was removed under reduced pressure to give **9a** as a white solid. Yield: 96%. ^1^H NMR (CDCl_3_) *δ*: 2.57 (s, 3H), 6.75 (d, 1H, *J *= 2.6 Hz), 7.42–7.46 (m, 2H), 7.49–7.53 (m, 1H), 7.77 (d, 2H, *J* = 6.9 Hz), 11.39 (brs, 1H); ^13^C‐DEPTq NMR (CDCl_3_) *δ*: 13.8, 116.8, 121.3, 122.9, 128.5, 129.2, 131.7, 139.9, 140.6, 162.8, 192.5.

#### 4.2.4 Synthesis of 4‐(2‐naphthoyl)‐5‐methyl‐1*H*‐pyrrole‐2‐carboxylic acid (**9b**)

Starting from **8a** (2.5 g, 8.52 mmol) and following the same procedure reported for **9a,** the title compound was obtained as a white solid. Yield: 93%; ^1^H NMR (DMSO‐*d*_6_) *δ*: 2.52 (s, 3H, overlapped), 6.93 (d, 1H, *J* = 2.4 Hz), 7.59–7.68 (m, 2H), 7.80 (dd, 1H, *J *= 8.5, 1.7 Hz), 8.00–8.06 (m, 2H), 8.12 (d, 1H, *J *= 8.0 Hz), 8.30 (s, 1H), 12.41 (brs, 1H); ^13^C‐DEPTq NMR (DMSO‐*d*_6_) *δ*: 14.2, 118.8, 121.3, 122.1, 126.0, 127.7, 128.6, 128.8, 128.9, 130.1 (2 C), 132.9, 135.2, 138.0, 141.5, 162.4, 191.6.

#### 4.2.5 Synthesis of 4‐benzoyl‐5‐methyl‐*N*‐(quinolin‐2‐yl)‐1*H*‐pyrrole‐2‐carboxamide (**1b**) and 4‐benzoyl‐*N*‐(3,5‐dichlorophenyl)‐5‐methyl‐1*H*‐pyrrole‐2‐carboxamide (1e)

To a solution of **9a** (0.150 g, 0.65 mmol) in dry toluene, (1 mL) oxalyl chloride (0.165 g, 1.30 mmol) and two drops of *N*,*N*‐dimethylformamide (DMF) were added, and the resulting mixture was heated at 50°C for 2 h. The solvent was removed under reduced pressure, and the residue was dissolved in dry DCM (1 mL), then 2‐aminoquinoline (0.187 g, 1.30 mmol, for **1b**) or 3,5‐dichloroaniline (0.211 g, 1.30 mmol, for **1e**) and dry triethylamine (0.132 g, 1.30 mmol) were added, and the mixture was stirred overnight at room temperature. The solvent was removed under reduced pressure, and the residue was taken up in EtOAc (25 mL) and washed with 1 M NaOH (2 × 20 mL) and brine (2 × 25 mL). The organic phase was dried over anhydrous Na_2_SO_4_, filtered, and the solvent was removed under *vacuo*. The crude was purified by flash chromatography (SiO_2_, *n*‐hexane/EtOAc 1:1 as eluent) and recrystallized (*n*‐hexane/EtOAc) to give the title compounds. (**1b**): white solid. Yield: 68%; mp: 194–195°C; ^1^H NMR (DMSO‐*d*_6_) *δ*: 2.56 (s, 3H), 7.50 (t, 1H, *J *= 7.5 Hz), 7.55–7.59 (m, 2H), 7.61–7.66 (m, 1H), 7.69–7.74 (m, 2H), 7.79–7.85 (m, 3H), 7.93 (d, 1H, *J *= 8.1 Hz), 8.38 (m, 2H), 10.98 (s, 1H), 12.43 (brs, 1H); ^13^C NMR (DMSO‐*d*_6_) *δ*: 14.3, 116.1, 117.6, 121.0, 124.4, 125.8, 126.5, 127.7, 128.7, 129.2, 129.6, 130.8, 132.2, 138.9, 140.7, 141.8, 147.4, 152.9, 160.4, 191.6. HRMS (ESI) *m/z* [M+H]^+^ calcd. for [C_22_H_18_N_3_O_2_]^+^ 356.1393, found, 356.1388. (**1e**): Pale yellow solid. Yield: 72%; mp: 272–273°C; ^1^H NMR (DMSO‐*d*_6_) δ: 2.51 (s, 3H, overlapped), 7.21 (t, 1H, *J *= 1.9 Hz), 7.36 (s, 1H), 7.52–7.56 (m, 2H), 7.59–7.63 (m, 1H), 7.72 (d, 2H, *J* = 6.8 Hz), 7.80 (d, 2H, *J* = 1.9 Hz), 10.09 (s, 1H), 12.38 (s, 1H); ^13^C‐DEPTq NMR (DMSO‐*d*_6_) δ: 14.5, 116.6, 118.8, 121.3, 123.4, 124.6, 129.6, 129.7, 132.7, 135.2, 141.1, 142.0, 142.7, 160.1, 192.2. HRMS (ESI) *m/z* [M+H]^+^ calcd. for [C_19_H_15_Cl_2_N_2_O_2_]^+^ 373.0505, found, 373.0495; *m/z* [M+Na]^+^ calcd. for [C_19_H_14_Cl_2_N_2_O_2_Na]^+^ 395.0324, found 395.0314.

#### 4.2.6 General procedure for the synthesis of compounds **1a**, **1c**, **1 d**, **1 f**, and **2a–d**

To a solution of acid **9a** or **9b** (0.50 mmol, 1 eq) in DMF (5 mL), HOBT (2.5 eq), HBTU (2.5 eq), and *N*‐methylmorfoline (NMM) (5 eq) were added, and the resulting mixture was stirred at 0°C for 30 min. Then, appropriate amine (2.5 eq) was added, and the mixture was allowed to room temperature and stirred in these conditions for 12 h. The solvent was removed under reduced pressure, and the residue was taken up in EtOAc (50 mL) and washed with 2 M KHSO_4_ (2 × 35 mL), saturated NaHCO_3_ solution (2 × 35 mL) and saturated NaCl solution (2 × 35 mL). The organic phase was dried over anhydrous Na_2_SO_4_, filtered, and the solvent was removed under vacuum. The crude was purified by flash chromatography (SiO_2_, DCM/*n*‐hexane/EtOAc 1:6:3 as eluent) and recrystallized (*n*‐hexane/EtOAc) to give the title compounds **1a**, **1c**, **1d**, **1f,** and **2a**–**d** in good yields.

4‐Benzoyl‐5‐methyl‐*N*‐[3‐(trifluoromethyl)benzyl+]‐1*H*‐pyrrole‐2‐carboxamide (**1a**): White solid. Yield: 63%; mp: 235–236°C; ^1^H NMR (DMSO‐*d*_6_) δ: 2.49 (s, 3H), 4.50 (d, 2H, *J* = 6.0 Hz), 7.12 (d, 1H, *J* = 2.5 Hz), 7.50–7.54 (m, 2H), 7.57–7.65 (m, 5H), 7.71 (d, 2H, *J* = 8.1 Hz), 8.80 (t, 1H, *J* = 6.1 Hz), 12.21 (brs, 1H); ^13^C NMR (DMSO‐*d*_6_) *δ*: 14.2, 42.5, 114.5, 120.6, 124.5 (q, *J*_C‐F_ = 3.9 Hz), 124.8 (q, *J*_C‐F_ = 3.9 Hz), 124.9, 125.2 (d, *J*_C‐F_ = 271.0 Hz), 129.2, 129.3, 129.9 (d, *J*_C‐F_ = 31.3 Hz), 130.3, 132.1, 132.4, 140.2, 141.1, 142.2, 161.3, 191.8. HRMS (ESI) *m/z* [M+H]^+^ calcd. for [C_21_H_18_F_3_N_2_O_2_]^+^ 387.1315, found, 387.1311; *m/z* [M+Na]^+^ calcd. for [C_21_H_17_F_3_N_2_O_2_Na]^+^ 409.1134, found 409.1130.

4‐Benzoyl‐*N*‐(3,4‐dimethylphenyl)‐5‐methyl‐1*H*‐pyrrole‐2‐carboxamide (**1c**): White solid. Yield: 76%; mp: 224–225°C; ^1^H NMR (DMSO‐*d*_6_) *δ*: 2.16 (s, 3H), 2.18 (s, 3H), 2.49 (s, 3H), 7.07 (d, 1H, *J *= 8.1 Hz), 7.32 (s, 1H), 7.37–7.39 (m, 2H), 7.53–7.57 (m, 2H), 7.61–7.64 (m, 1H), 7.73 (d, 2H, *J *= 8.1 Hz), 9.78 (s, 1H), 12.14 (s, 1H); ^13^C NMR (DMSO‐*d*_6_) *δ*: 14.8, 20.3, 21.1, 116.1, 119.4, 121.6, 123.1, 125.9, 130.1, 130.2, 131.2, 133.2, 133.3, 137.8, 138.1, 141.3, 141.9, 160.4, 193.4. HRMS (ESI) *m/z* [M+H]^+^ calcd. for [C_21_H_21_N_2_O_2_]^+^ 333.1597, found, 333.1592; *m/z* [M+Na]^+^ calcd. for [C_21_H_20_N_2_O_2_Na]^+^ 355.1417, found 355.1411.

(±) 4‐Benzoyl‐*N*‐[4‐(1‐hydroxyethyl)phenyl]‐5‐methyl‐1*H*‐pyrrole‐2‐carboxamide (**1d**): White solid. Yield: 75%; mp: 203–204°C; ^1^H NMR (DMSO‐*d*_6_) *δ*: 1.31 (d, 3H, *J* = 6.4 Hz), 2.51 (s, 3H, overlapped), 4.65–4.71 (m, 1H), 5.06 (d, 1H, *J* = 4.2 Hz), 7.28 (d, 2H, *J* = 8.6 Hz), 7.36 (d, 1H, *J* = 2.3 Hz), 7.54–7.58 (m, 2H), 7.60–7.65 (m, 3H), 7.74 (d, 2H, *J* = 6.8 Hz), 9.83 (s, 1H), 12.30 (brs, 1H); ^13^C NMR (DMSO‐*d*_6_) *δ*: 14.2, 26.8, 68.6, 115.2, 120.6, 120.8, 125.1, 126.4, 129.2, 129.4, 132.2, 138.4, 140.8, 141.1, 143.1, 159.6, 191.9. HRMS (ESI) *m/z* [M+H]^+^ calcd. for [C_21_H_21_N_2_O_3_]^+^ 349.1547, found 349.1542; *m/z* [M+Na]^+^ calcd. for [C_21_H_20_N_2_O_3_Na]^+^ 371.1366, found 371.1362.

N‐[3‐Amino‐5‐(trifluoromethyl)phenyl]‐4‐benzoyl‐5‐methyl‐1*H*‐pyrrole‐2‐carboxamide (**1f**): White solid. Yield: 60%; mp: 223–225°C; ^1^H NMR (DMSO‐*d*_6_) *δ*: 2.52 (s, 3H, overlapped), 5.61 (bs, 2H), 6.55 (brt, 1H), 7.17 (brt, 1H), 7.29 (brt, 1H), 7.39 (d, 1H, *J *= 4.0 Hz), 7.54–7.58 (m, 2H), 7.60–7.65 (m, 1H), 7.74 (d, 2H, *J *= 8.0 Hz), 9.81 (brs, 1H), 12.30 (brs, 1H); ^13^C‐DEPTq NMR (DMSO‐*d*_6_) *δ*: 14.2, 104.4 (d, *J*_CF _= 4.0 Hz), 105.6 (d, *J*_CF_ = 3.0 Hz), 108.7, 115.7, 120.8, 124.9, 125.4 (d, *J*_CF_ = 270.0 Hz), 129.3, 129.5, 130.7 (q, *J*_CF _= 31.3 Hz), 132.3, 141.0, 141.1, 141.4, 150.8, 159.7, 191.9. HRMS (ESI) *m/z* [M+H]^+^ calcd. for [C_20_H_17_F_3_N_3_O_2_]^+^ 388.1267; found 388.1262.

4‐(2‐Naphthoyl)‐*N*‐(2‐ethylphenyl)‐5‐methyl‐1*H*‐pyrrole‐2‐carboxamide (**2a**): White solid. Yield: 93%; mp: 246–247°C; ^1^H NMR (DMSO‐*d*_6_) *δ*: 1.11 (t, 3H, *J* = 7.5 Hz), 2.55 (s, 3H, overlapped), 2.59 (q, 2H, *J* = 7.5 Hz, overlapped), 7.18–7.29 (m, 4H), 7.41 (brs, 1H), 7.60–7.67 (m, 2H), 7.84 (dd, 1H, *J* = 8.5, 1.7 Hz), 8.02 (d, 1H, *J *= 7.7 Hz), 8.06 (d, 1H, *J *= 8.5 Hz), 8.12 (d, 1H, *J *= 7.7 Hz), 8.36 (s, 1H), 9.57 (s, 1H), 12.34 (s, 1H); ^13^C‐DEPTq NMR (DMSO‐*d*_6_) *δ*: 14.2, 15.0, 24.9, 115.3, 121.0, 125.1, 126.0, 126.9, 127.2, 127.6, 128.4, 128.5, 128.7, 128.8, 129.4, 130.2 (2 C), 132.9, 135.1, 136.2, 138.2, 140.5, 140.7, 160.2, 191.8. HRMS (ESI) *m/z* [M+H]^+^ calcd. for [C_25_H_23_N_2_O_2_]^+^ 383.1754; found, 383.1746; *m/z* calcd.[M+Na]^+^ for [C_25_H_22_N_2_O_2_Na]^+^ 405.1573, found 405.1564.

4‐(2‐Naphthoyl)‐*N*‐(3‐hydroxy‐4‐methylphenyl)‐5‐methyl‐1*H*‐pyrrole‐2‐carboxamide (**2b**): White solid. Yield: 65%; mp: 273–274°C; ^1^H NMR (DMSO‐*d*_6_) *δ*: 2.06 (s, 3H), 2.55 (s, 3H), 6.96 (m, 2H), 7.29 (s, 1H), 7.44 (d, 1H, *J *= 2.5 Hz), 7.60–7.69 (m, 2H), 7.83 (dd, 1H, *J *= 8.4, 1.7 Hz), 8.03 (d, 1H, *J* = 8.0 Hz), 8.07 (d, 1H, *J* = 8.5 Hz), 8.14 (d, 1H, *J* = 7.9 Hz), 8.34 (s, 1H), 9.28 (s, 1H), 9.65 (s, 1H), 12.29 (s, 1H); ^13^C‐DEPTq NMR (DMSO‐*d*_6_) *δ*: 14.2, 16.4, 107.8, 111.7, 115.4, 119.7, 121.0, 125.3, 126.1, 127.6, 128.5, 128.7, 128.9, 130.2 (2 C), 131.0, 133.0, 135.1, 138.3, 138.4, 140.6, 156.0, 159.4, 191.9. HRMS (ESI) *m/z* [M+H]^+^ calcd. for [C_24_H_21_N_2_O_3_]^+^ 385.1547, found 385.1539; *m/z* [M+Na]^+^ calcd. for [C_24_H_20_N_2_O_3_Na]^+^ 407.1366, found 407.1356.

4‐(2‐Naphthoyl)‐*N*‐(3‐hydroxy‐4‐methoxyphenyl)‐5‐methyl‐1*H*‐pyrrole‐2‐carboxamide (**2c**): White solid. Yield: 55%; mp: 208–209°C; ^1^H NMR (DMSO‐*d*_6_) *δ*: 2.54 (s, 3H), 3.73 (s, 3H), 6.85 (d, 1H, *J *= 8.7 Hz), 7.04 (dd, 1H, *J* = 8.7, 2.5 Hz), 7.25 (d, 1H, *J* = 2.2 Hz), 7.41 (d, 1H, *J* = 2.5 Hz), 7.60–7.69 (m, 2H), 7.82 (dd, 1H, *J *= 8.5, 1.7 Hz), 8.03 (d, 1H, *J *= 7.8 Hz), 8.07 (d, 1H, *J *= 8.5 Hz), 8.14 (d, 1H, 7.9 Hz), 8.33 (s, 1H), 9.00 (s, 1H), 9.61 (s, 1H), 12.29 (s, 1H); ^13^C‐DEPTq NMR (DMSO‐*d*_6_) *δ*: 14.2, 56.8, 109.5, 111.7, 113.3, 115.2, 121.0, 125.3, 126.1, 127.6, 128.5, 128.7, 128.9, 130.1, 130.2, 133.0, 133.5, 135.1, 138.3, 140.5, 144.7, 147.2, 159.3, 191.9. HRMS (ESI) *m/z* [M+H]^+^ calcd. for [C_24_H_21_N_2_O_4_]^+^ 401.1496, found 401.1491; *m/z* [M+Na]^+^ calcd. for [C_24_H_20_N_2_O_4_Na]^+^ 423.1315, found 423.1310.

4‐(2‐Naphthoyl)‐*N*‐[4‐chloro‐3‐(hydroxymethyl)phenyl]‐5‐methyl‐1*H*‐pyrrole‐2‐carboxamide (**2 d**): White solid. Yield: 88%; mp: 260‐261°C; ^1^H NMR (DMSO‐*d*_6_) *δ*: 2.54 (s, 3H), 4.52 (d, 2H, *J *= 5.5 Hz), 5.41 (t, 1H, *J* = 5.5 Hz), 7.32 (d, 1H, *J *= 8.5 Hz), 7.50 (d, 1H, *J *=– 2.3 Hz), 7.60–7.68 (m, 2H), 7.77–7.84 (m, 3H), 8.03 (d, 1H, *J* = 7.8 Hz), 8.07 (d, 1H, *J* = 8.5 Hz), 8.13 (d, 1H, *J *= 7.8 Hz), 8.33 (s, 1H), 9.95 (s, 1H), 12.35 (s, 1H); ^13^C‐DEPTq NMR (DMSO‐*d*_6_) *δ*: 14.2, 61.1, 115.9, 120.1, 120.2, 121.2, 124.9, 125.3, 126.0, 127.6, 128.5, 128.8, 128.9, 129.7, 130.2 (2 C), 133.0, 135.1, 138.2, 139.1, 140.7, 141.0, 159.6, 191.9. HRMS (ESI) *m/z* [M+H]^+^ calcd. for [C_24_H_20_ClN_2_O_3_]^+^ 419.1157, found 419.1148; *m/z* [M+Na]^+^ calcd. for [C_24_H_19_ClN_2_O_3_Na]^+^ 441.0976, found 441.0966.

### 4.3 Biophysical assays

#### 4.3.1 Cell‐free mPGES‐1 activity assay

Microsomes of A549 cells containing mPGES‐1 were used as enzyme sources. In brief, A549 cells were stimulated with interleukin‐1β (IL‐1β) (2 ng/mL) for 48 h, harvested, and sonicated. The lysate was first centrifugated at 10,000*g* (10 min), and then at 174,000*g* for (1 h) at 4°C. The pellet (microsomal fraction) was resuspended in 1 ml homogenization buffer (0.1 M potassium phosphate buffer, pH = 7.4, 1 mM phenylmethanesulfonyl fluoride, 60 µg/mL soybean trypsin inhibitor, 1 µg/mL leupeptin, 2.5 mM GSH, and 250 mM sucrose), the total protein concentration was determined, and microsomes were diluted in potassium phosphate buffer (0.1 M, pH 7.4) containing GSH (2.5 mM) and seeded in a 96‐well plate. Compounds **1a**–**1f** and **2a**–**2d** or DMSO (1%) were preincubated with the microsomes for 15 min on ice, and the enzymatic production of PGE_2_ was triggered by adding 20 µM of PGH_2._ After 1 min, the addition of 100 µL of a stop solution (40 mM FeCl_3_, 80 mM citric acid, and 10 µM 11β‐PGE_2_) blocked the reaction. PGE_2_ and 11β‐PGE_2_ were extracted by solid‐phase extraction, and PGE_2_ formation was quantified by RP‐HPLC.^[^ ^52^ ^]^

#### 4.3.2 Cell‐free sEH activity assay

All compounds were tested in a single‐dose triplicate mode at a concentration of 10 μM against sEH using a Soluble Epoxide Hydrolase Inhibitor Screening Assay Kit (10011671).^[^ ^65^ ^]^ For compounds **2b**–**d** and **1f,** the IC_50_ value was calculated using diluted threefold concentrations, from 30,000 to 1.52 nM. Briefly, isolated human recombinant sEH was diluted in bis‐Tris buffer (25 mM, pH 7) and preincubated with test compounds or the reference compound (AUDA) or vehicle (2.5% DMSO) for 15 min at 25°C. The reaction was started by the addition of 5 μL of substrate (PHOME, 10 μM in DMSO) to obtain a final concentration of 0.25 μM). When the epoxide moiety of PHOME is hydrolyzed by the epoxide hydrolase, an intramolecular cyclization occurs, which results in the release of a cyanohydrin under basic conditions. The cyanohydrin quickly decomposes into cyanide ion and the highly fluorescent 6‐Methoxy‐2‐naphthaldehyde, which can be analyzed. The signal was detected (λex 330 nm, λem 465 nm) on an EnSpire™ Multimode Plate Reader (PerkinElmer).

### 4.4 Preclinical studies in cellular models

#### 4.4.1 Human cell cultures

HCT‐116 cells (CCL‐247 ™), an epithelial‐like carcinoma cell line isolated from the colon of an adult male with colon cancer, were purchased from ATCC and cultured in Roswell Park Memorial Institute (Invitrogen) supplemented with 10% fetal bovine serum (FBS, Cambrex), l‐glutamine (2 mM, Sigma), penicillin (100 units/mL, Sigma), and streptomycin (100 μg/mL, Sigma), and cultured in a humidified 5% carbon dioxide atmosphere at 37°C, according to ATCC recommendations.^[^ ^58^ ^]^

HaCaT cells, human immortalized keratinocytes (kindly provided by Dr. Valeria Cicatiello at Italian National Research Council (CNR), Institute of Genetics and Biophysics), were maintained in a humidified 5% CO2 atmosphere at 37°C and grown in DMEM (Invitrogen) supplemented with 10% fetal bovine serum (FBS, Cambrex), l‐glutamine (2 mM), penicillin (100 units/mL, Sigma‐Aldrich), and streptomycin (100 μg/mL). Cells were seeded at a density between 2 and 4 × 104 cells/cm^2^ and cultured to about 80%–90% confluence.^[^ ^66^ ^]^ HaCaT cells provide ideal in vitro cell systems to study biocompatibility and toxicological cellular responses.

#### 4.4.2 *In vitro bioscreens *for anticancer activity

Bioactivity and cell responses to in vitro treatment with **1f**, **2b**, **2c**, and **2d** compounds were investigated through the estimation of a “cell survival index,” arising from the combination of cell viability evaluation with cell counting. The cell survival index is calculated as the arithmetic mean between the percentage values derived from the 3‐(4,5‐dimethyl‐2‐thiazolyl) 2,5‐diphenyl‐2‐H‐tetrazolium bromide (MTT) assay and the automated cell count.^[^ ^67^ ^]^ HCT‐116 and HaCaT were inoculated in 96‐microwell culture plates at a density of 10^4^ cells/well and allowed to grow for 24 h. Subsequently, the culture medium was exchanged with fresh medium, and cells were exposed to different concentrations (0→50 µM) of compounds **1f**, **2b**−**2d** under investigation for an additional 48 h. After the treatments, the medium was removed, and the cells were incubated with 20 μL/well of an MTT solution (5 mg/mL MTT, Sigma) for 1 h in a humidified 5% CO_2_ incubator at 37°C. The incubation was stopped by removing the MTT solution and by adding 100 μL/well of DMSO to solubilize the obtained formazan. Finally, the absorbance was monitored at 550 nm using a microplate reader (iMark microplate reader, Bio‐Rad). Cell number was determined by TC20 automated cell counter (Bio‐Rad), which uses disposable slides, TC20 trypan blue dye (0.4% trypan blue dye w/v in 0.81% sodium chloride and 0.06% potassium phosphate dibasic solution), and a CCD camera to count cells based on the analyses of captured images. The medium was removed, and the cells were collected. Ten microliters of cell suspension, mixed with 0.4% trypan blue solution at a 1:1 ratio, were loaded into the chambers of disposable slides. The results are expressed in terms of total cell count (cell number/mL). If the presence of try‐pan blue is identified, the instrument incorporates the dilution factor and displays both the count of live cells and the percentage of viability. Total counts and live/dead ratio from random samples for each cell line were subjected to comparisons with manual hemocytometers in control experiments. The determination of the IC_50_ values relies on plots of data (*n* = 6 for each experiment), repeated four times for a total of 24 samples (*n* = 24). Concentration‐effect curves were obtained with nonlinear regression using GraphPad Prism 8.0 curve‐fitting software.^[^ ^67^ ^]^

## CONFLICTS OF INTEREST STATEMENT

The authors declare no conflicts of interest.

## Supporting information

Supporting information.

Supporting information.

## Data Availability

The data are available on request from the authors.
