# Supplementary material for: 5‐methyl‐2‐carboxamidepyrrole‐based novel dual mPGES‐1/sEH inhibitors as promising anticancer candidates
Source: Arch Pharm (Weinheim). 2024 Dec 18;358(1):e2400708. doi: 10.1002/ardp.202400708 (PMC11653428; doi:10.1002/ardp.202400708)
Supplement: Supplementary file 1 — Supporting information. [file ARDP-358-e2400708-s002.doc]

**Supplemental Material: Novel Compounds and Biological Screening Results**

5-Methyl-2-carboxamidepyrrole-based novel dual mPGES-1/sEH inhibitors as promising anticancer candidates

Ester Colarusso^1,ǁ^, Gianluigi Lauro^1,ǁ^, Marianna Potenza^1,2^, Paola Galatello^1^, Maria Luisa d'Aulisio Garigliota^1^, Maria Grazia Ferraro^3^, Marialuisa Piccolo^4^, Maria Giovanna Chini^5^, Carlo Irace^4^, Pietro Campiglia^1^, Robert Klaus Hoffstetter^2^, Oliver Werz^2^, Anna Ramunno^1^,*, Giuseppe Bifulco^1^,*

1 Department of Pharmacy, University of Salerno, Via Giovanni Paolo II 132, Fisciano 84084, Italy

2 Department of Pharmaceutical/Medicinal Chemistry, Institute of Pharmacy, Friedrich Schiller University, Philosophenweg 14, Jena, 07743, Germany

3 Department of Molecular Medicine and Medical Biotechnologies, School of Medicine and Surgery, University of Naples, Via Domenico Montesano 49, Naples 80131, Italy

4 BioChem Lab, Department of Pharmacy, School of Medicine and Surgery, University of Naples, Via Domenico Montesano 49, Naples 80131, Italy

5 Department of Biosciences and Territory, University of Molise, C.da Fonte Lappone, Pesche 86090, Italy

- Dr. Anna Ramunno, Department of Pharmacy, University of Salerno, Via Giovanni Paolo II 132, Fisciano 84084, Italy (Email: [aramunno@unisa.it](mailto:aramunno@unisa.it))
- Prof. Giuseppe Bifulco, Department of Pharmacy, University of Salerno, Via Giovanni Paolo II 132, Fisciano 84084, Italy (Email: bifulco@unisa.it)

| **Compound No.** | **InChI** | **Biological Activity (*K*_i_, IC_50_, EC_50_, % activity etc.)^a^** |
| --- | --- | --- |
| 1a | InChI=1S/C21H17F3N2O2/c1-13-17(19(27)15-7-3-2-4-8-15)11-18(26-13)20(28)25-12-14-6-5-9-16(10-14)21(22,23)24/h2-11,26H,12H2,1H3,(H,25,28) | - mPGES-1 residual activity: 75.0 ± 5.3 % - sEH residual activity: 85.7 ± 2.5 % |
| 1b | InChI=1S/C22H17N3O2/c1-14-17(21(26)16-8-3-2-4-9-16)13-19(23-14)22(27)25-20-12-11-15-7-5-6-10-18(15)24-20/h2-13,23H,1H3,(H,24,25,27) | - mPGES-1 residual activity: 55.6 ± 2.9 % - sEH residual activity: 71.8 ± 4.7 % |
| 1c | InChI=1S/C21H20N2O2/c1-13-9-10-17(11-14(13)2)23-21(25)19-12-18(15(3)22-19)20(24)16-7-5-4-6-8-16/h4-12,22H,1-3H3,(H,23,25) | - mPGES-1 residual activity: 65.5 ± 3.7 % - sEH residual activity: 47.7 ± 2.9 % |
| 1d | InChI=1S/C21H20N2O3/c1-13-18(20(25)16-6-4-3-5-7-16)12-19(22-13)21(26)23-17-10-8-15(9-11-17)14(2)24/h3-12,14,22,24H,1-2H3,(H,23,26) | - mPGES-1 residual activity: incalculable - sEH residual activity: 93.4 ± 0.6 % |
| 1e | InChI=1S/C19H14Cl2N2O2/c1-11-16(18(24)12-5-3-2-4-6-12)10-17(22-11)19(25)23-15-8-13(20)7-14(21)9-15/h2-10,22H,1H3,(H,23,25) | - mPGES-1 residual activity: 73.4 ± 5.2 % - sEH residual activity: 86.9 ± 4.8 % |
| 1f | InChI=1S/C20H16F3N3O2/c1-11-16(18(27)12-5-3-2-4-6-12)10-17(25-11)19(28)26-15-8-13(20(21,22)23)7-14(24)9-15/h2-10,25H,24H2,1H3,(H,26,28) | - mPGES-1 residual activity: 47.6 ±4.4 % - sEH IC_50_: 5.0 ± 0.9 µM - HTC-116 IC_50_: 25 ± 2 µM - HaCat IC_50_: 37 ± 3 µM |
| 2a | InChI=1S/C25H22N2O2/c1-3-17-8-6-7-11-22(17)27-25(29)23-15-21(16(2)26-23)24(28)20-13-12-18-9-4-5-10-19(18)14-20/h4-15,26H,3H2,1-2H3,(H,27,29) | - mPGES-1 residual activity: 83.4 ± 4.0 % - sEH residual activity: 74.3 ± 3.6 % |
| 2b | InChI=1S/C24H20N2O3/c1-14-7-10-19(12-22(14)27)26-24(29)21-13-20(15(2)25-21)23(28)18-9-8-16-5-3-4-6-17(16)11-18/h3-13,25,27H,1-2H3,(H,26,29) | - mPGES-1 IC_50_ : 3.3 ± 0.5 µM - sEH IC_50_: 1.5 ± 0.2 µM - HTC-116 IC_50_: 42 ± 1 µM - HaCat IC_50_: 31 ± 4 µM |
| 2c | InChI=1S/C24H20N2O4/c1-14-19(23(28)17-8-7-15-5-3-4-6-16(15)11-17)13-20(25-14)24(29)26-18-9-10-22(30-2)21(27)12-18/h3-13,25,27H,1-2H3,(H,26,29) | - mPGES-1 IC_50_ : 4.0 ± 1.1 µM - sEH IC_50_: 6.3 ± 1.1 µM - HTC-116 IC_50_: 44 ± 3 µM - HaCat IC_50_: 43 ± 2µM |
| 2d | 4-(2-naphthoyl)-N-(4-chloro-3-(hydroxymethyl)phenyl)-5-methyl-1H-pyrrole-2-carboxamide | - mPGES-1 IC_50_ : 4.5 ± 1.3 µM - sEH IC_50_: 7.6 ± 1.0 µM - HTC-116 IC_50_: 39 ± 2 µM - HaCat IC_50_: 22 ± 2 µM |

^a^ Brief description of screening procedure incl. reference.

**mPGES-1 activity assay:** Microsomes from A549 cells, which contain the enzyme mPGES-1, were used in an experiment to study the production of PGE2. The A549 cells were first stimulated with IL-1β for 48 hours, then harvested and broken down by sonication. The resulting cell lysate was subjected to two rounds of centrifugation to isolate the microsomal fraction. The microsomes were diluted and placed in a 96-well plate, where they were preincubated with various compounds or a control solution. The production of PGE2 was triggered by adding a substrate, and the reaction was stopped after one minute. PGE2 was then extracted and quantified using HPLC.

**sEH activity assay:** The experiment involved using human recombinant sEH, which was diluted in a bis-Tris buffer and pre-incubated with either the test compounds, a reference inhibitor (AUDA), or a control solution. The reaction was initiated by adding a small amount of a no-fluorescent substrate called PHOME. When sEH hydrolyzes PHOME, it triggers a chemical reaction that ultimately produces a fluorescent compound. The fluorescence emitted by this compound was measured using a plate reader, which allowed the researchers to analyze the enzyme activity and determine the effectiveness of the test compounds in inhibiting sEH.

**Experiments on HTC-116 and HaCat cell lines:** The bioactivity and cellular responses to treatment 2d were evaluated by calculating a "cell survival index." This index combines results from cell viability tests (using the MTT assay) and cell counting. HCT-116 and HaCaT cells were grown in 96-well plates for 24 hours, after which they were treated with various concentrations of the compounds for an additional 48 hours. After treatment, cell viability was assessed by incubating the cells with MTT solution, which results in the formation of formazan crystals. These crystals were dissolved in DMSO, and the absorbance was measured to determine cell viability.
