# Supplementary material for: 5‐methyl‐2‐carboxamidepyrrole‐based novel dual mPGES‐1/sEH inhibitors as promising anticancer candidates
Source: Arch Pharm (Weinheim). 2024 Dec 18;358(1):e2400708. doi: 10.1002/ardp.202400708 (PMC11653428; doi:10.1002/ardp.202400708)
Supplement: Supplementary file 2 — Supporting information. [file ARDP-358-e2400708-s001.docx]

**SUPPORTING INFORMATION**

[**FIGURE S1.** 3-(4-dodecanoyl-1,3,5-trimethyl-1*H*-pyrrol-2-yl)propanoic acid mPGES-1 inhibitor **I** (IC_50_ = 0.80 μM).^1^ 3](#_Toc183432890)

[**TABLE S1.** Pyrrole alkanoic acid derivatives and inhibitory activity on mPGES-1.^1^ 3](#_Toc183432891)

[**FIGURE S2.** 3D docking poses of A) 5-methyl-4-phenoyl-1*H*-pyrrole-2-carboxamide and B) 5-methyl-4-naphtoyl-1*H*-pyrrole-2-carboxamide 4](#_Toc183432892)

[**FIGURE S3.** ^1^H NMR spectrum (400 MHz, CDCl_3_) of compound **8a**. 5](#_Toc183432893)

[**FIGURE S4.** ^13^C-DEPTq NMR spectrum (100 MHz, CDCl_3_) of compound **8a**. 5](#_Toc183432895)

[**FIGURE S5.** ^1^H NMR spectrum (400 MHz, CDCl_3_) of compound **8b**. 6](#_Toc183432896)

[**FIGURE S6.** ^13^C-DEPTq NMR spectrum (100 MHz, CDCl_3_) of compound **8b**. 6](#_Toc183432897)

[**FIGURE S7.** Expanded region of 2D ROESY spectrum of compound **8b**. 7](#_Toc183432898)

[**FIGURE S8.** ^1^H NMR spectrum (400 MHz, CDCl_3_) of compound **9a**. 8](#_Toc183432899)

[**FIGURE S9.** ^13^C-DEPTq NMR spectrum (100 MHz, CDCl_3_) of compound **9a**. 8](#_Toc183432900)

[**FIGURE S10.** ^1^H NMR spectrum (400 MHz, DMSO-*d_6_*) of compound **9b**. 9](#_Toc183432901)

[**FIGURE S11.** ^13^C-DEPTq NMR spectrum (100 MHz, DMSO-*d*_6_) of compound **9b**. 9](#_Toc183432902)

[**FIGURE S12.** ^1^H NMR spectrum (400 MHz, DMSO-*d*_6_) of compound **1a**. 10](#_Toc183432903)

[**FIGURE S13.** ^13^C-NMR spectrum (100 MHz, DMSO-*d*_6_) of compound **1a**. 10](#_Toc183432904)

[**FIGURE S14.** HRMS (ESI) spectrum of compound **1a**. 11](#_Toc183432905)

[**FIGURE S15.** HPLC analysis of compound **1a**. 11](#_Toc183432906)

[**FIGURE S16.** ^1^H NMR spectrum (400 MHz, DMSO-*d_6_*) of compound **1b**. 12](#_Toc183432907)

[**FIGURE S17.** ^13^C NMR spectrum (100 MHz, DMSO-*d*_6_) of compound **1b**. 12](#_Toc183432908)

[**FIGURE S18.** HRMS (ESI) spectrum of compound **1b**. 13](#_Toc183432909)

[**FIGURE S19.** HPLC analysis of compound **1b**. 13](#_Toc183432910)

[**FIGURE S20.** ^1^H NMR spectrum (400 MHz, DMSO-*d_6_*) of compound **1c**. 14](#_Toc183432911)

[**FIGURE S21.** ^13^C NMR spectrum (100 MHz, DMSO-*d*_6_) of compound **1c**. 14](#_Toc183432912)

[**FIGURE S22.** HRMS (ESI) spectrum of compound **1c**. 15](#_Toc183432913)

[**FIGURE S23.** HPLC analysis of compound **1c**. 15](#_Toc183432914)

[**FIGURE S24.** ^1^H NMR spectrum (400 MHz, DMSO-*d_6_*) of compound **1d**. 16](#_Toc183432915)

[**FIGURE S25.** ^13^C NMR (100 MHz, DMSO-*d_6_*) spectrum of compound **1d**. 16](#_Toc183432916)

[**FIGURE S26.** HRMS (ESI) spectrum of compound **1d**. 17](#_Toc183432917)

[**FIGURE S27.** HPLC analysis of compound **1d**. 17](#_Toc183432918)

[**FIGURE S28.** ^1^H NMR spectrum (400 MHz, DMSO-*d*_6_) of compound **1e**. 18](#_Toc183432919)

[**FIGURE S29.** ^13^C-DEPTq NMR spectrum (100 MHz, DMSO-*d_6_*) of compound **1e**. 18](#_Toc183432920)

[**FIGURE S30.** HRMS (ESI) spectrum of compound **1e**. 19](#_Toc183432921)

[**FIGURE S31.** HPLC analysis of compound **1e**. 19](#_Toc183432922)

[**FIGURE S32.** ^1^H NMR spectrum (400 MHz, DMSO-*d_6_*) of compound **1f**. 20](#_Toc183432923)

[**FIGURE S33.** ^13^C-DEPTq NMR spectrum (100 MHz, DMSO-*d*_6_) of compound **1f**. 20](#_Toc183432924)

[**FIGURE S34.** HRMS (ESI) spectrum of compound **1f**. 21](#_Toc183432925)

[**FIGURE S35.** HPLC analysis of compound **1f**. 21](#_Toc183432926)

[**FIGURE S36.** ^1^H NMR spectrum (400 MHz, DMSO-*d_6_*) of compound **2a**. 22](#_Toc183432927)

[**FIGURE S37.** ^13^C-DEPTq NMR spectrum (100 MHz, DMSO-*d*_6_) of compound **2a**. 22](#_Toc183432928)

[**FIGURE S38.** HRMS (ESI) spectrum of compound **2a**. 23](#_Toc183432929)

[**FIGURE S39.** HPLC analysis of compound **2a**. 23](#_Toc183432930)

[**FIGURE S40.** ^1^H NMR spectrum (400 MHz, DMSO-*d*_6_) of compound **2b**. 24](#_Toc183432931)

[**FIGURE S41.** ^13^C-DEPTq NMR spectrum (100 MHz, DMSO-*d*_6_) of compound **2b**. 24](#_Toc183432932)

[**FIGURE S42.** HRMS (ESI) spectrum of compound **2b**. 25](#_Toc183432933)

[**FIGURE S43.** HPLC analysis of compound **2b**. 25](#_Toc183432934)

[**FIGURE S44.** ^1^H NMR spectrum (400 MHz, DMSO-*d_6_*) of compound **2c**. 26](#_Toc183432935)

[**FIGURE S45.** ^13^C-DEPTq NMR spectrum (100 MHz, DMSO-*d*_6_) of compound **2c**. 26](#_Toc183432936)

[**FIGURE S46.** HRMS (ESI) spectrum of compound **2c**. 27](#_Toc183432937)

[**FIGURE S47.** HPLC analysis of compound **2c**. 27](#_Toc183432938)

[**FIGURE S48.** ^1^H NMR spectrum (400 MHz, DMSO-*d_6_*) of compound **2d**. 28](#_Toc183432939)

[**FIGURE S49.** ^13^C-DEPTq NMR spectrum (100 MHz, DMSO-*d*_6_) of compound **2d**. 28](#_Toc183432940)

[**FIGURE S50.** HRMS (ESI) spectrum of compound **2d**. 29](#_Toc183432941)

[**FIGURE S51.** HPLC analysis of compound **2d**. 29](#_Toc183432942)

[**FIGURE S52.** Residual activity of mPGES-1(%) after incubation with compounds **1a**-**f** and **2a**-**d** at a concentration of 1 μM. 30](#_Toc183432943)

[**FIGURE S53.** IC_50_ curves for compounds **2b**(A), **2c**(B), **2d**(C) on mPGES-1 30](#_Toc183432944)

[**FIGURE S54.** IC_50_ curves for compounds **1f**(A), **2b**(B), **2c**(C), **2d**(D) on sEH. 31](#_Toc183432945)

[**TABLE S2.** H-bonds and π-π interactions reported for **1f**, **2b**-**2d** in the mPGES-1 and sEH binding sites. 31](#_Toc183432946)

[**REFERENCES** 32](#_Toc183432947)

# **FIGURE S1.** 3-(4-dodecanoyl-1,3,5-trimethyl-1*H*-pyrrol-2-yl)propanoic acid mPGES-1 inhibitor I (IC_50_ = 0.80 μM).^1^

# **TABLE S1.** Pyrrole alkanoic acid derivatives and inhibitory activity on mPGES-1.^1^

| Reported compounds^[2]^ | | Structure | Inhibition of mPGES-1 from A549 cells at 3.3 μM [%] | Inhibition of human recombinant mPGES-1 at 1μM [%] |
| --- | --- | --- | --- | --- |
| **I** |  | | 74±8 (n=5) | 54±8 (n=7) |
| **II** |  | | n.a. | n.a. |
| **III** |  | | n.a. | n.a. |
| **IV** |  | | 55 | n.a. |
| **V** |  | | 62 | n.a. |
| **VI** |  | | 63 | 91 |
| **VII** |  | | 35 | 97 |
| **VIII** |  | | n.a. | 97 |
| **IX** |  | | 23 | n.a. |

**
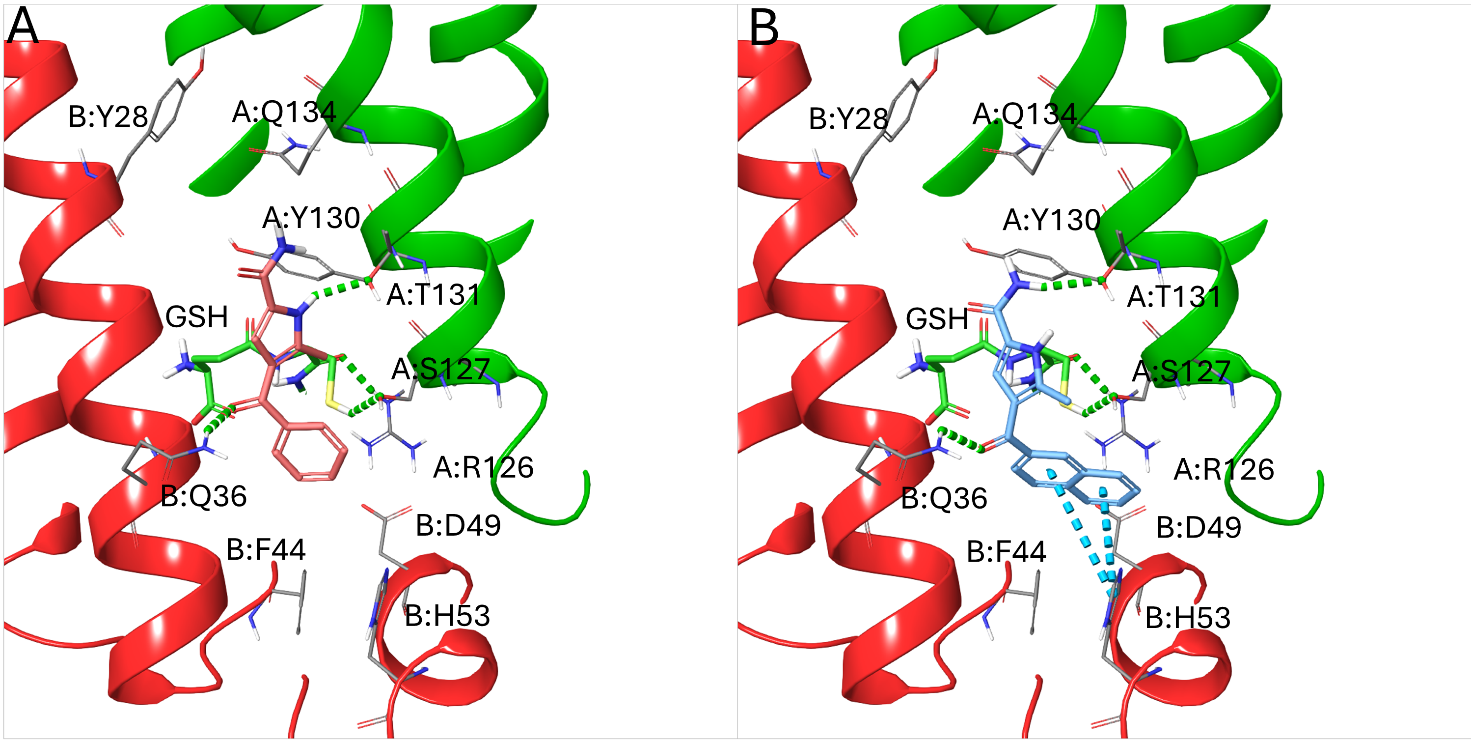
**

**FIGURE S2.** 3D docking poses of A) 5-methyl-4-phenoyl-1*H*-pyrrole-2-carboxamide (colored by atom type: C dark orange, O red, N blue, polar H white), B) 5-methyl-4-naphtoyl-1*H*-pyrrole-2-carboxamide (colored by atom type: C light blue, O red, N blue, polar H white) in the mPGES-1 binding site (chains A and B reported in green and red ribbons, respectively; GSH cofactor colored by atom type: C green, O red, N blue, S yellow, polar H white, residues colored by atom type: C grey, O red, N blue, polar H white). H-bonds and π-π interactions are reported in green and cyan dotted lines, respectively.


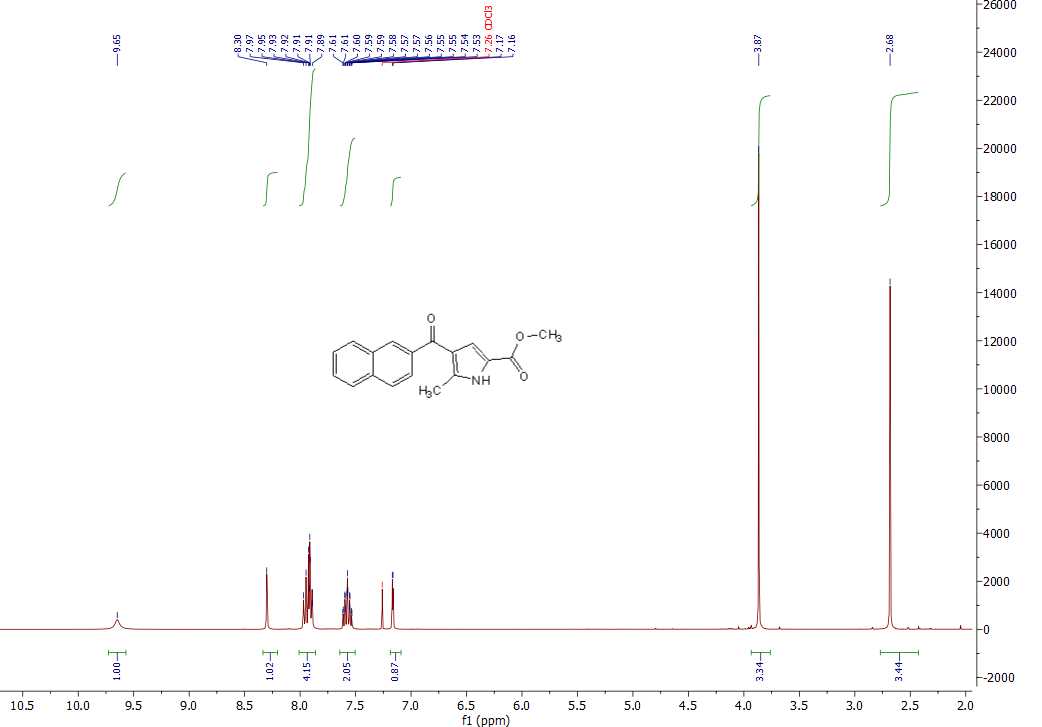


# **FIGURE S3.** ^1^H NMR spectrum (400 MHz, CDCl_3_) of compound **8a**.

**.
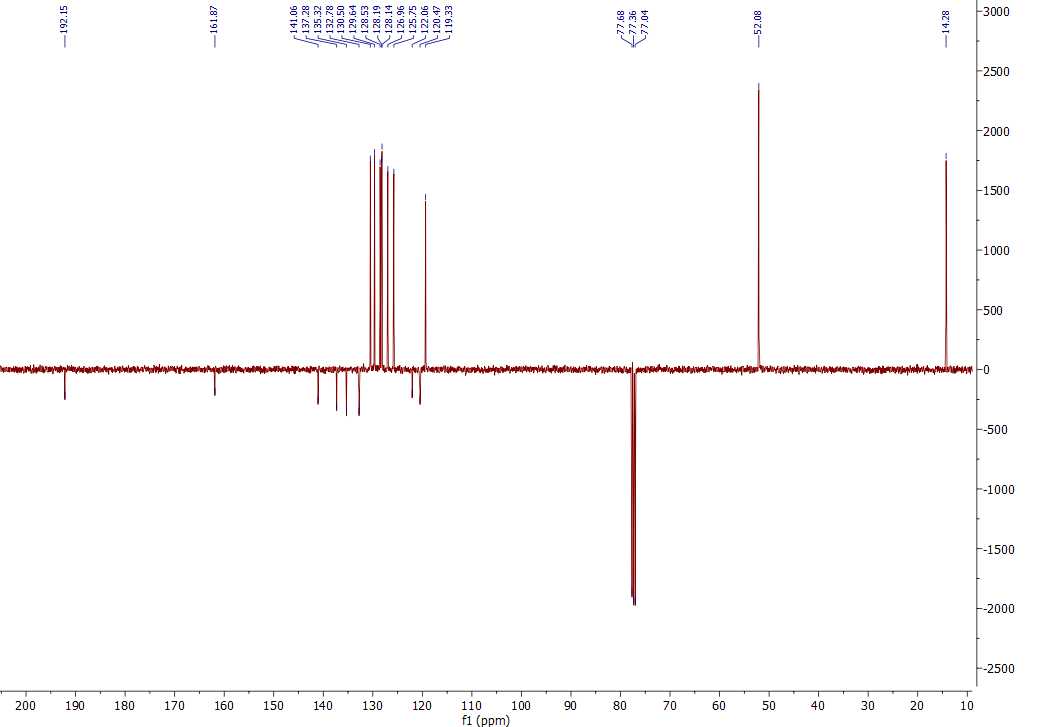
**

**FIGURE S4.** ^13^C-DEPTq NMR spectrum (100 MHz, CDCl_3_) of compound **8a**.


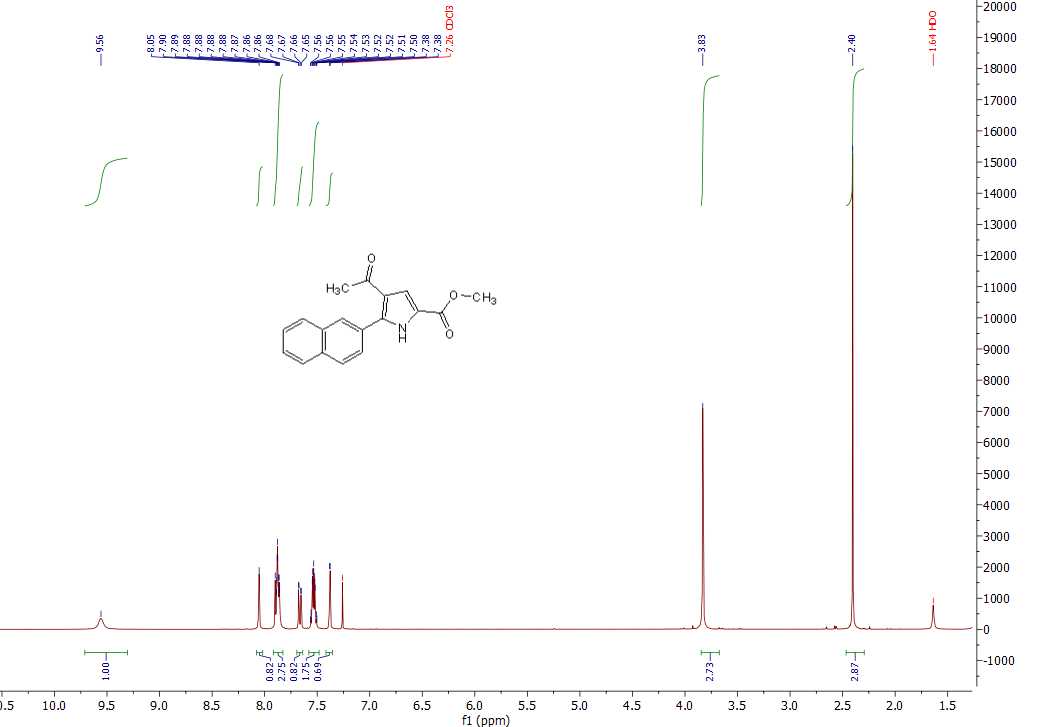


#
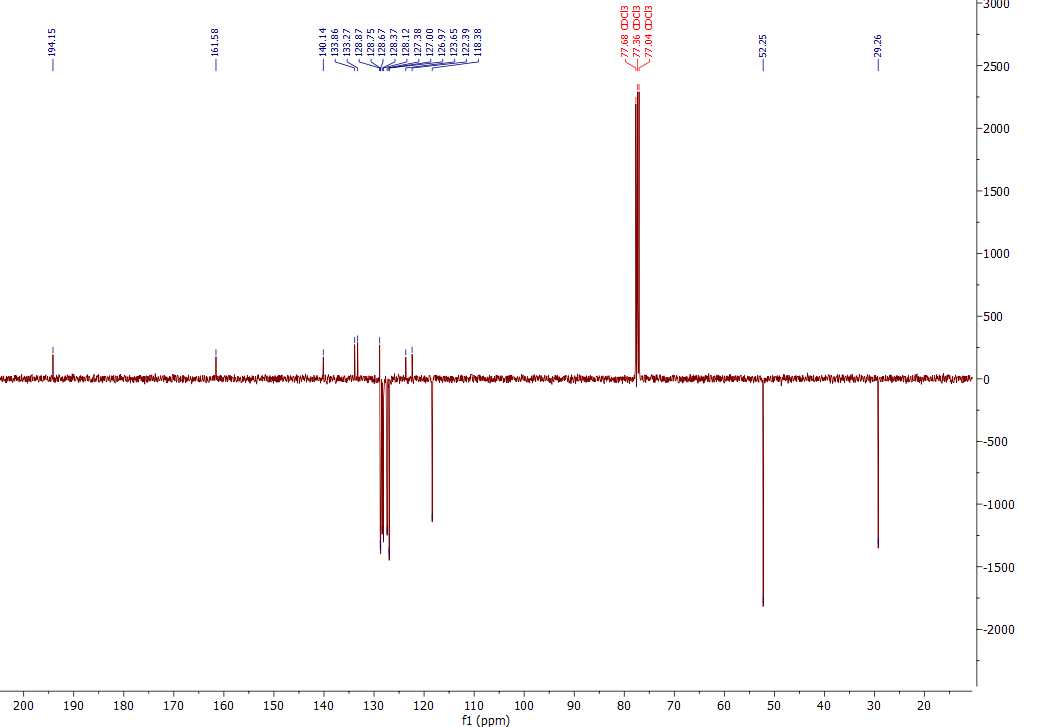
**FIGURE S5.** ^1^H NMR spectrum (400 MHz, CDCl_3_) of compound **8b**.

**FIGURE S6.** ^13^C-DEPTq NMR spectrum (100 MHz, CDCl_3_) of compound **8b**.

**
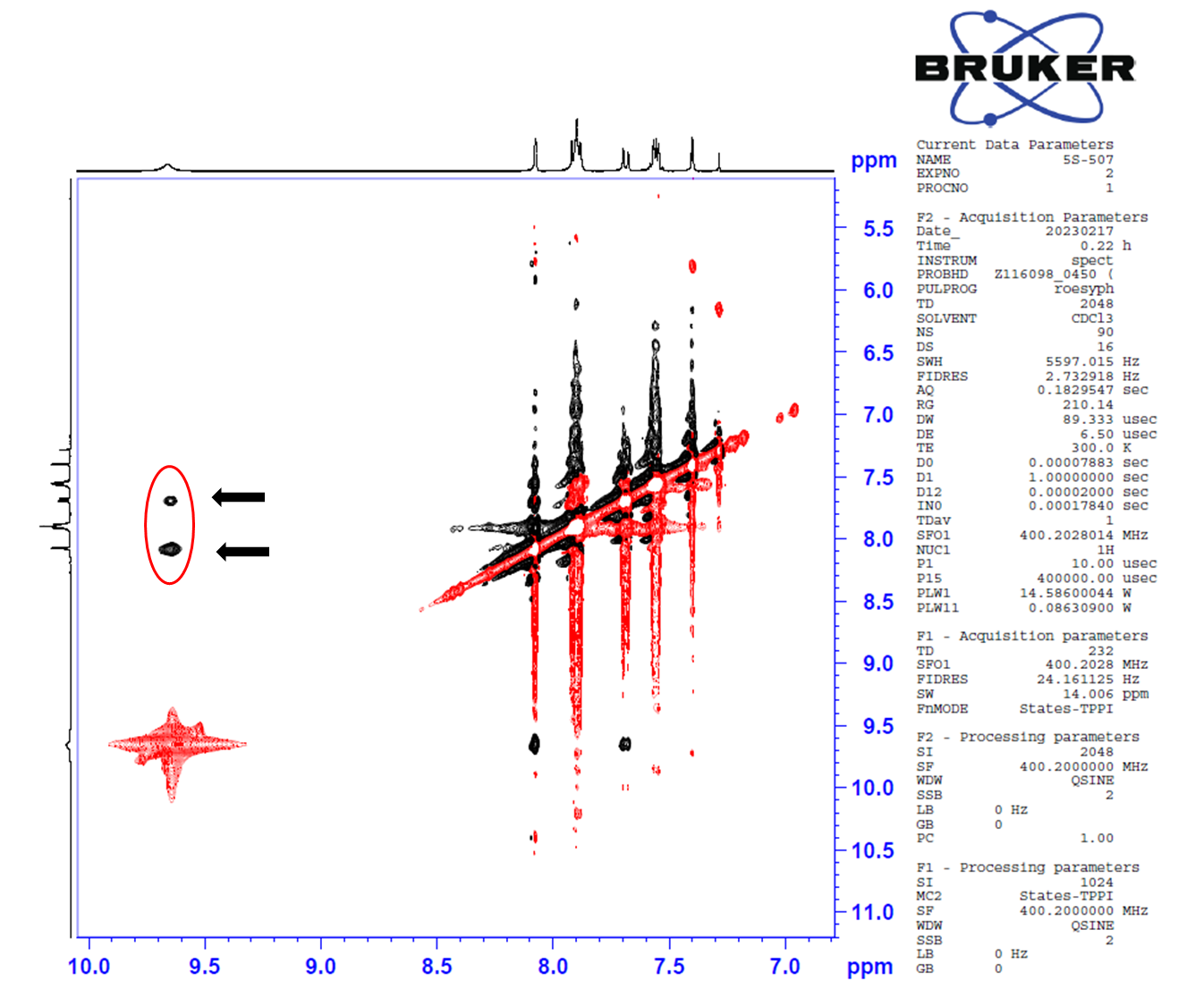
**

**FIGURE S7.** Expanded region of 2D ROESY spectrum of compound **8b**. Diagnostic ROE signals were evidenced with an arrow. 2D ROESY experiments were performed on a AscendTM Bruker 400 MHz spectrometer at 298 K. 2D spectra were recorded in the phase-sensitive mode, and data block sizes were 2048 addresses in t2 and 256 equidistant t1 values. A mixing time of 400 ms was used. Spectra were transformed and integrated using the Bruker program Topspin 3.6.2.


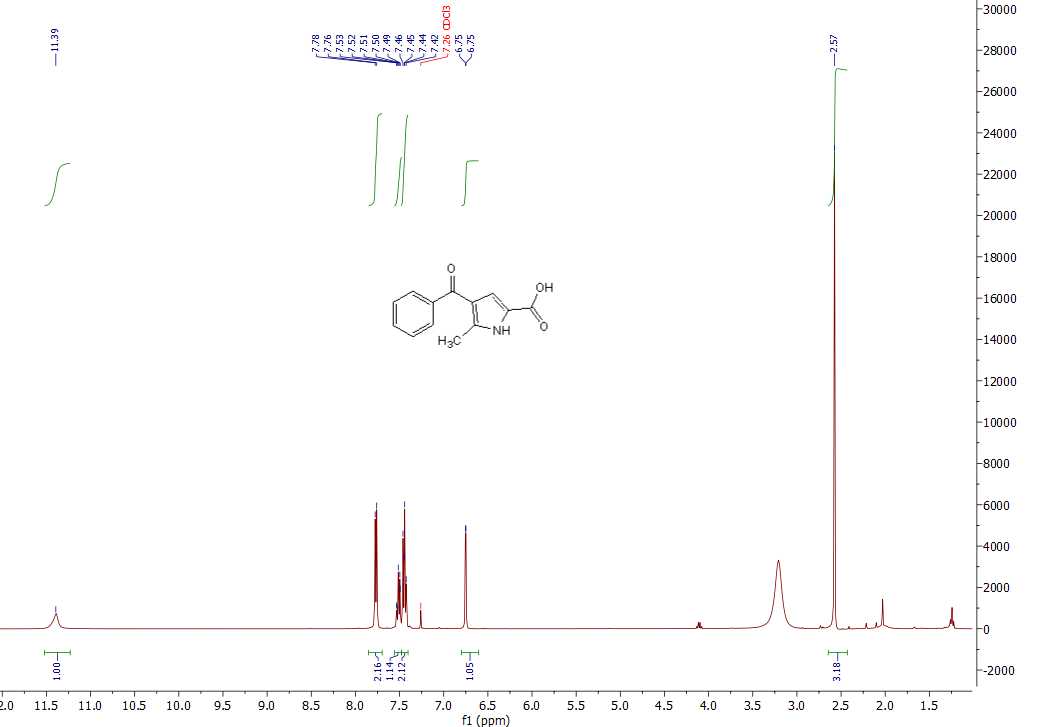


# **FIGURE S8.** ^1^H NMR spectrum (400 MHz, CDCl_3_) of compound **9a**.


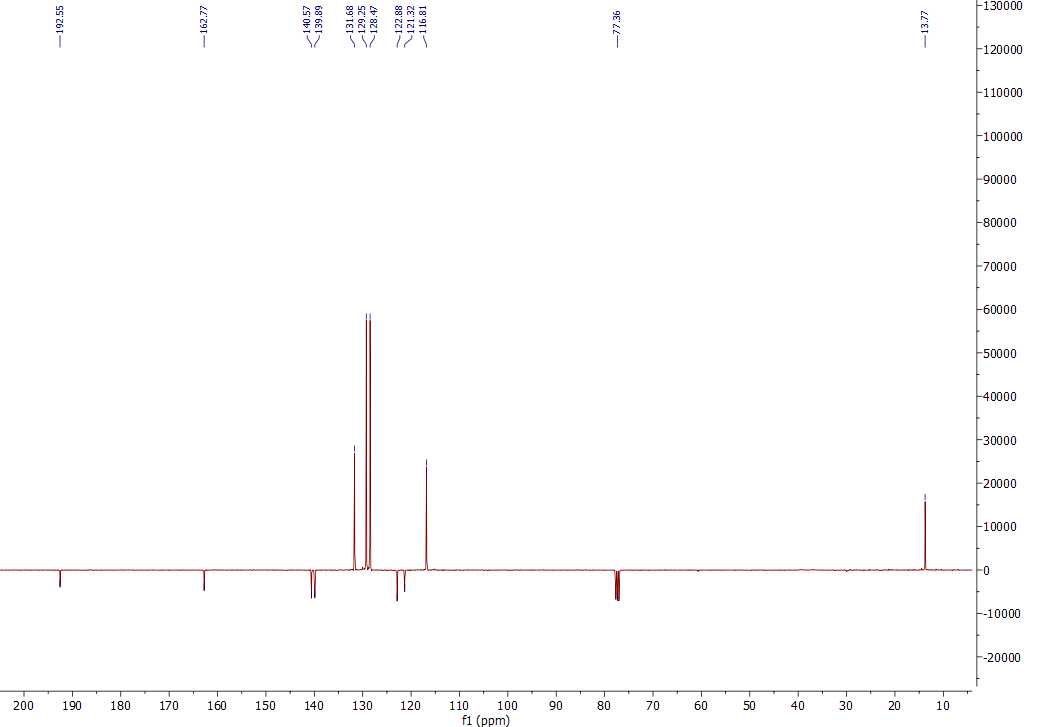


# **FIGURE S9.** ^13^C-DEPTq NMR spectrum (100 MHz, CDCl_3_) of compound **9a**.


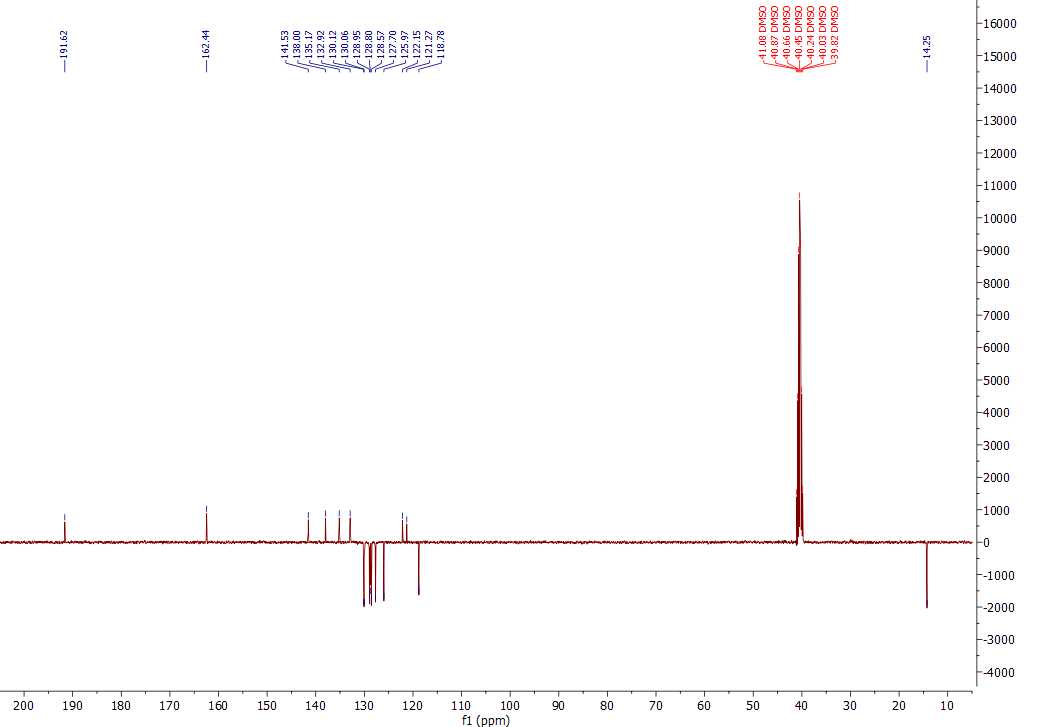

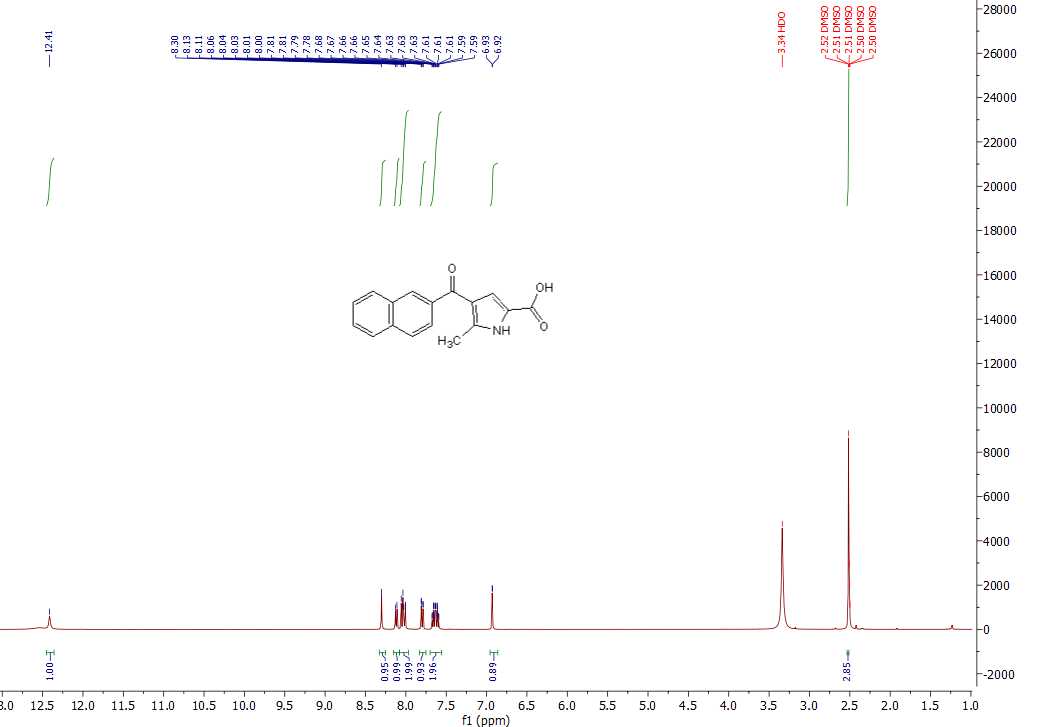


# **FIGURE S10.** ^1^H NMR spectrum (400 MHz, DMSO-*d_6_*) of compound **9b**.

# **FIGURE S11.** ^13^C-DEPTq NMR spectrum (100 MHz, DMSO-*d*_6_) of compound **9b**.


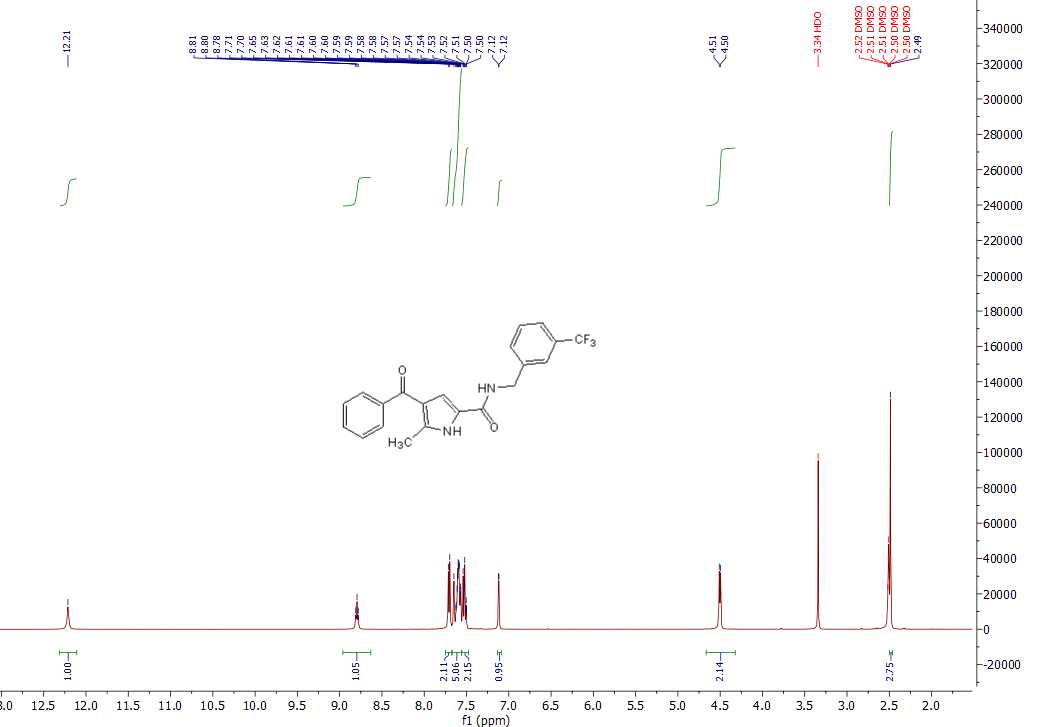


# **FIGURE S12.** ^1^H NMR spectrum (400 MHz, DMSO-*d*_6_) of compound **1a**.


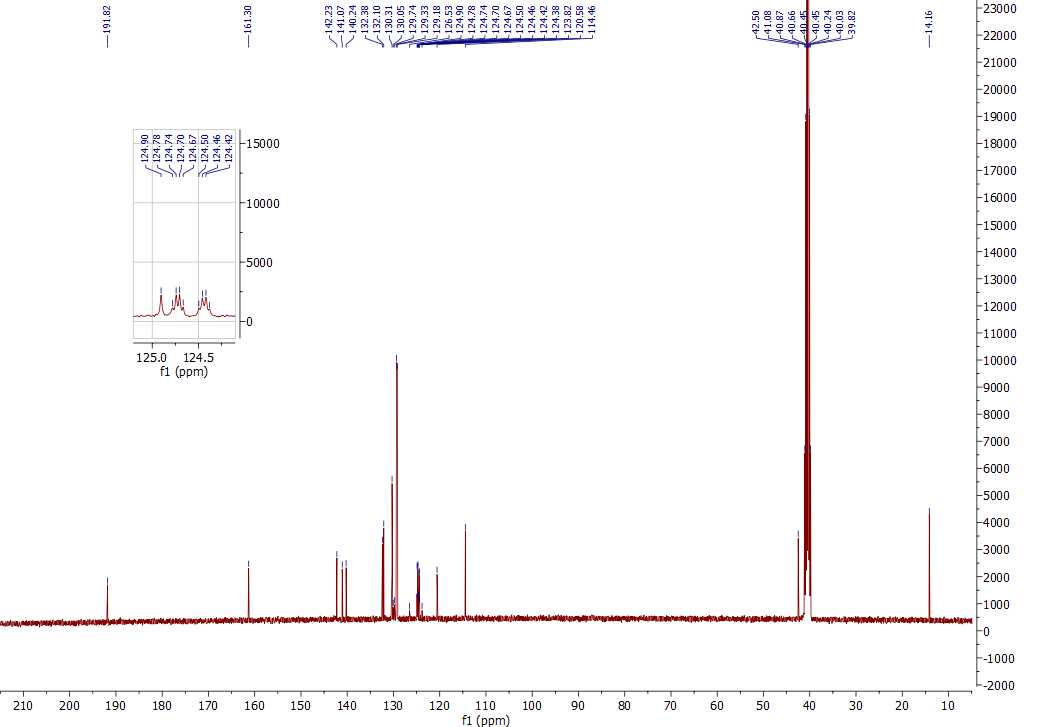


# **FIGURE S13.** ^13^C-NMR spectrum (100 MHz, DMSO-*d*_6_) of compound **1a**.


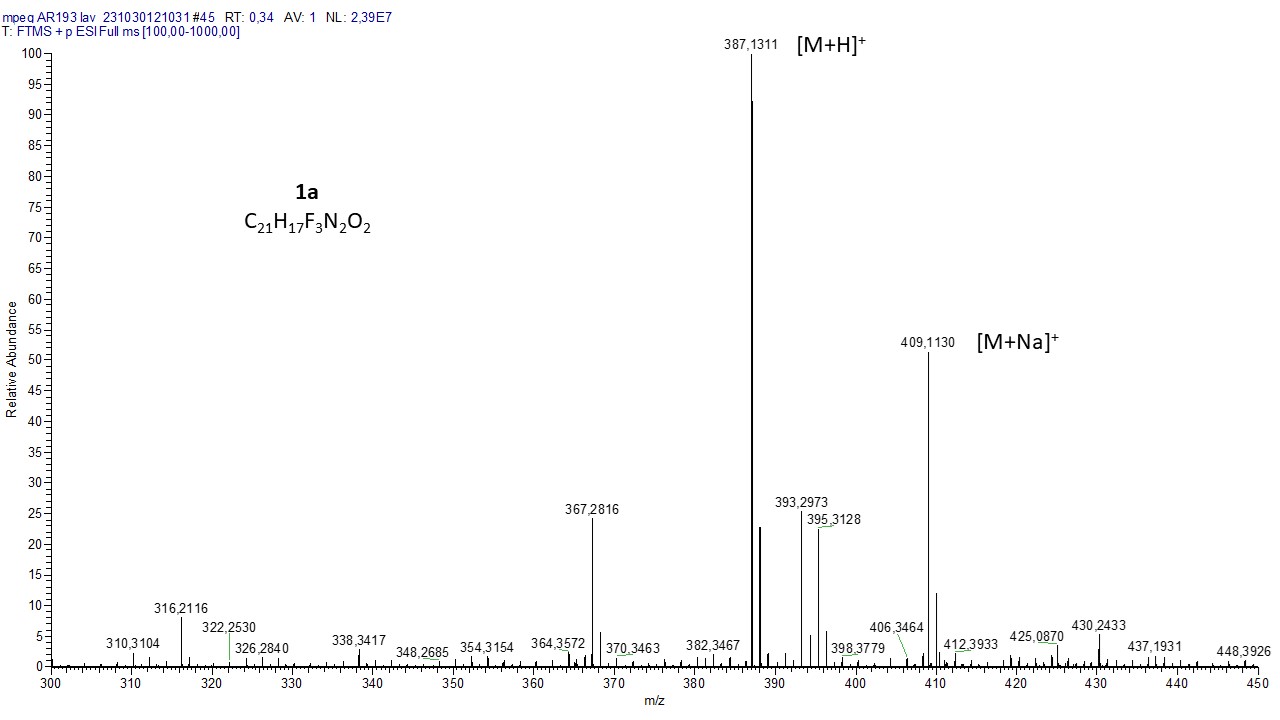


# **FIGURE S14.** HRMS (ESI) spectrum of compound **1a**.


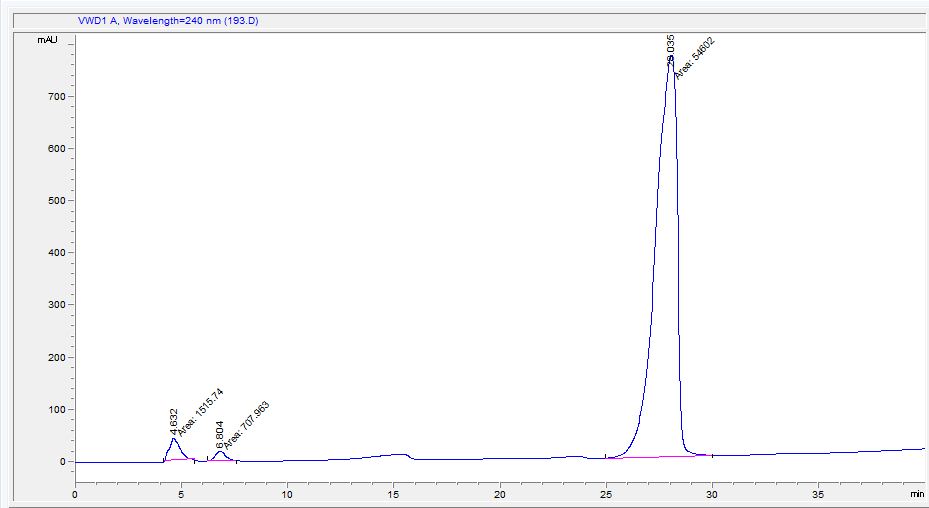


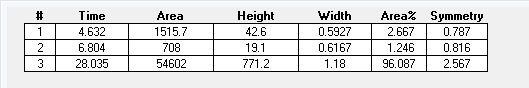


# **FIGURE S15.** HPLC analysis of compound **1a**.


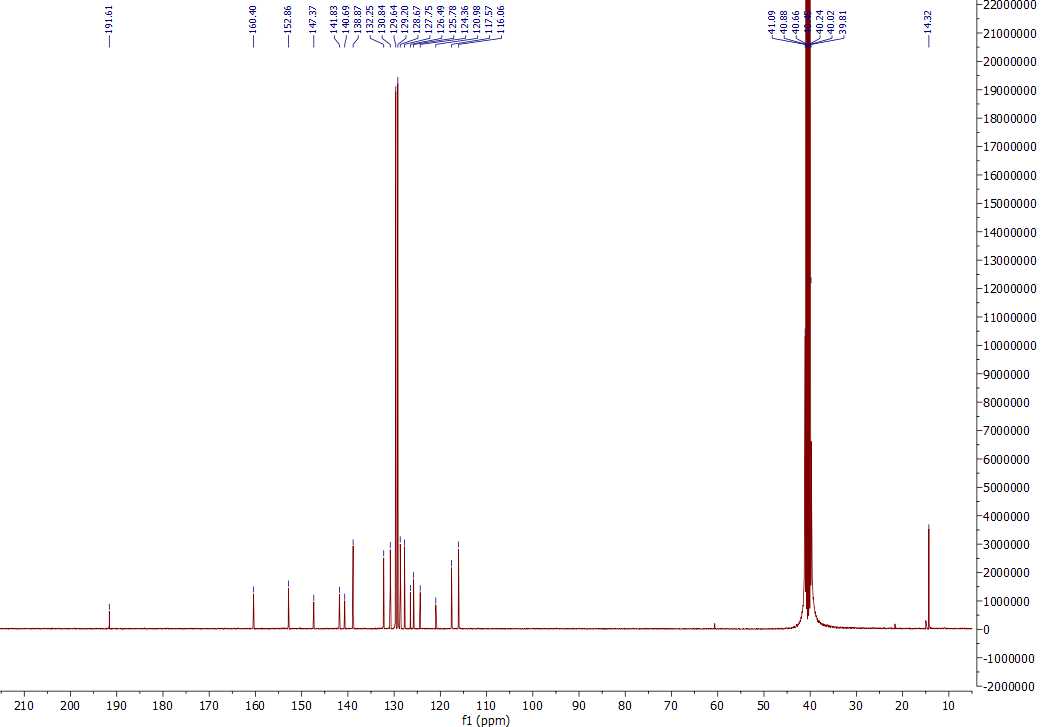

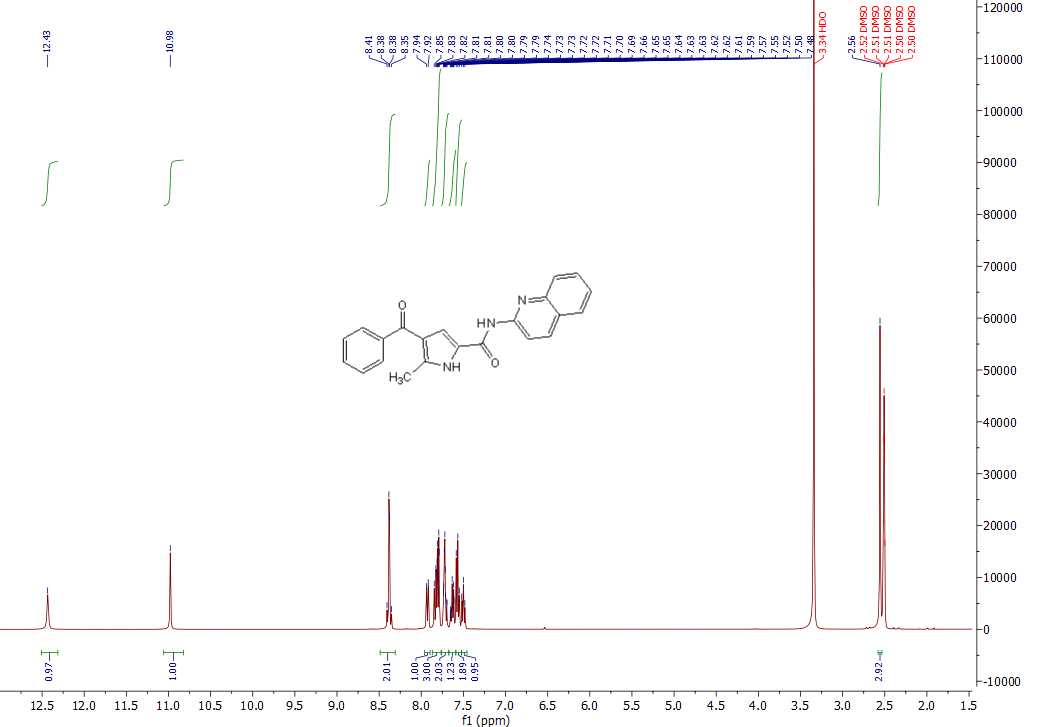


# **FIGURE S16.** ^1^H NMR spectrum (400 MHz, DMSO-*d_6_*) of compound **1b**.

# **FIGURE S17.** ^13^C NMR spectrum (100 MHz, DMSO-*d*_6_) of compound **1b**.


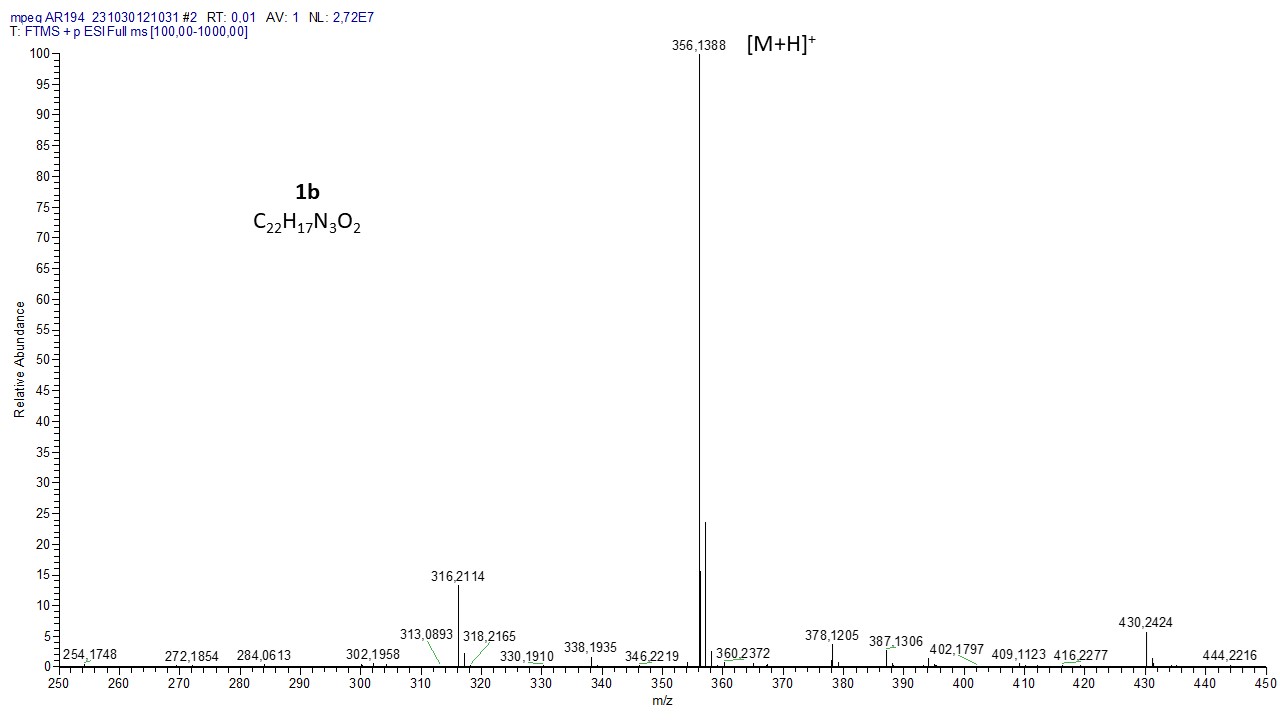


# **FIGURE S18.** HRMS (ESI) spectrum of compound **1b**.


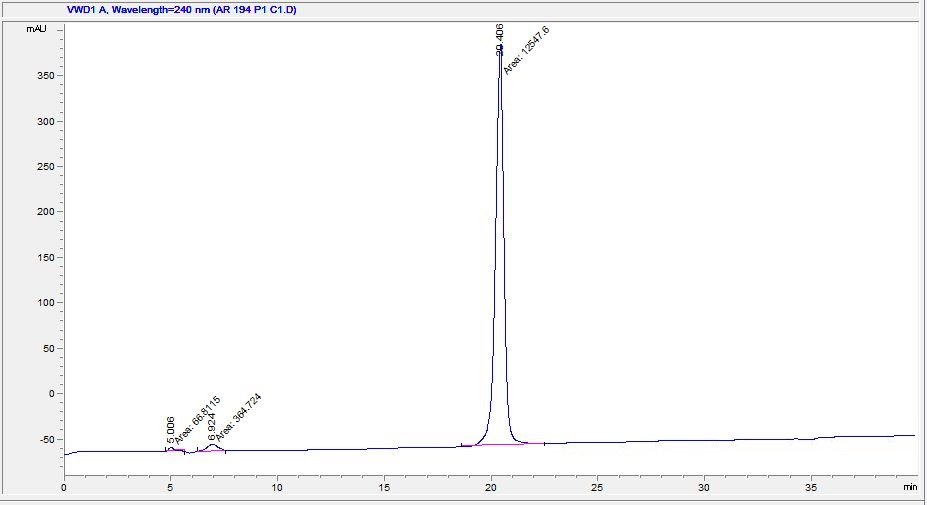


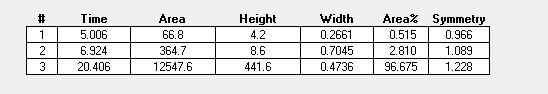


# **FIGURE S19.** HPLC analysis of compound **1b**.


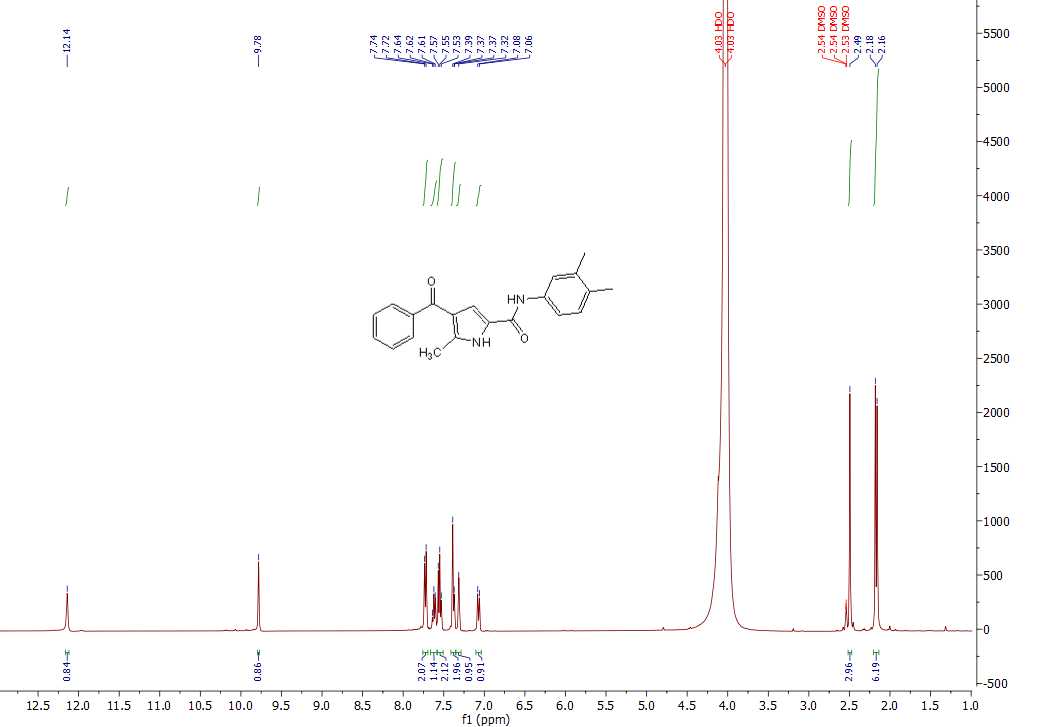


# **FIGURE S20.** ^1^H NMR spectrum (400 MHz, DMSO-*d_6_*) of compound **1c**.


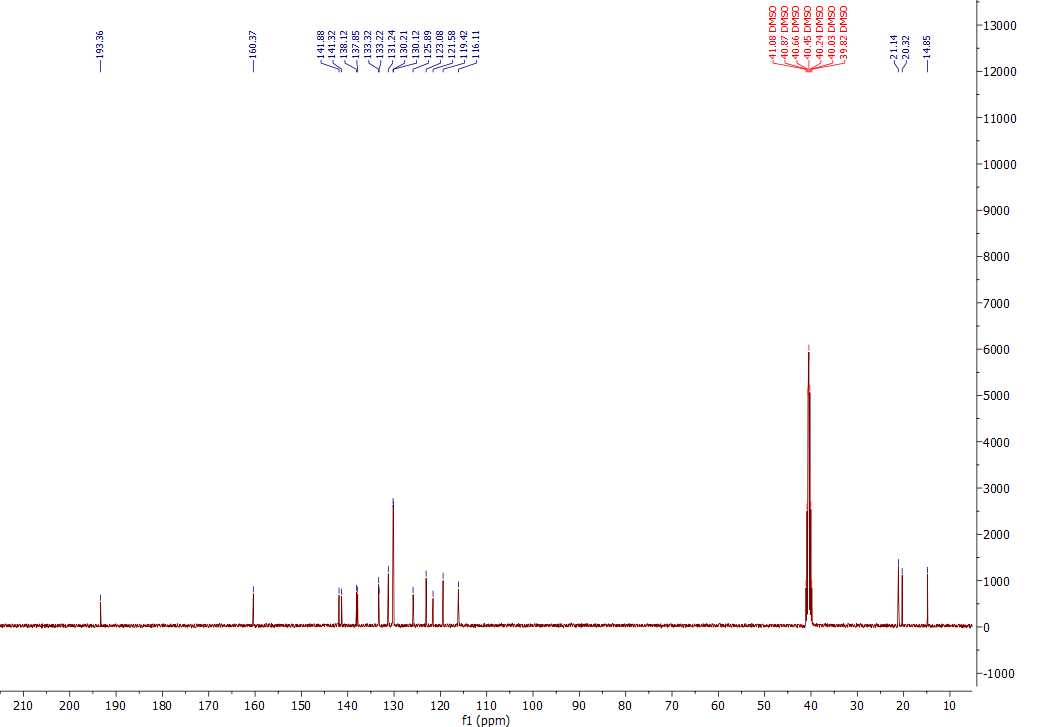


# **FIGURE S21.** ^13^C NMR spectrum (100 MHz, DMSO-*d*_6_) of compound **1c**.


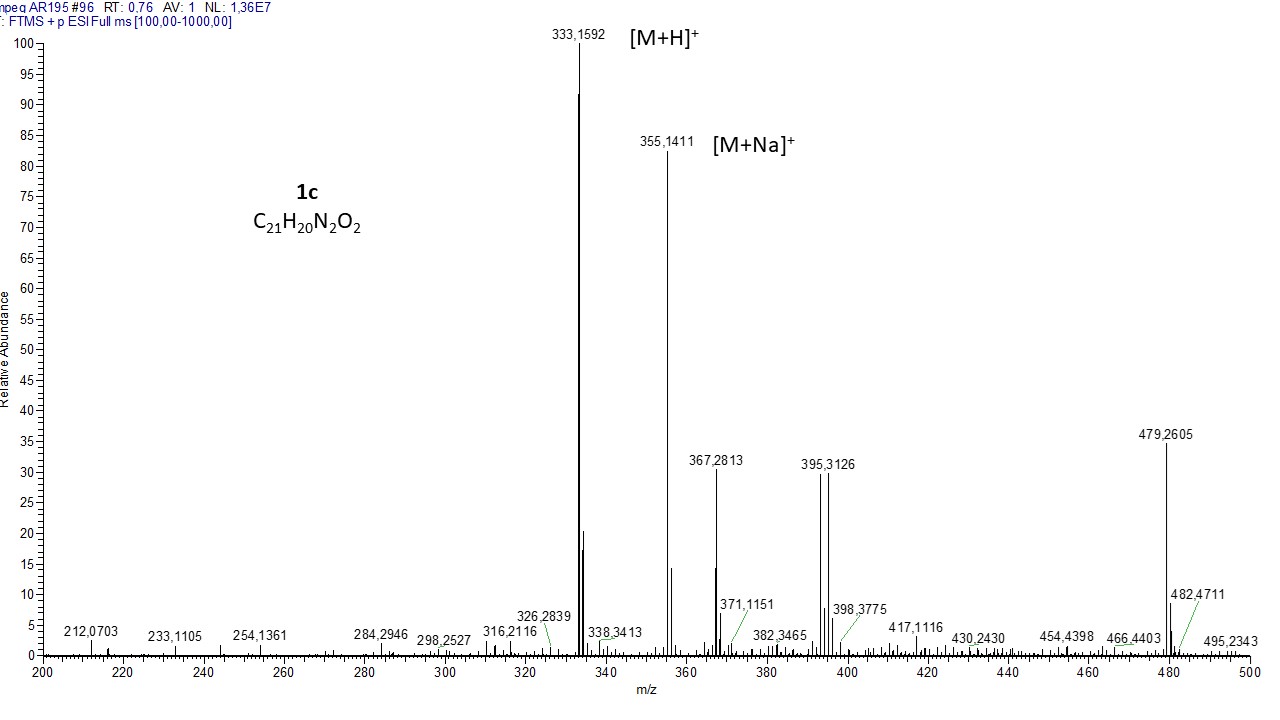


# **FIGURE S22.** HRMS (ESI) spectrum of compound **1c**.


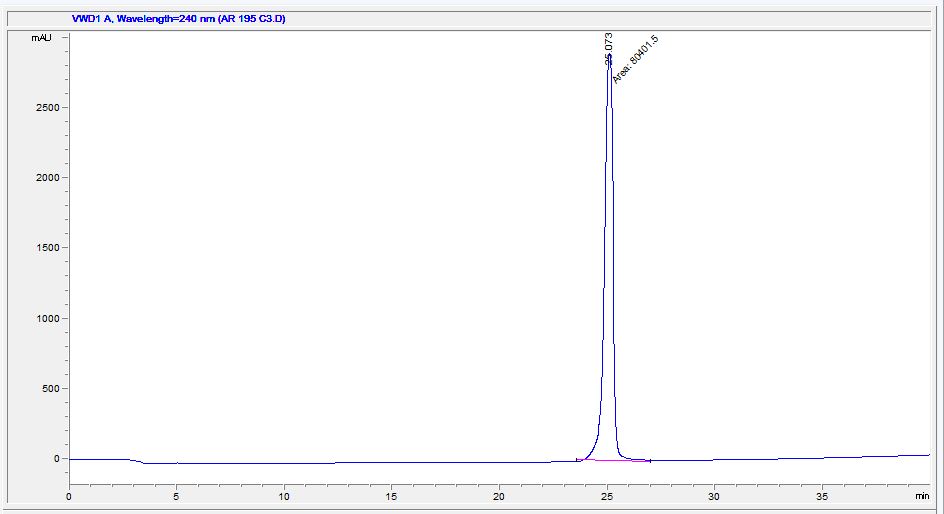


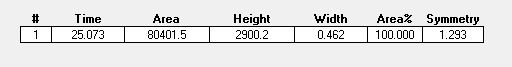


# **FIGURE S23.** HPLC analysis of compound **1c**.


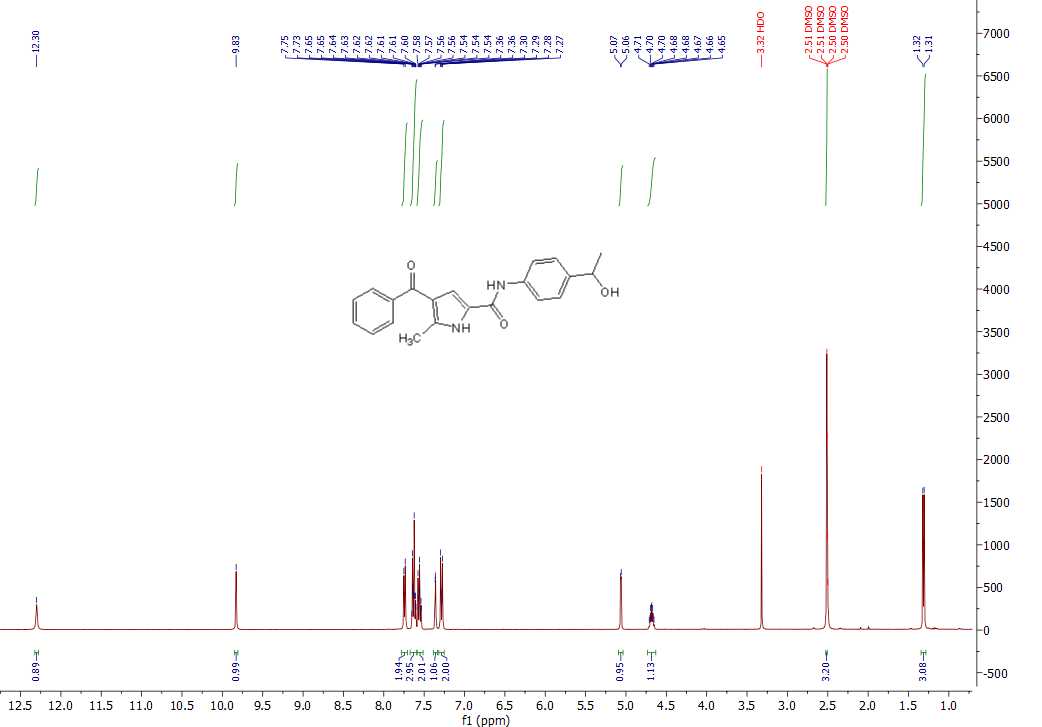


# **FIGURE S24.** ^1^H NMR spectrum (400 MHz, DMSO-*d_6_*) of compound **1d**.


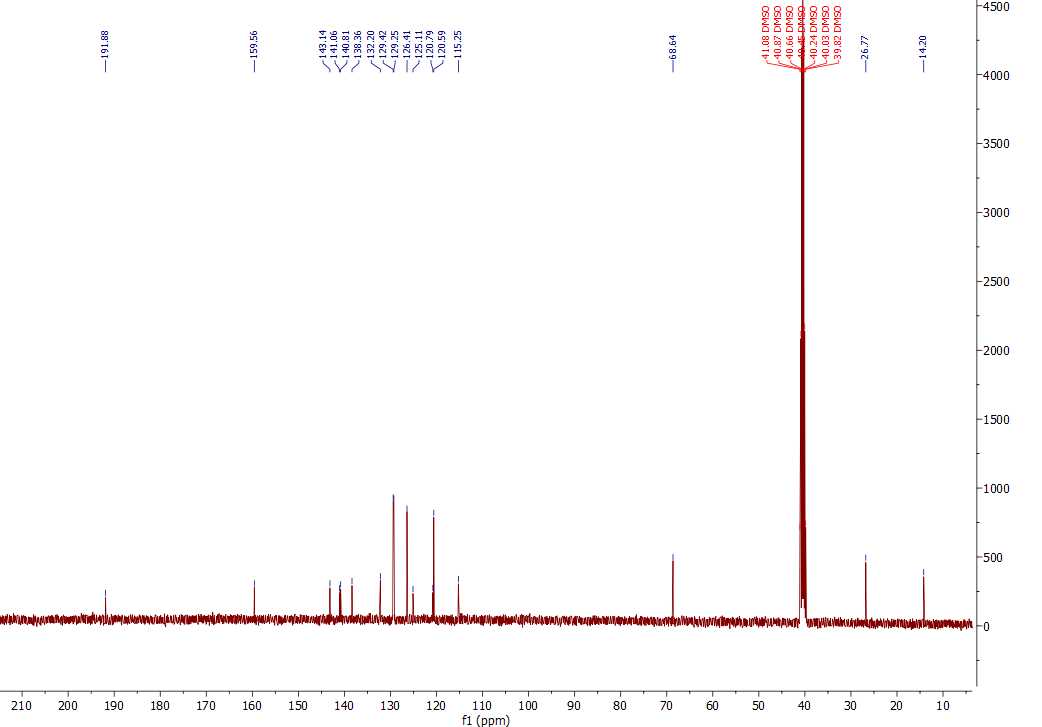


# **FIGURE S25.** ^13^C NMR (100 MHz, DMSO-*d_6_*) spectrum of compound **1d**.


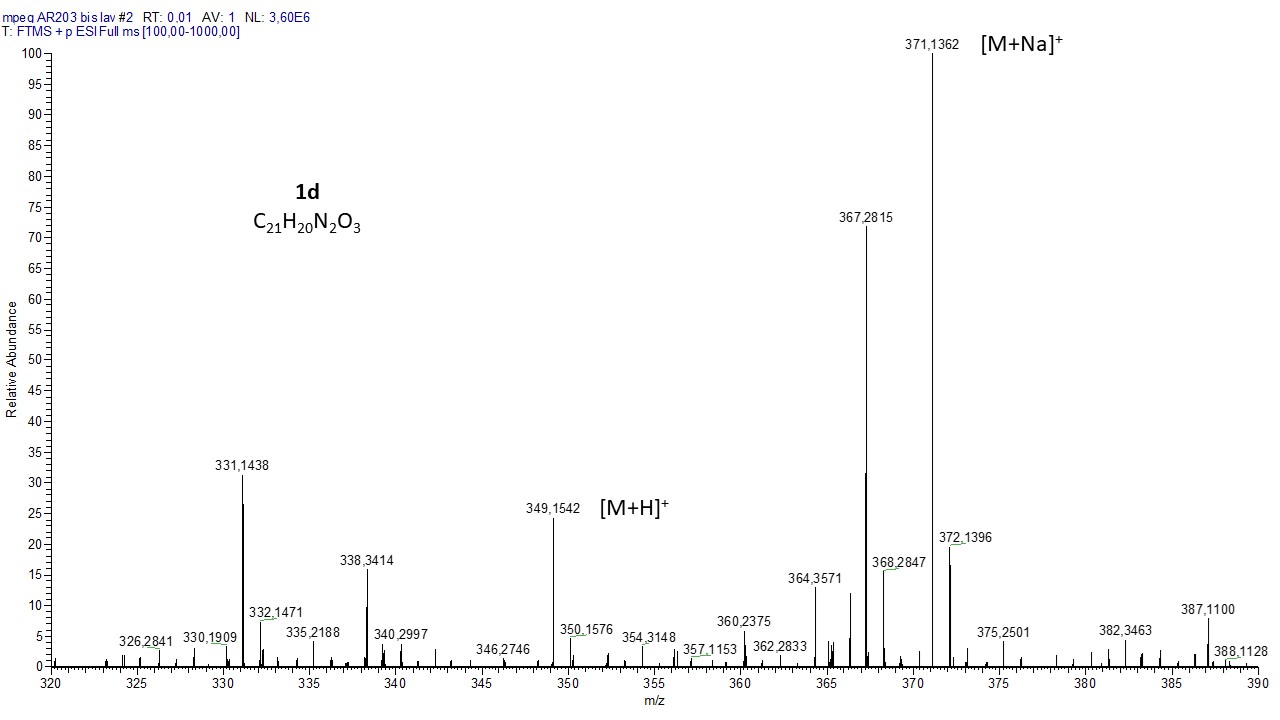


# **FIGURE S26.** HRMS (ESI) spectrum of compound **1d**.


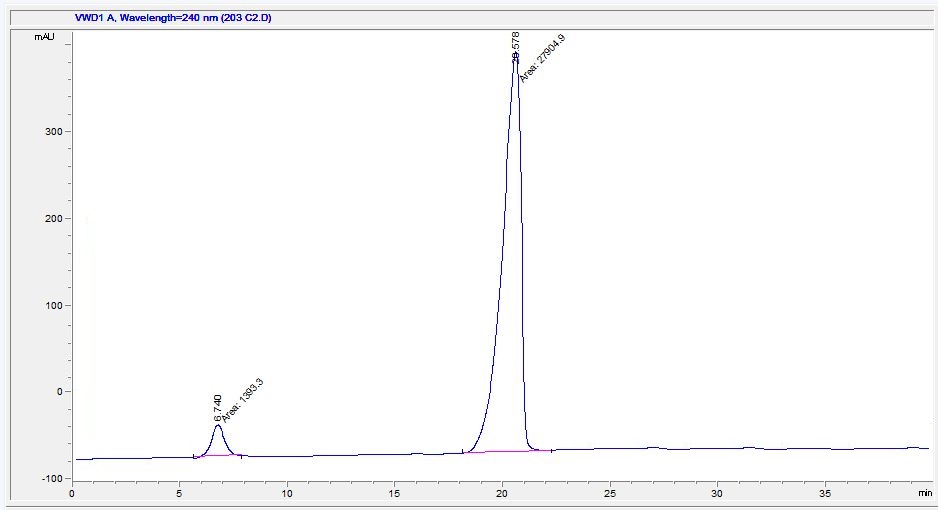


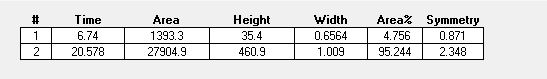


# **FIGURE S27.** HPLC analysis of compound **1d**.


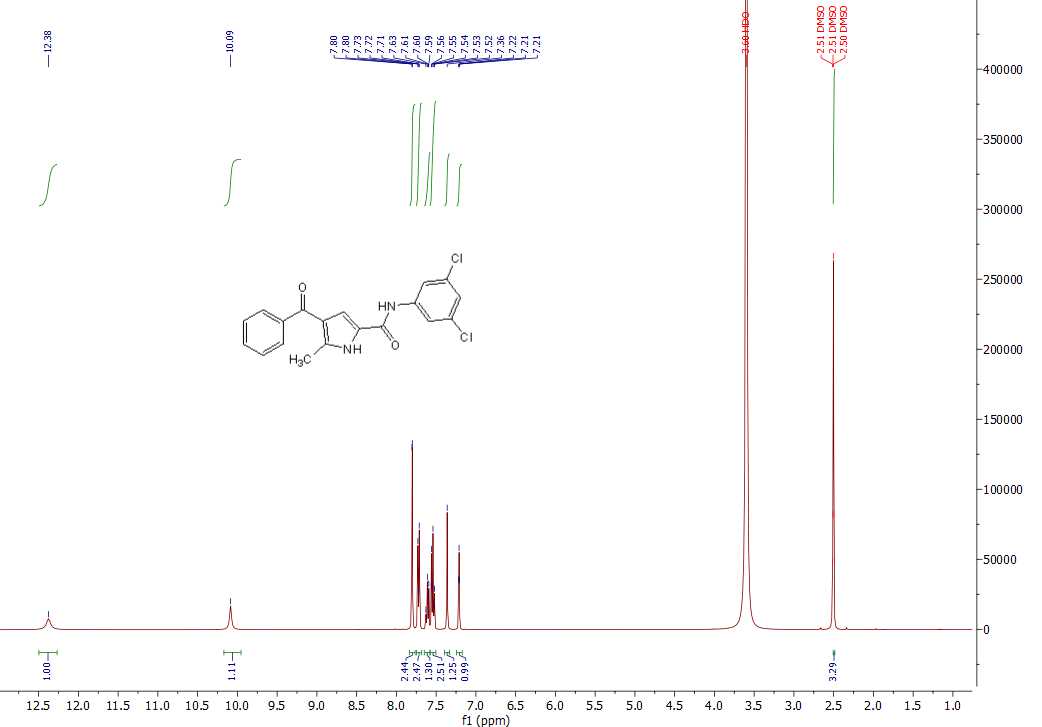


# **FIGURE S28.** ^1^H NMR spectrum (400 MHz, DMSO-*d*_6_) of compound **1e**.


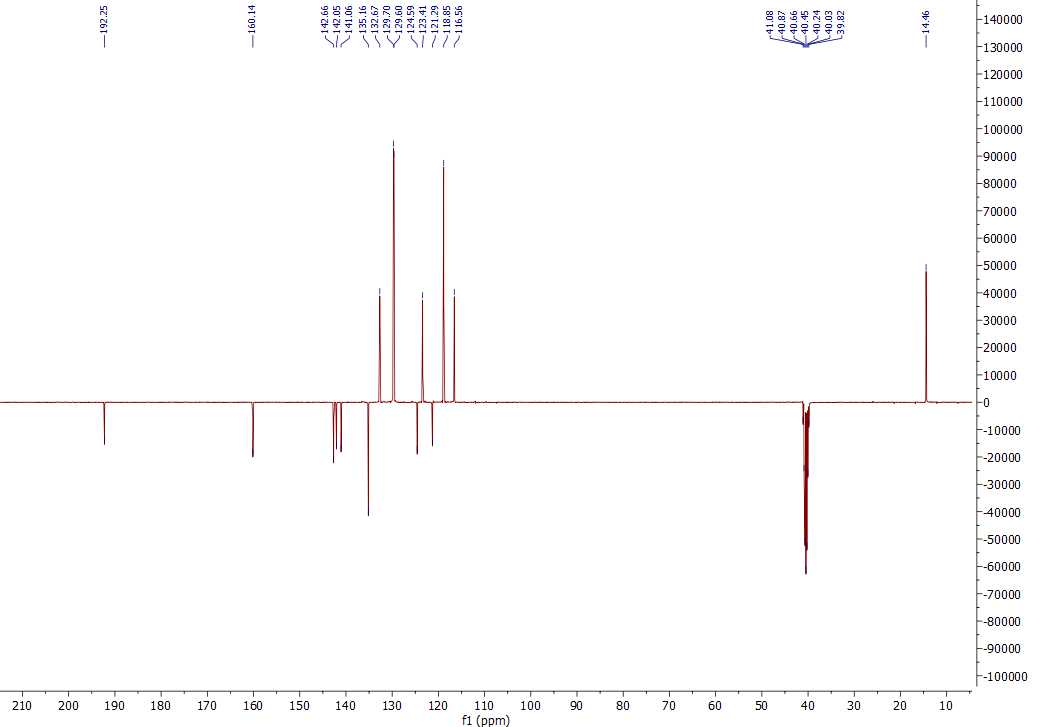


# **FIGURE S29.** ^13^C-DEPTq NMR spectrum (100 MHz, DMSO-*d_6_*) of compound **1e**.


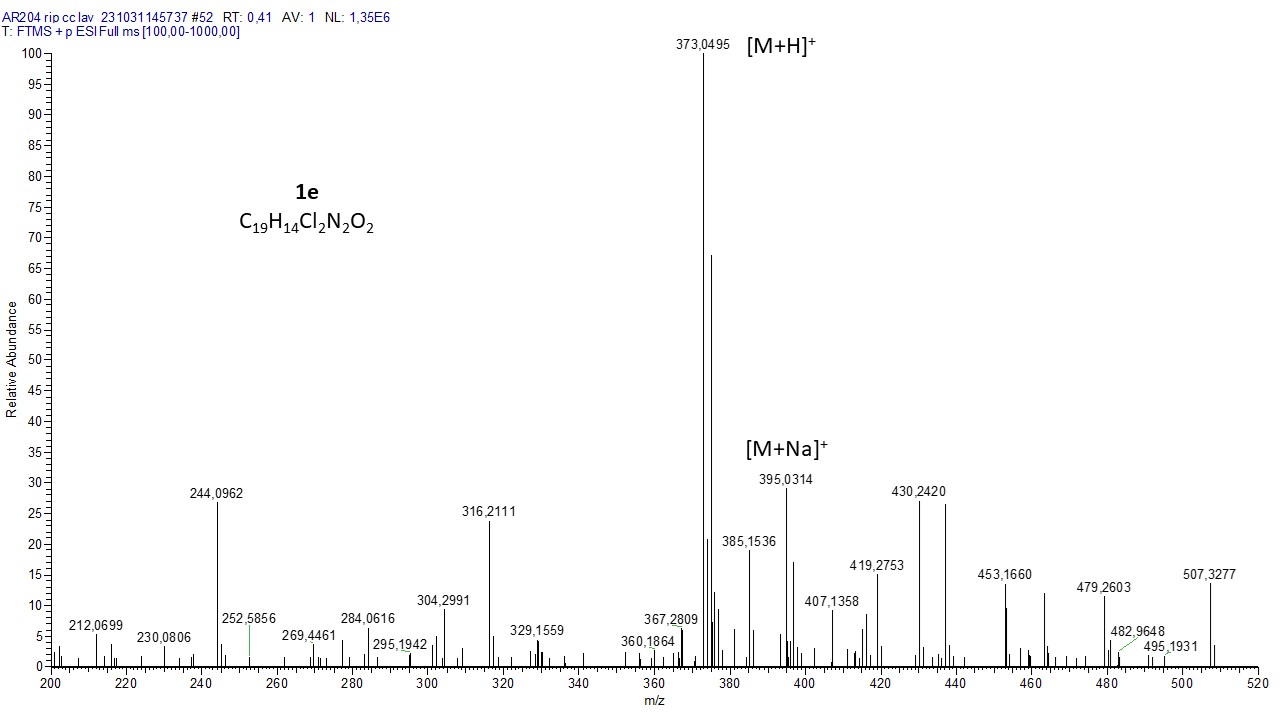


# **FIGURE S30.** HRMS (ESI) spectrum of compound **1e**.

**
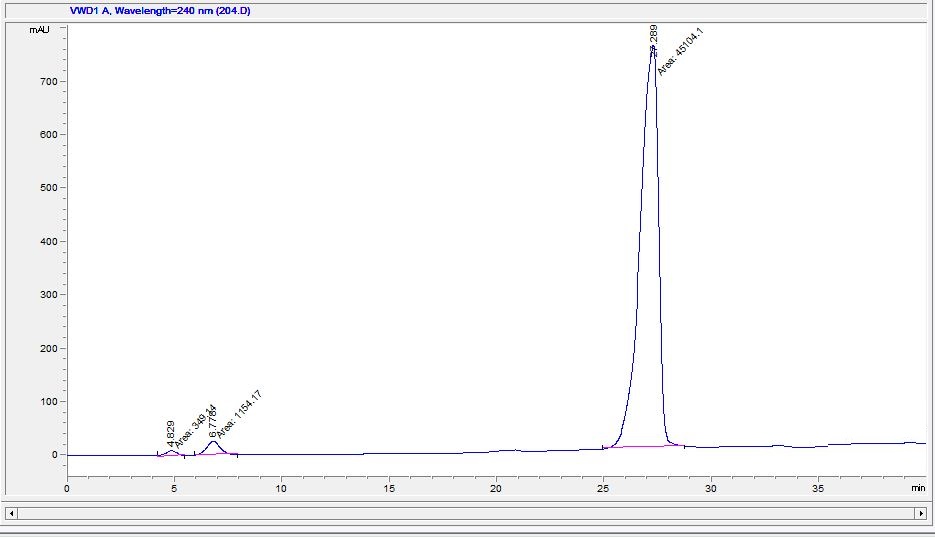
**

**
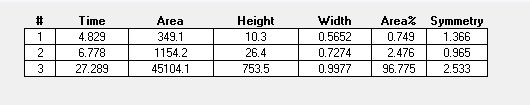
**

# **FIGURE S31.** HPLC analysis of compound **1e**.


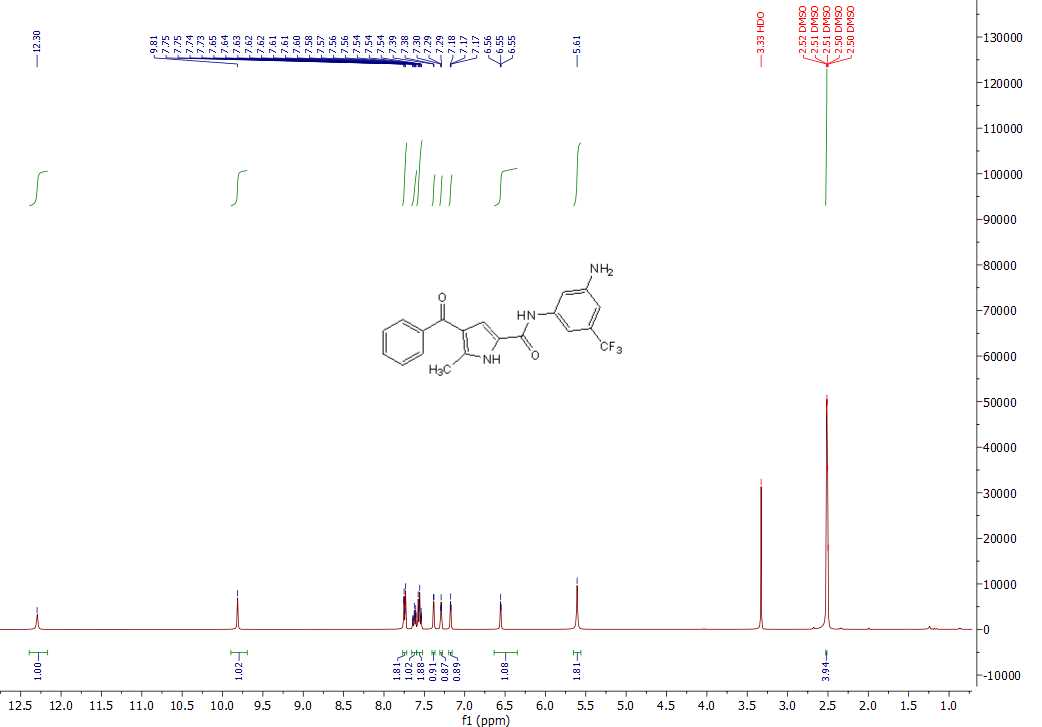


# **FIGURE S32.** ^1^H NMR spectrum (400 MHz, DMSO-*d_6_*) of compound **1f**.


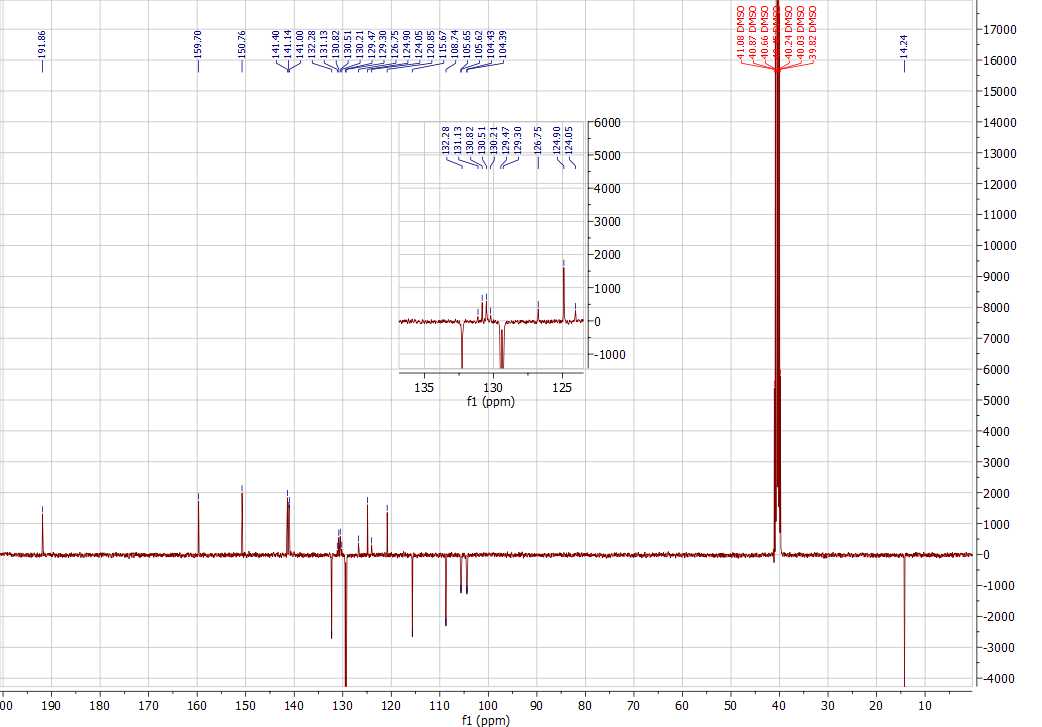


# **FIGURE S33.** ^13^C-DEPTq NMR spectrum (100 MHz, DMSO-*d*_6_) of compound **1f**.


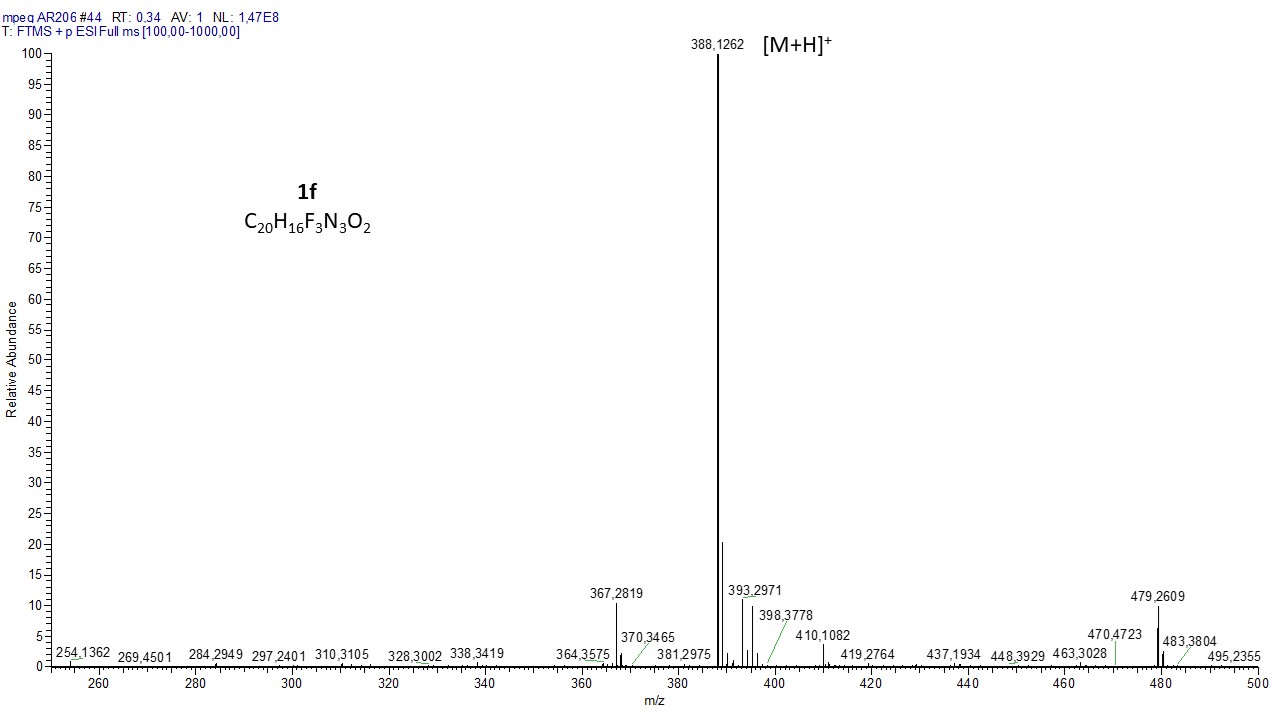


# **FIGURE S34.** HRMS (ESI) spectrum of compound **1f**.

**
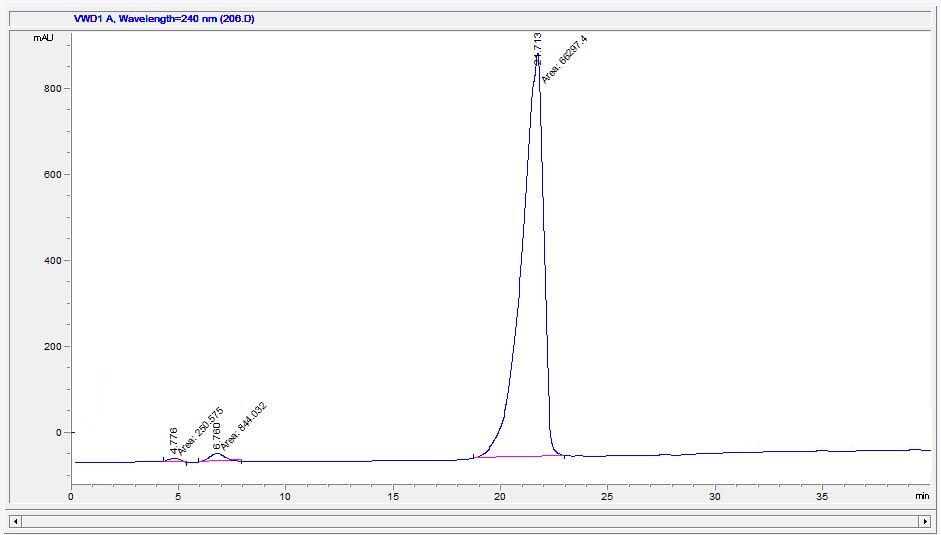
**

**
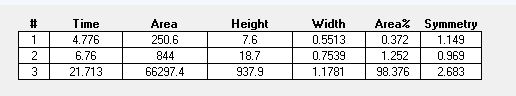
**

# **FIGURE S35.** HPLC analysis of compound **1f**.


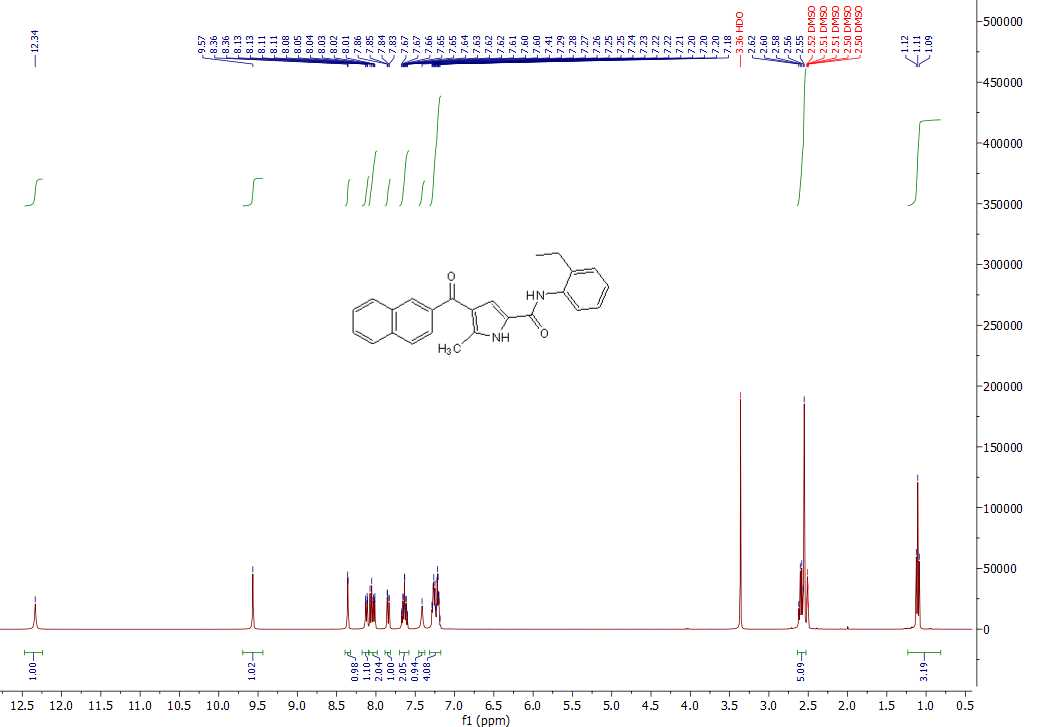


# **FIGURE S36.** ^1^H NMR spectrum (400 MHz, DMSO-*d_6_*) of compound **2a**.


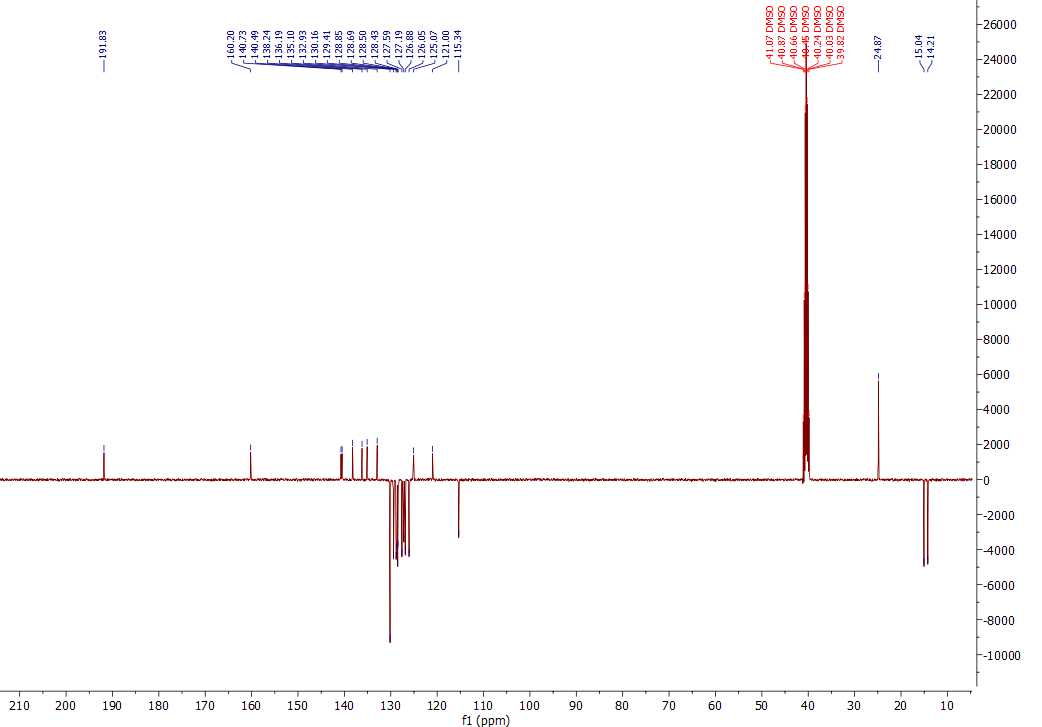


# **FIGURE S37.** ^13^C-DEPTq NMR spectrum (100 MHz, DMSO-*d*_6_) of compound **2a**.


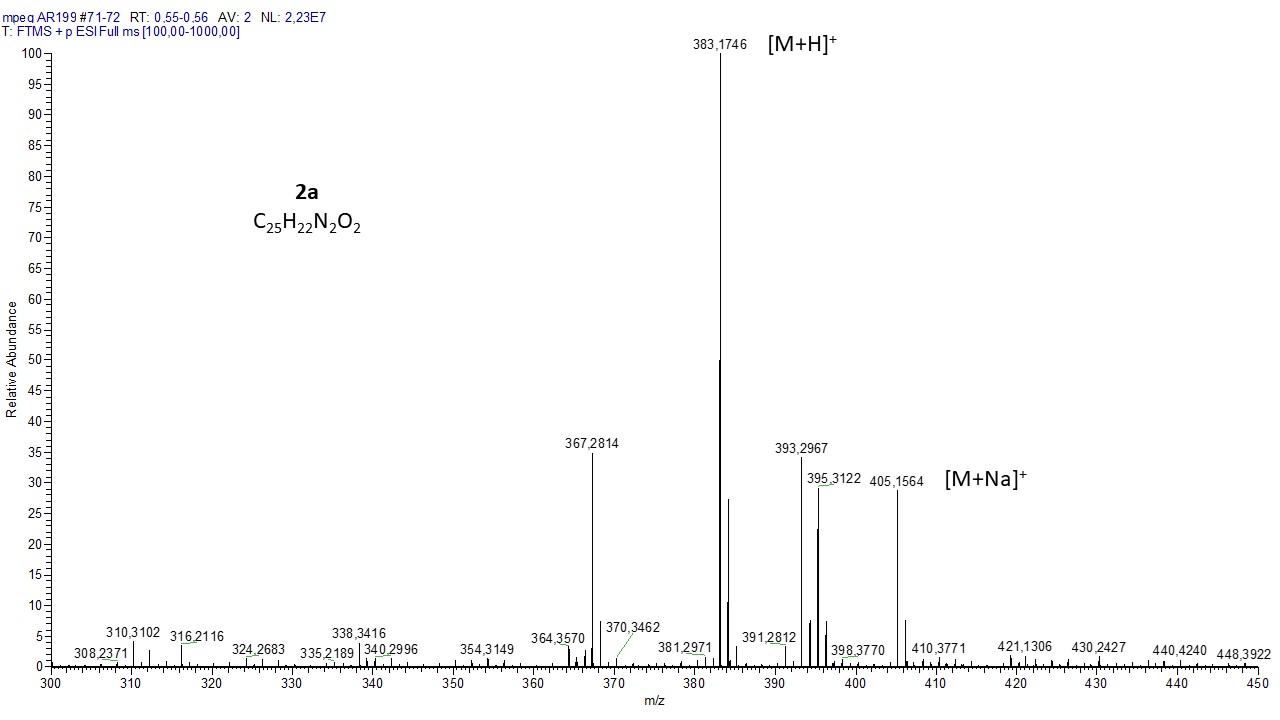


# **FIGURE S38.** HRMS (ESI) spectrum of compound **2a**.


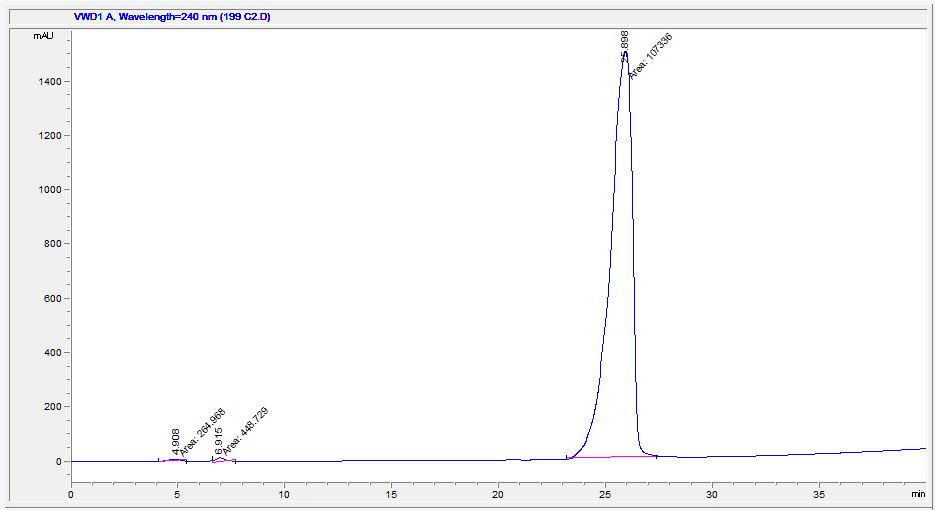


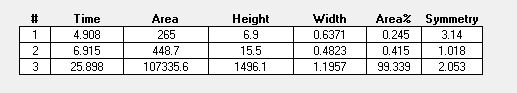


# **FIGURE S39.** HPLC analysis of compound **2a**.


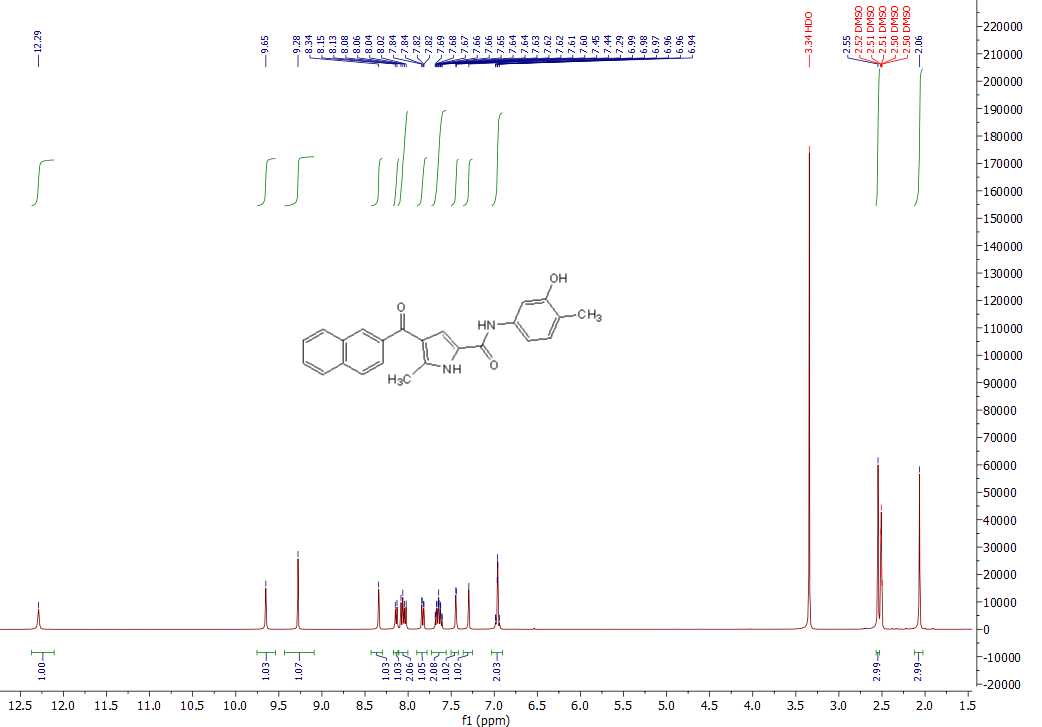


# **FIGURE S40.** ^1^H NMR spectrum (400 MHz, DMSO-*d*_6_) of compound **2b**.


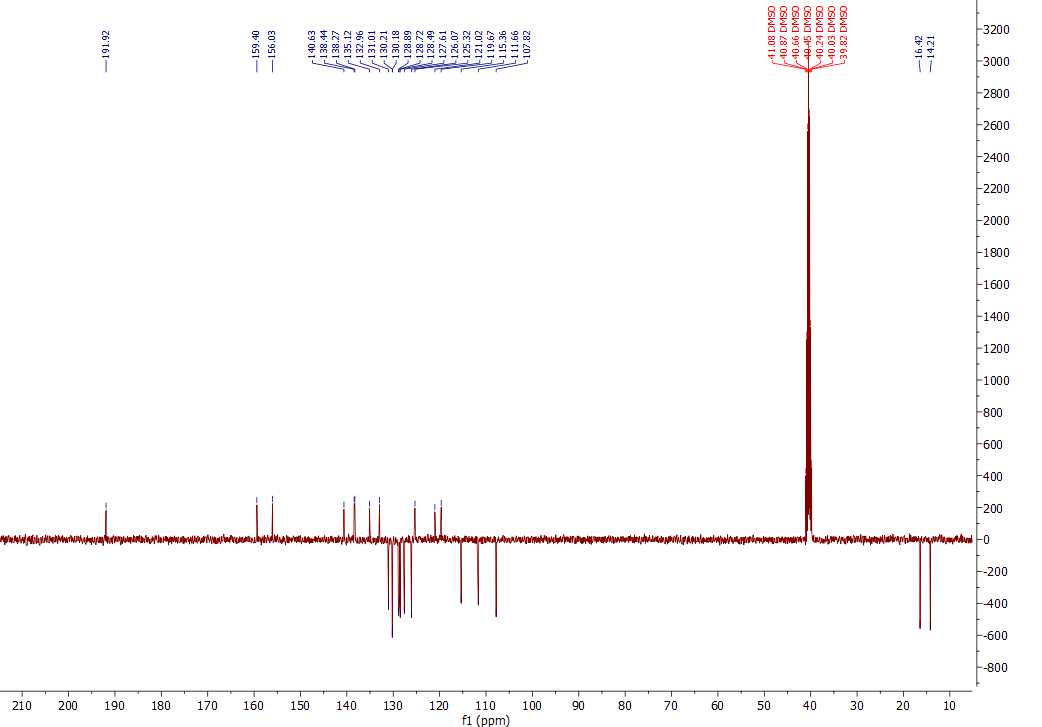


# **FIGURE S41.** ^13^C-DEPTq NMR spectrum (100 MHz, DMSO-*d*_6_) of compound **2b**.


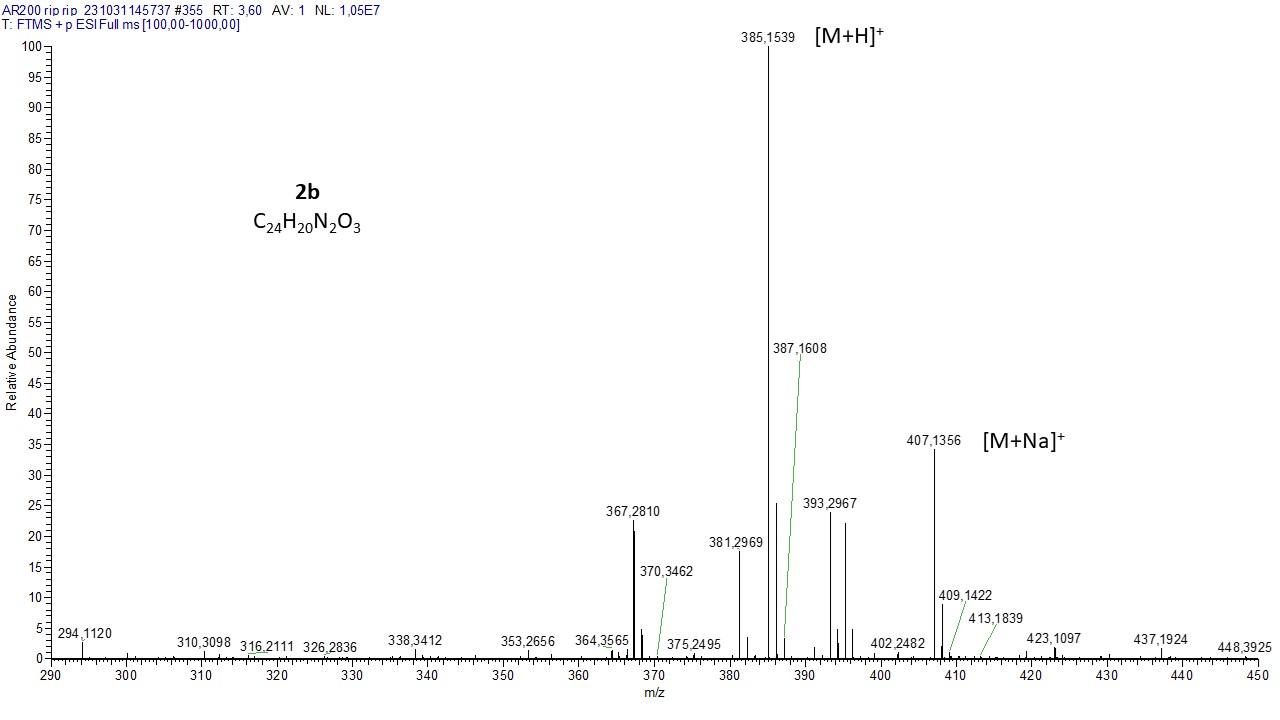


# **FIGURE S42.** HRMS (ESI) spectrum of compound **2b**.


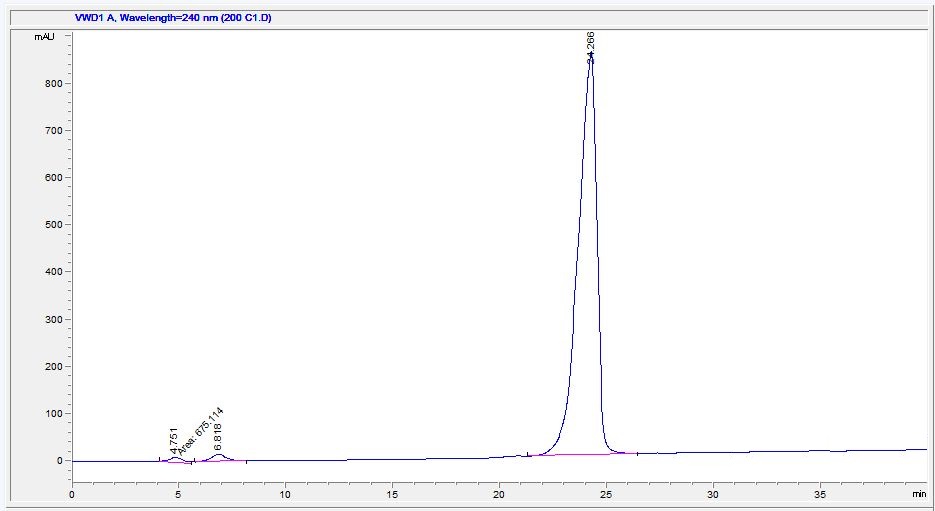


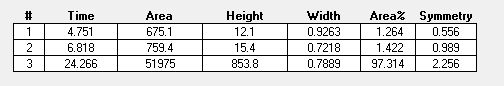


# **FIGURE S43.** HPLC analysis of compound **2b**.


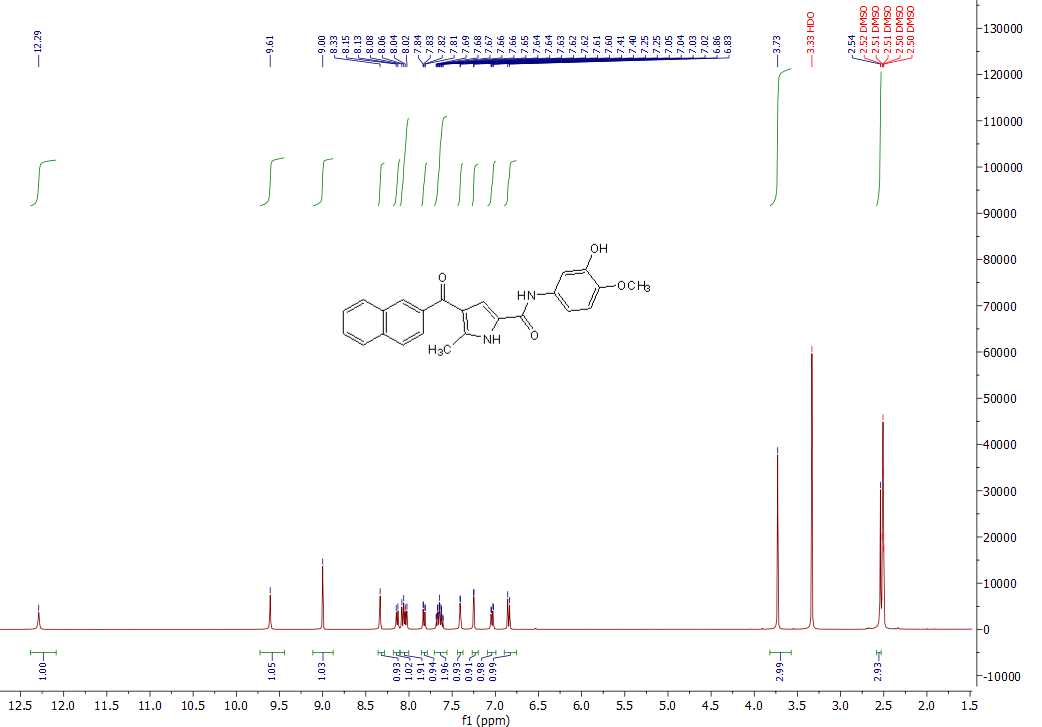


# **FIGURE S44.** ^1^H NMR spectrum (400 MHz, DMSO-*d_6_*) of compound **2c**.


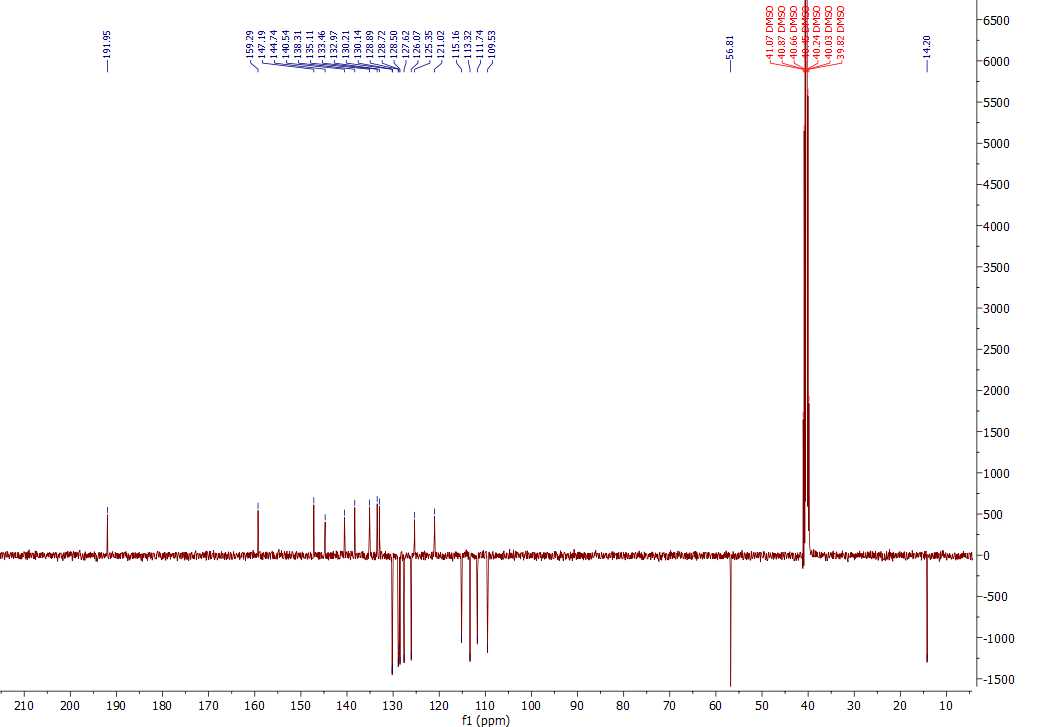


# **FIGURE S45.** ^13^C-DEPTq NMR spectrum (100 MHz, DMSO-*d*_6_) of compound **2c**.


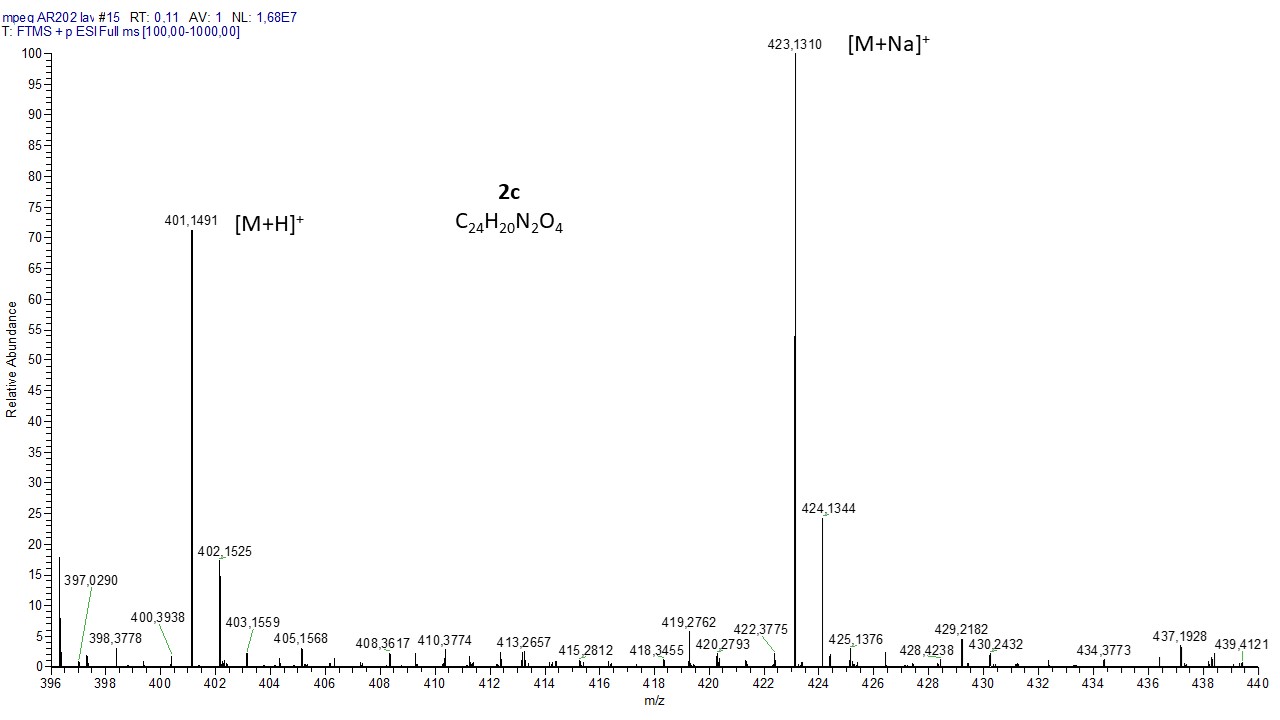


# **FIGURE S46.** HRMS (ESI) spectrum of compound **2c**.


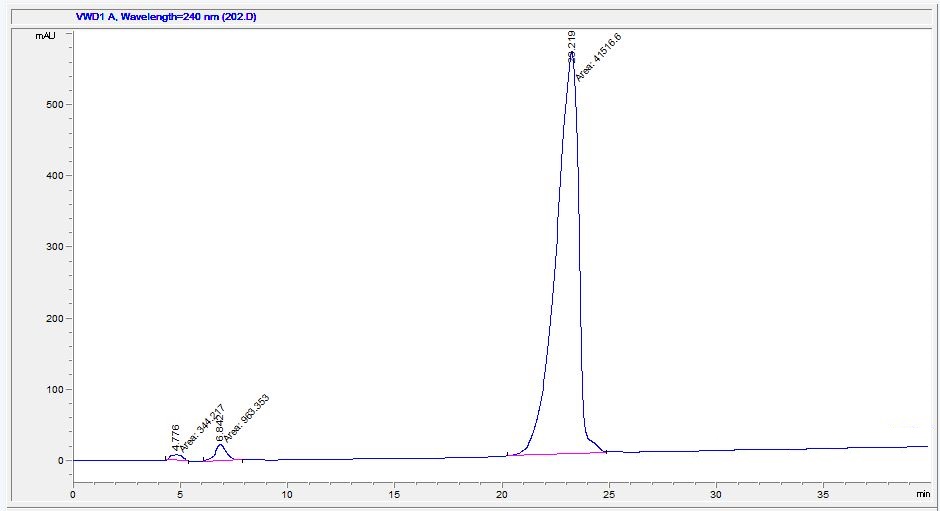


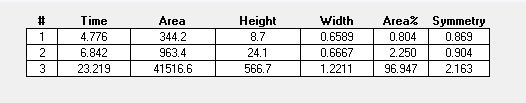


# **FIGURE S47.** HPLC analysis of compound **2c**.


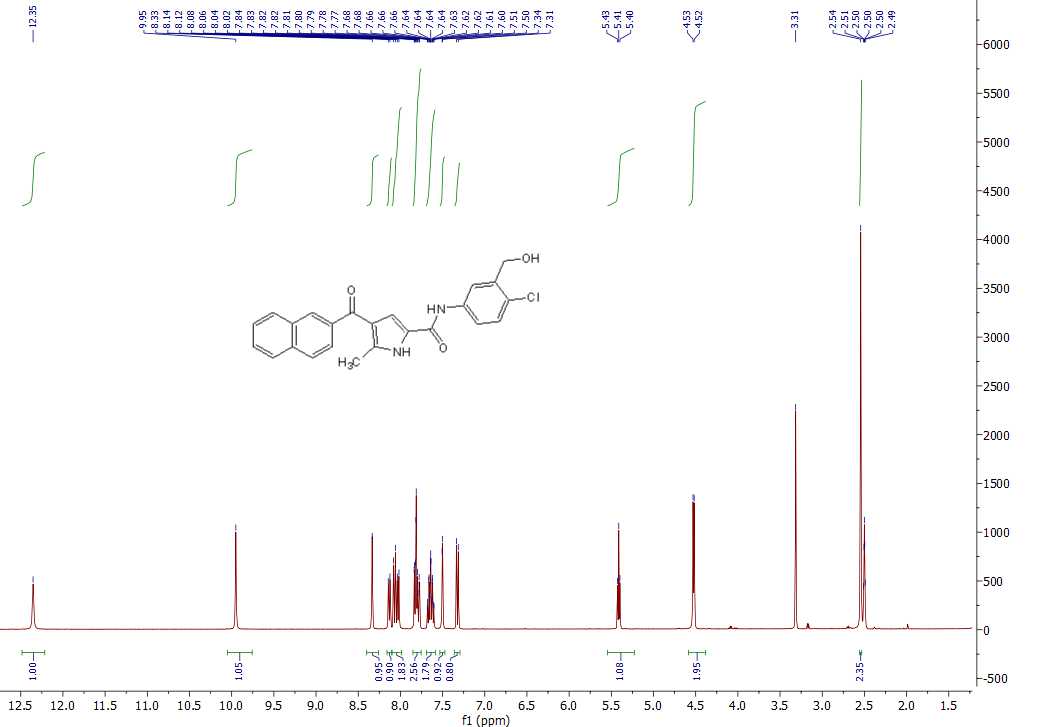


# **FIGURE S48.** ^1^H NMR spectrum (400 MHz, DMSO-*d_6_*) of compound **2d**.


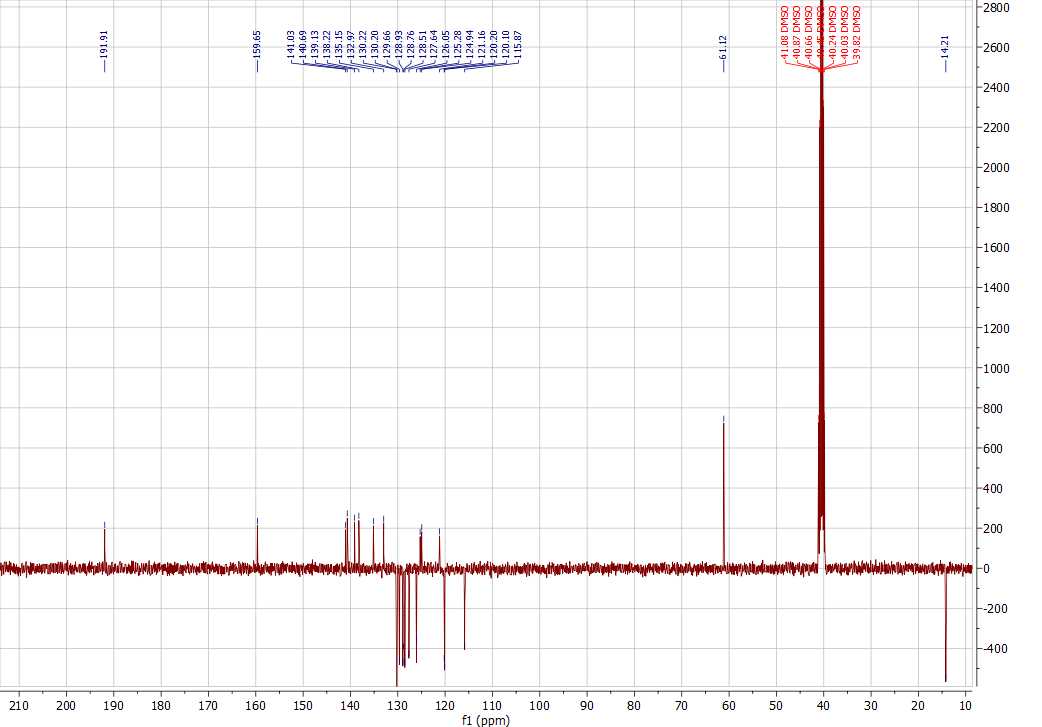


# **FIGURE S49.** ^13^C-DEPTq NMR spectrum (100 MHz, DMSO-*d*_6_) of compound **2d**.


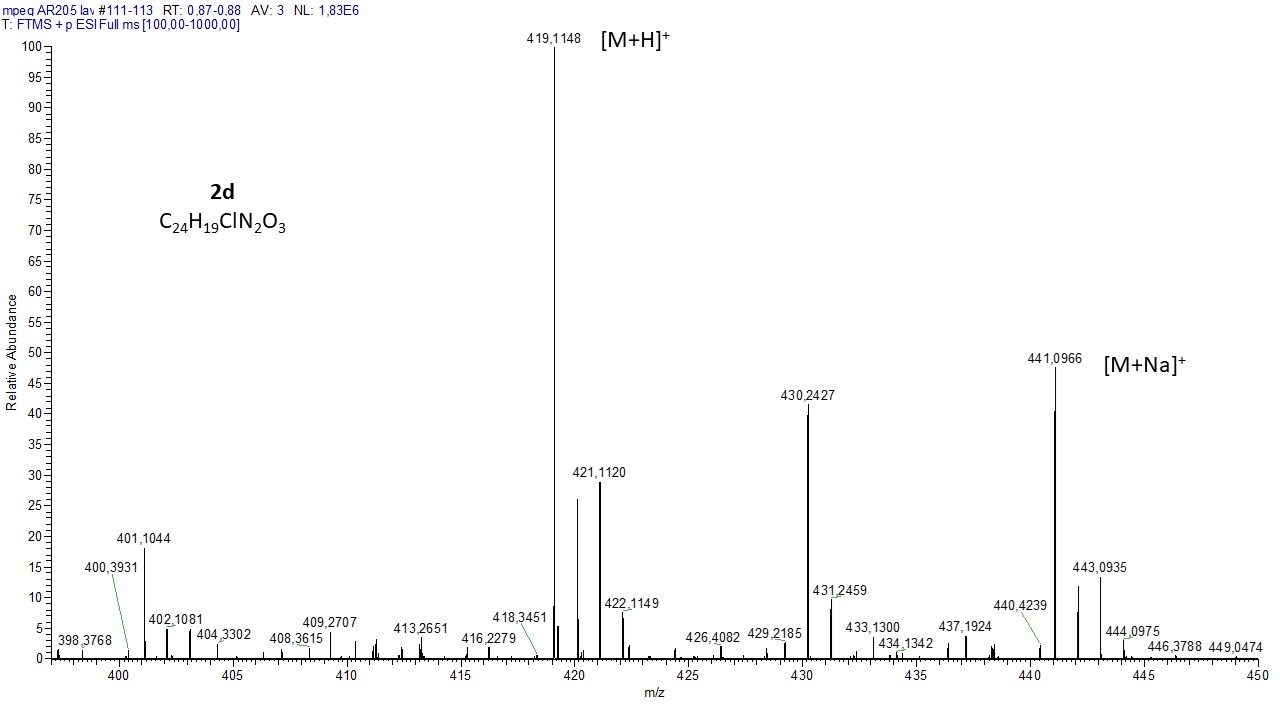


# **FIGURE S50.** HRMS (ESI) spectrum of compound **2d**.


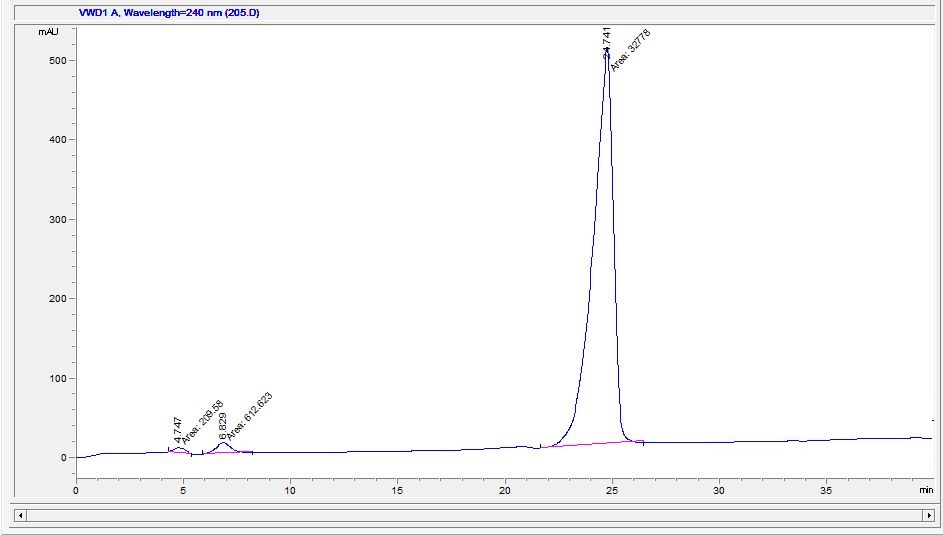


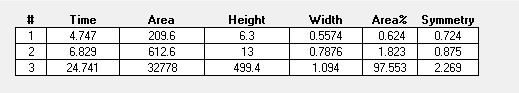


# **FIGURE S51.** HPLC analysis of compound **2d**.


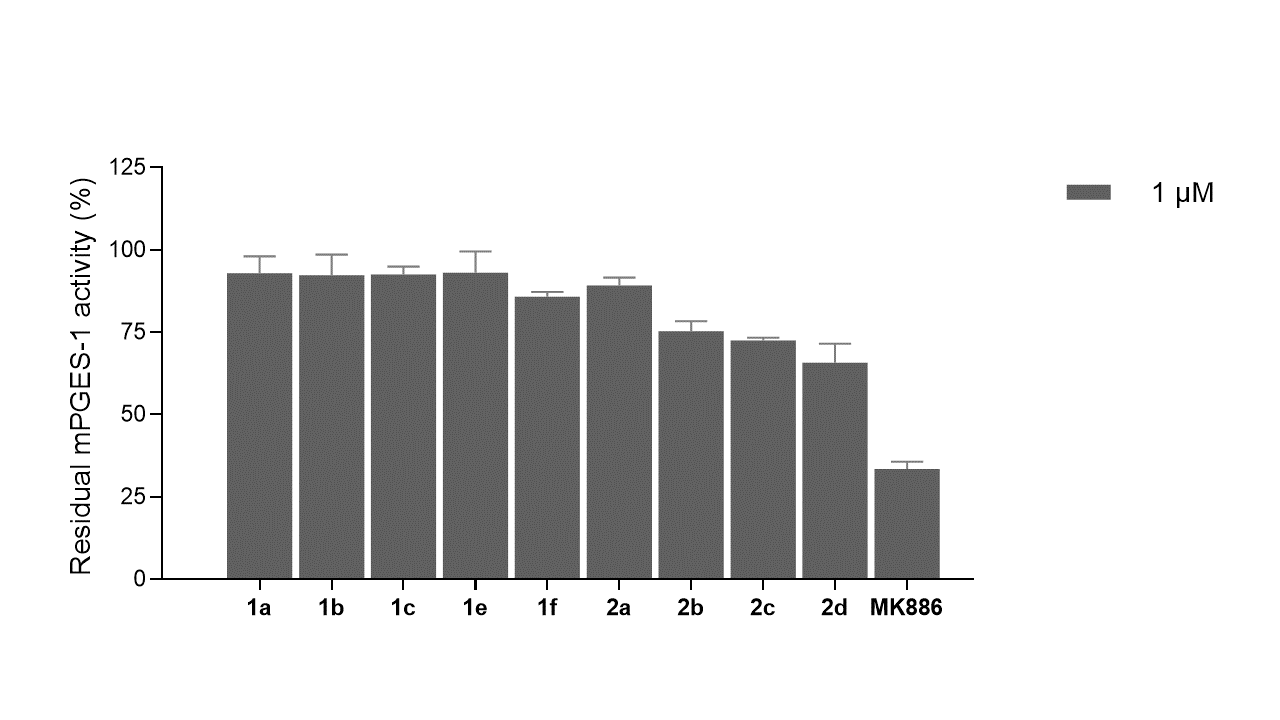


# **FIGURE S52.** Residual activity of mPGES-1(%) after incubation with compounds **1a**-**f** and **2a**-**d** at a concentration of 1 μM.


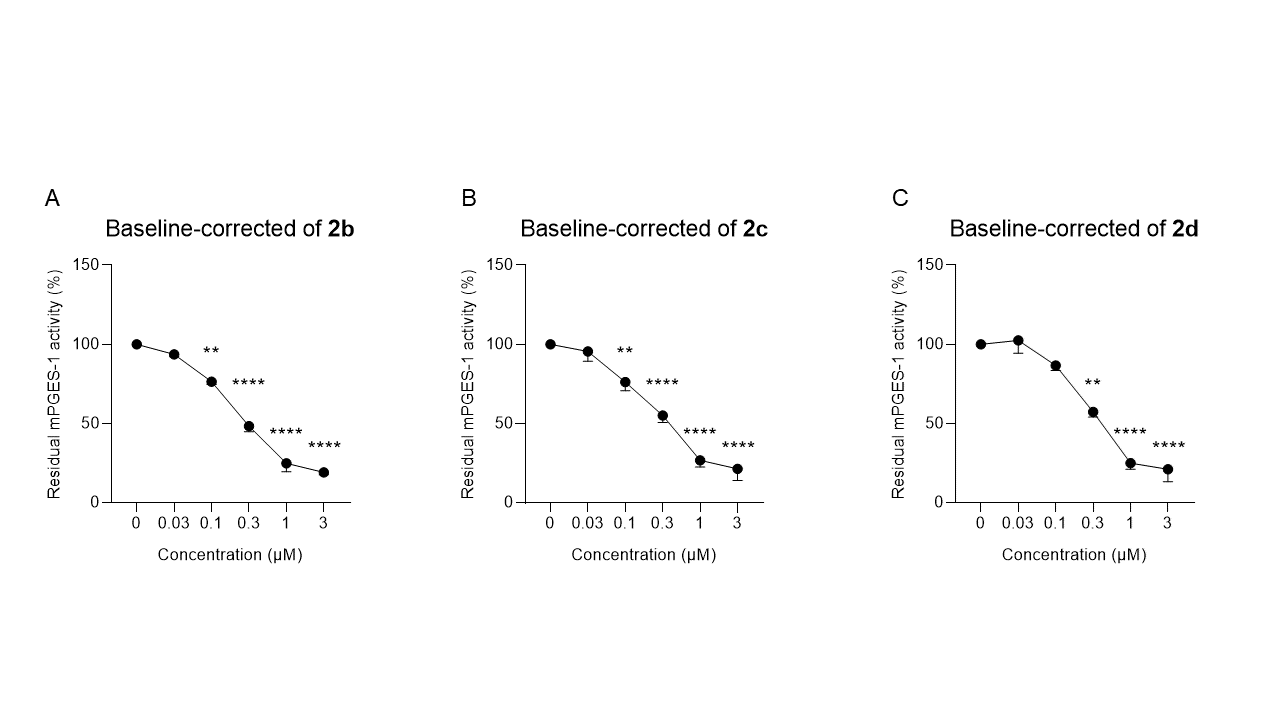


# **FIGURE S53.** IC_50_ curves for compounds **2b**(A), **2c**(B), **2d**(C) on mPGES-1 Data are expressed as means of the three experiments ± SD.


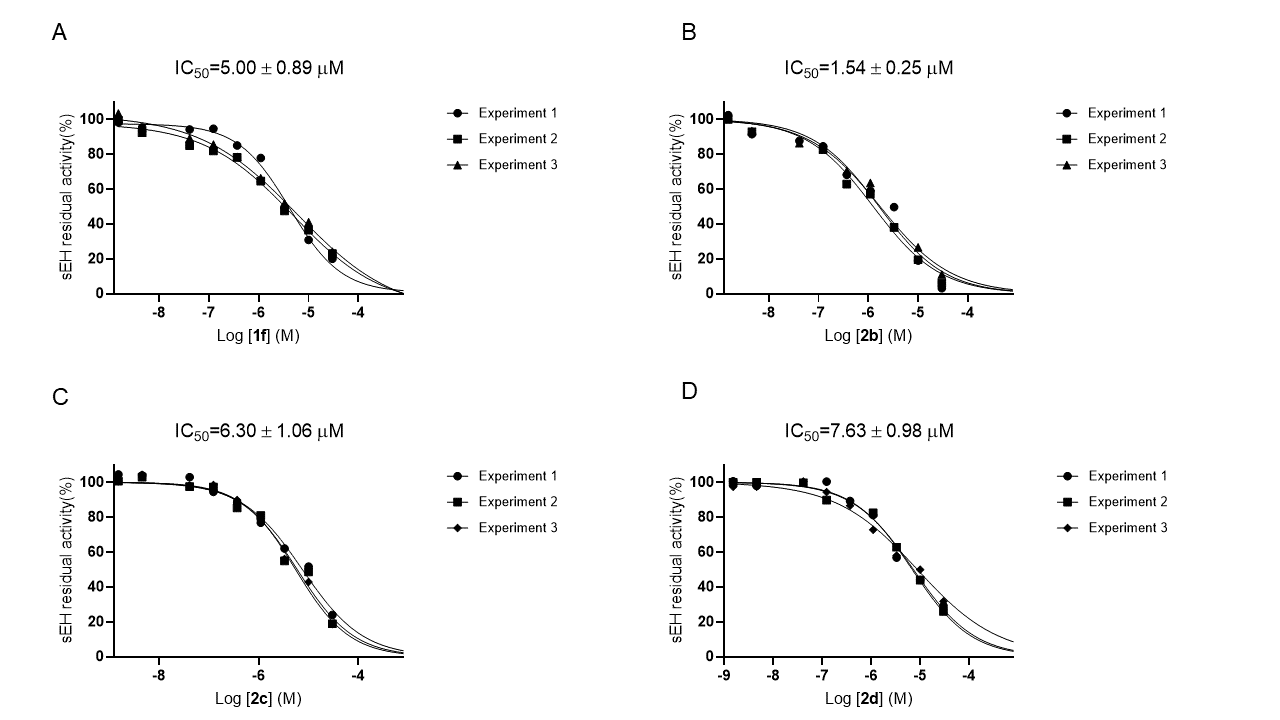


# **FIGURE S54.** IC_50_ curves for compounds **1f**(A), **2b**(B), **2c**(C), **2d**(D) on sEH. Data are expressed as means of the three experiments ± SD.

# **TABLE S2.** H-bonds and π-π interactions reported for **1f**, **2b**-**2d** in the mPGES-1 and sEH binding sites.

|  | **mPGES-1** | | **sEH** | |
| --- | --- | --- | --- | --- |
| **Compounds** | **H-bonds** | **π-π interactions** | **H-bonds** | **π-π interactions** |
| **1f** | -Chain A: Gln134, Tyr130, Ser 127, Arg126.  -Chain B: Gln36. | \ | Phe267. | Tyr383, His524, Trp336. |
| **2b** | -Chain A: Ser127, Arg126.  -Chain B: Gln36. | -Chain A: Tyr130.  -Chain B: His53. | Phe267, Tyr383, Asp335. | His524, Trp336. |
| **2c** | -Chain A: Ser127, Arg126.  -Chain B: Gln36. | -Chain A: Tyr130.  -Chain B: His53, Phe44. | Tyr466 | Tyr383, His524, Trp336 |
| **2d** | -Chain A: Thr131, Ser127, Arg126.  -Chain B: Gln36. | -Chain A: Tyr130.  -Chain B: His53, Phe44. | Phe267, Tyr383, His524, Asp335 | His524, Trp336 |

# **REFERENCES**

1. A. Wiegard, W. Hanekamp, K. Griessbach, J. Fabian, M. Lehr, *Eur.J. Med. Chem.* **2012,** *48*, 153.
